# Supplementary material for: Replicability of bulk RNA-Seq differential expression and enrichment analysis results for small cohort sizes
Source: PLoS Comput Biol. 2025 May 5;21(5):e1011630. doi: 10.1371/journal.pcbi.1011630 (PMC12077797; doi:10.1371/journal.pcbi.1011630)
Supplement: S1 Text — Table A: Statistical tests used for differential expression analysis. Table B: Comparison of Cui et al. [14] with this study. Fig A: Performance metrics for different statistical tests. Fig B: Influence of subsampling with replacement on replicability. Fig C: Influence of subsample inclusion in ground truth on precision and recall. Figs D–F: Partial results with Wilcoxon signed-rank test. Fig G: DEGs from 8 permuted and unpermuted data sets. (PDF) [file pcbi.1011630.s001.pdf]

Replicability of bulk RNA-Seq differential expression and enrichment analysis results for small cohort sizes

Supporting Information 1

Peter Methys Degen and Matúš Medo

April 15, 2025

Contents

1 Additional Tables **1**

2 Additional Figures **2**
2.1 Comparison of DEG tests 2
2.2 Influence of subsampling with replacement on replicability 3
2.3 Influence of subsample inclusion in ground truth on precision and recall 4
2.4 Wilcoxon signed-rank test 6

3 Derivation of expected metrics under random gene selection **9**

1 Additional Tables

Table A: All statistical methods for differential expression testing used in this study. QLF: quasi-likelihood F-test; LRT: likelihood-ratio test; TREAT: t-test relative to a threshold. See method section of main text for an explanation of TREAT.

Test	Tool	Null hypothesis	Post hoc FC threshold
Wald	DESeq2	$\lvert\log_2 \mathrm{FC}\rvert = 0$	$\lvert\log_2 \mathrm{FC}\rvert > t_{post} \in \{0, 1\}$
Wald	DESeq2	$\lvert\log_2 \mathrm{FC}\rvert \leq 1$	None
LRT	edgeR	$\lvert\log_2 \mathrm{FC}\rvert = 0$	$\lvert\log_2 \mathrm{FC}\rvert > t_{post} \in \{0, 1\}$
TREAT / LRT	edgeR	$\lvert\log_2 \mathrm{FC}\rvert \leq 1$	None
QLF	edgeR	$\lvert\log_2 \mathrm{FC}\rvert = 0$	$\lvert\log_2 \mathrm{FC}\rvert > t_{post} \in \{0, 1\}$
TREAT / QLF	edgeR	$\lvert\log_2 \mathrm{FC}\rvert \leq 1$	None

Table B: Comparison of Cui et al. (2021) with the DEG replicability study presented in this paper.

	**Cui et al.**	**This study**
Data sets	3	18
Cohort sizes	3–14,18,24	3–15
Cohorts per scenario	4	100
Cohorts can share replicates	No	Yes
Design	Unpaired	Paired, controlled
Tool	edgeR	edgeR, DESeq2
Statistical test	Unspecified	QLF, LRT, Wald, TREAT
FDR threshold	0.05	0.05
$\lvert\log_2 \mathrm{FC}\rvert$ threshold	1	0 and 1
FC threshold strategy	Unspecified	Formal test, post hoc
Replicability metric	Jaccard	Jaccard
Ground truth cohort size	14,18,24	58.5 (median), 39–161 (range)

2 Additional Figures

2.1 Comparison of DEG tests

As described in the main text, we use the replicability, precision, and recall as our primary metrics to evaluate the performance of results derived from subsampled cohorts. It should be noted that these metrics are non-zero, on average, also when the significant genes are chosen at random. Therefore, as a benchmark, we introduce the expected random gene replicability as

$$R_0 \approx \frac{|S_i||S_j|}{N_{tot}(|S_i| + |S_j|) - |S_i||S_j|}, \tag{1}$$

where N_{tot} is the total number of tested genes or terms/pathways. Rather than calculating this value for every pair (i, j), we calculate a median-based expected replicability by setting $|S_i| = |S_j| = \text{median}\big[|S_i| \mid i \in \{1, 2, \ldots, 100\}\big]$. The expected precision and recall values for randomly selected genes are computed in a similar manner. A derivation of all relevant formulas is given in Section 3.

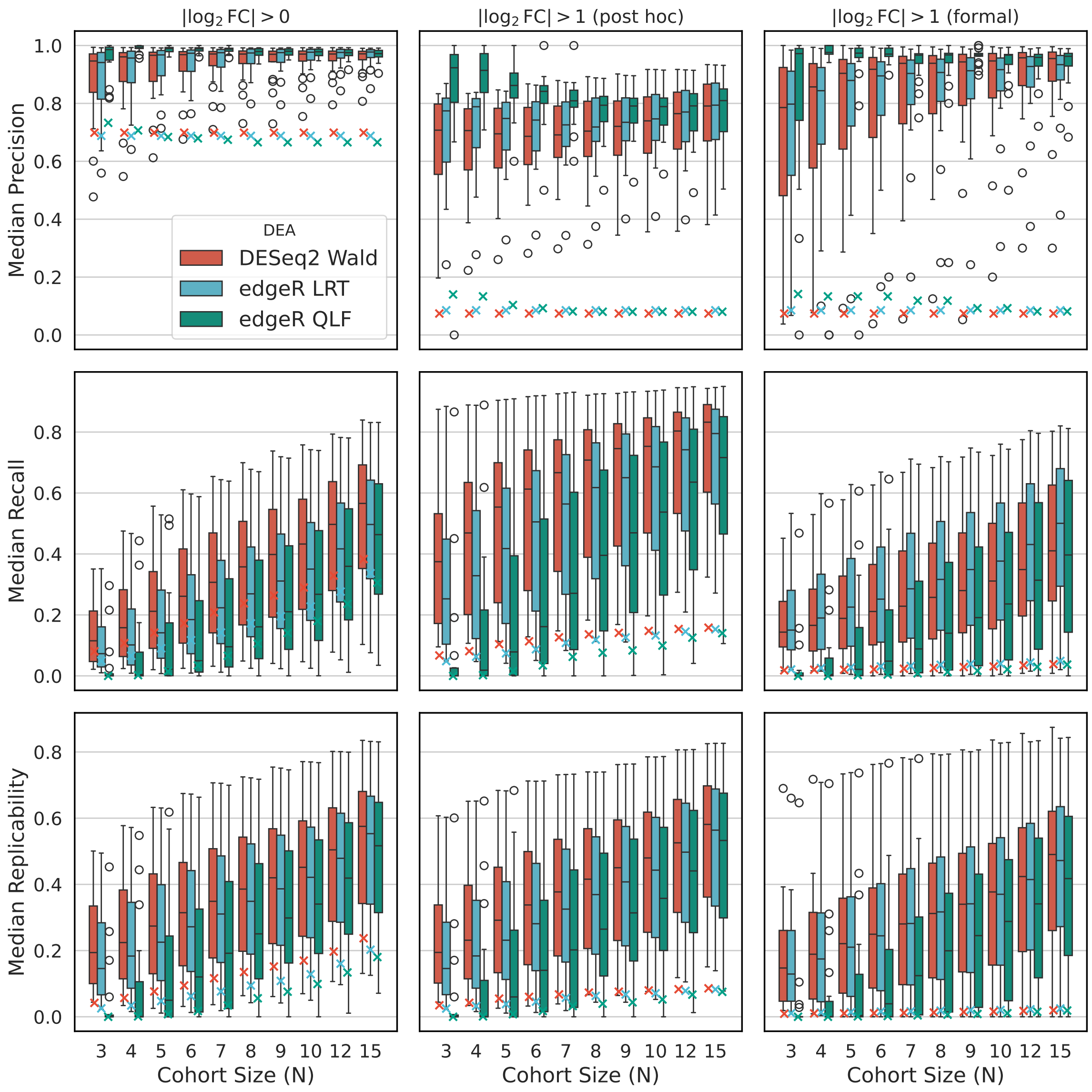

Figure A: **Performance metrics for different statistical tests (Wald, LRT, QLF).** Each box plot summarizes 18 data points that represent the median values obtained using 100 subsampled cohorts for each of the data sets. Outlier data sets are shown as circles. The left column shows results for DEGs defined without a logFC threshold. The middle column defines DEGs by taking the DEGs from the left column and applying a post hoc filter of $|\log_2 \mathrm{FC}| > 1$. The right column shows results with a formal threshold of $|\log_2 \mathrm{FC}| > 1$. The crosses show the median expected metrics under the null model of random gene selection, averaged over all data sets.

2.2 Influence of subsampling with replacement on replicability

As our methodology relies on subsampling cohorts from a parent data set, it is instructive to investigate the effect of repeated replicates when calculating the replicability from pairs of cohorts. We stress again that the cohorts are subsampled without replacement, so there are no repeated replicates within any given cohort. However, each cohort is subsampled from the full parent data, which means that there can be repeated replicates between different cohorts. Repetition of replicates can be expected to increase replicability metrics by a small amount. This can be checked explicitly by computing the metrics only for those cohort pairs that have no mutually repeating replicates. Panel A in Fig B shows the fraction of thus-defined unique cohort pairs, relative to all $\binom{100}{2} = 4950$ pairs (per cohort size, for 7 of 18 data sets). This fraction shrinks to 30% for the most extreme case (COAD $N = 15$), indicating that most pairs have

repeated replicates. However, even in this case, the repeated replicates inflate our replicability metrics by a negligible amount. This can be seen in panel B, which shows the difference in the median replicability computed from all pairs and only from unique pairs. The differences are on the order of 0.2–1.3%.

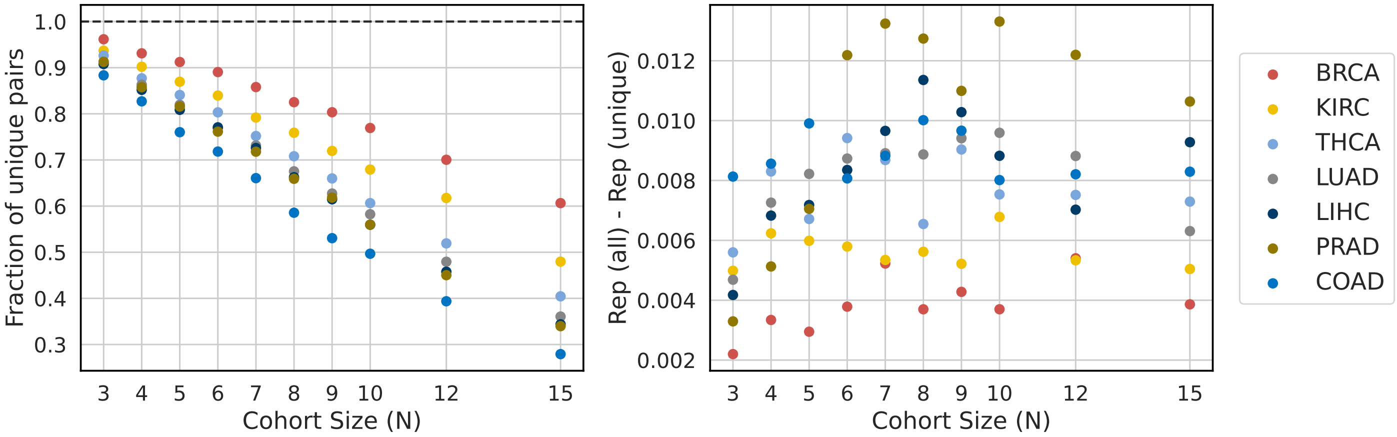

Figure B: **Replicability inflation.** *Left:* Fraction of cohort pairs without repeated replicates out of 4'950 total pairs (per data set per N). *Right:* Median replicability calculated from all pairs minus median replicability calculated from unique pairs without repeated replicates.

2.3 Influence of subsample inclusion in ground truth on precision and recall

A similar consideration has to be made for our ground truth definition. Since a given subsample itself contributes to the ground truth definition from which we calculate our performance metrics, there is a degree of circularity in the analysis. However, when the parent data sets are sufficiently much larger than the subsampled data sets, this effect is negligible. An alternative strategy that entirely avoids circularity would be to exclude the subsample cohort from the ground truth definition, yielding separate ground truths for each subsampled cohort (similar to the technique of cross-validation in statistics). However, such a study design would require substantially more computing resources, which is likely not worth the effort given the minimal expected gain.

To illustrate this, we consider four data sets: COAD (smallest data set with $N_{full} = 39$), SNF2 (best performing and second smallest data set with $N_{full} = 42$), HSPL (third smallest data set with $N_{full} = 43$, and LMAB (worst performing data set with $N_{full} = 161$). Fig C compares the number of DEGs in the full ground truth (DESeq2 with formal threshold $|\log_2 \mathrm{FC}| > 1$) with the number of DEGs obtained from reduced ground truths by removing $N \in \{15, 9, 3\}$ samples from the full data sets. The Jaccard indices between these two ground truth definitions is consistently above 0.8, except for the most extreme case of COAD $N = 15$, where it is 0.78.

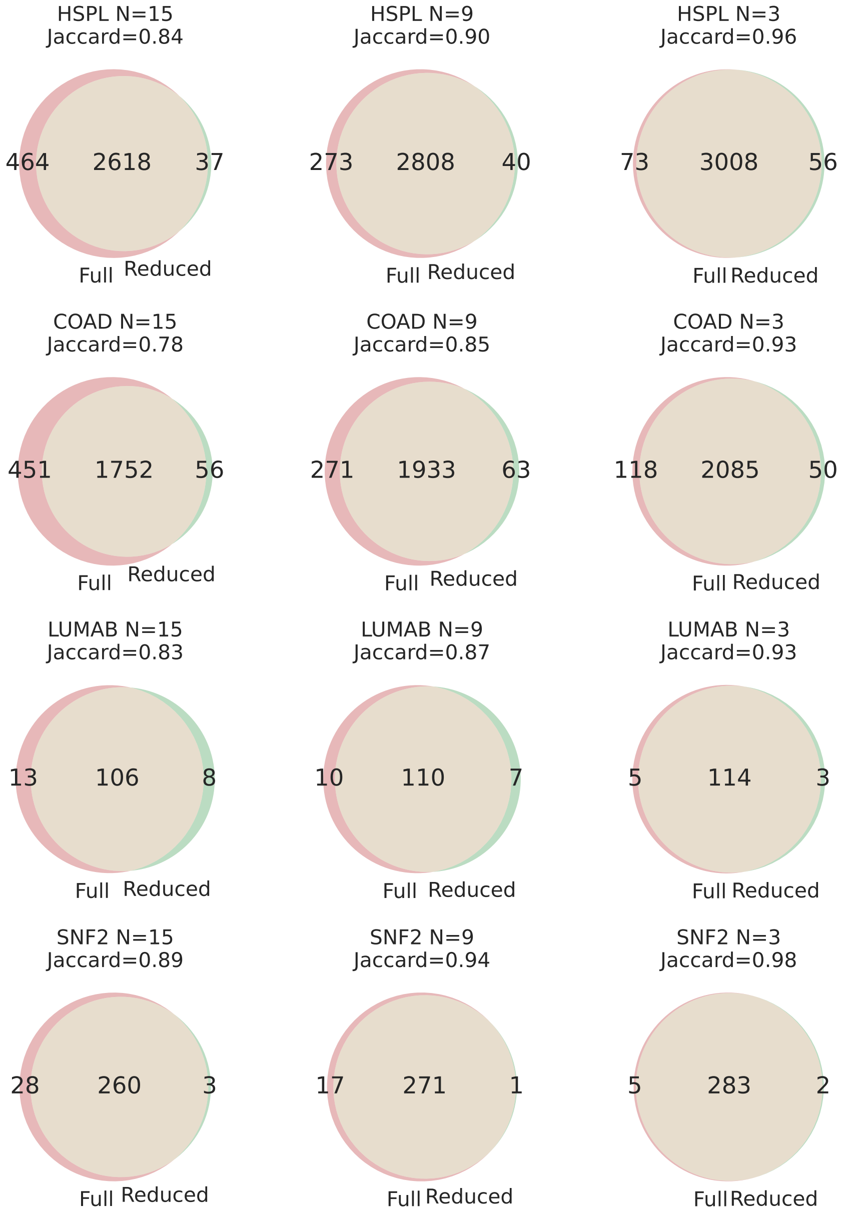

Figure C: **Venn diagrams showing the number of DEGs for full and reduced ground truths.** Reduced ground truths (green) are obtained by removing N random replicates from the full ground truth (red). The numbers are averaged over 10 trials.

2.4 Wilcoxon signed-rank test

A recent study on population-level RNA-Seq studies with large sample sizes [1] recommends the use of the non-parametric Wilcoxon rank-sum test over edgeR and DESeq2 if the number of samples is larger than $N = 7$. We wanted to see how well this recommendation applies to our 8 normal-tumor TCGA data sets and present here the results for the Wilcoxon signed-rank test (the paired-sample equivalent of the rank-sum test). As the Wilcoxon test is a generic test not specifically designed for RNA-Seq data, we used DESeq2 for count data normalization and fold change estimation (the latter is needed for post hoc fold change thresholding). Although the precision achieved by the Wilcoxon test is comparable to that of the other methods (Fig D), the recall is low, as the median number of DEGs is zero for $N \leq 8$ (Fig E). We also computed the Matthew's Correlation Coefficient (MCC) [2], which is a balanced performance metric that incorporates all four categories of the confusion matrix and ranges from -1 (worst) to $+1$ (best) and is defined as

$$\mathrm{MCC} = \frac{\mathrm{TP} \times \mathrm{TN} - \mathrm{FP} \times \mathrm{FN}}{\sqrt{(\mathrm{TP}+\mathrm{FP})(\mathrm{TP}+\mathrm{FN})(\mathrm{TN}+\mathrm{FP})(\mathrm{TN}+\mathrm{FN})}}.$$

Fig F shows that the Wilcoxon has the lowest MCC in our sample size range. For this reason, in addition to the difficulties with formal thresholding and adjusting for confounders, we decided to avoid further using this test for the main text.

The authors of [1] also reported that DEGs are found by edgeR in permuted data where DEGs should be absent by definition. We assessed this on our normal–tumor cancer data sets and found that it happens rarely for small cohort sizes and the number of such spurious DEGs is small even for large cohorts (Fig G).

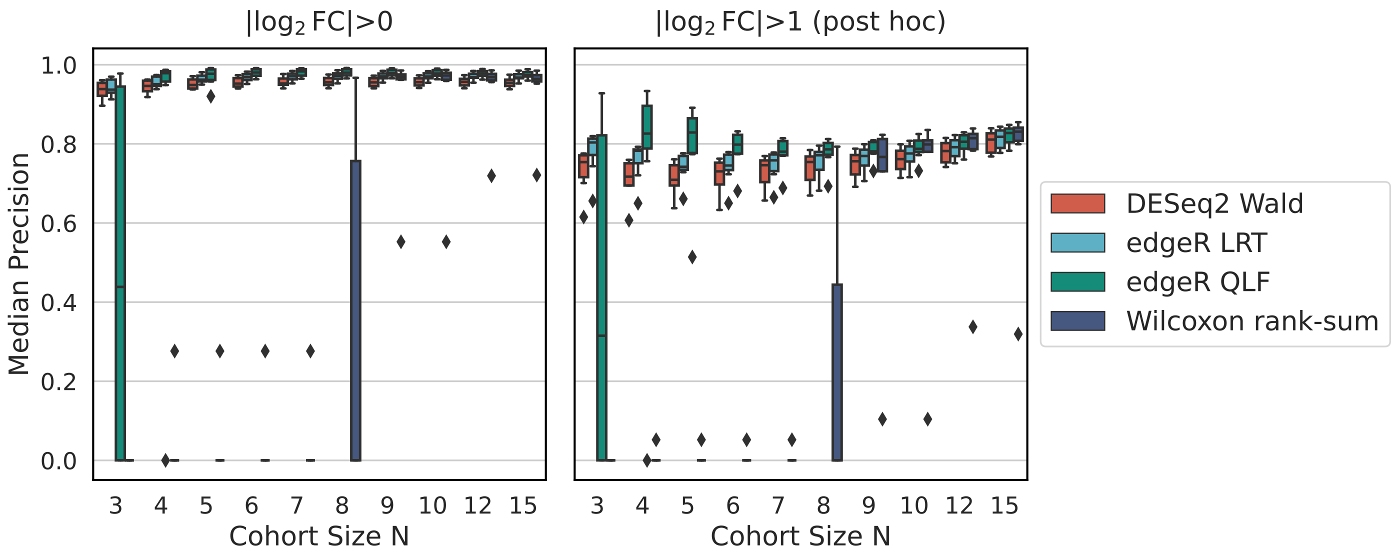

Figure D: Precision results with added Wilcoxon test. Each boxplot summarizes the eight normal–tumor tissue comparisons. (Side note: For legacy reasons, we use a different definition of precision here compared to the other figures: instead of removing the precision from summary calculations like the median when the precision is undefined, we set it to 0. This mainly affects the QLF and Wilcoxon tests when cohort sizes are very small, in which case most cohorts yield 0 DEGs.)

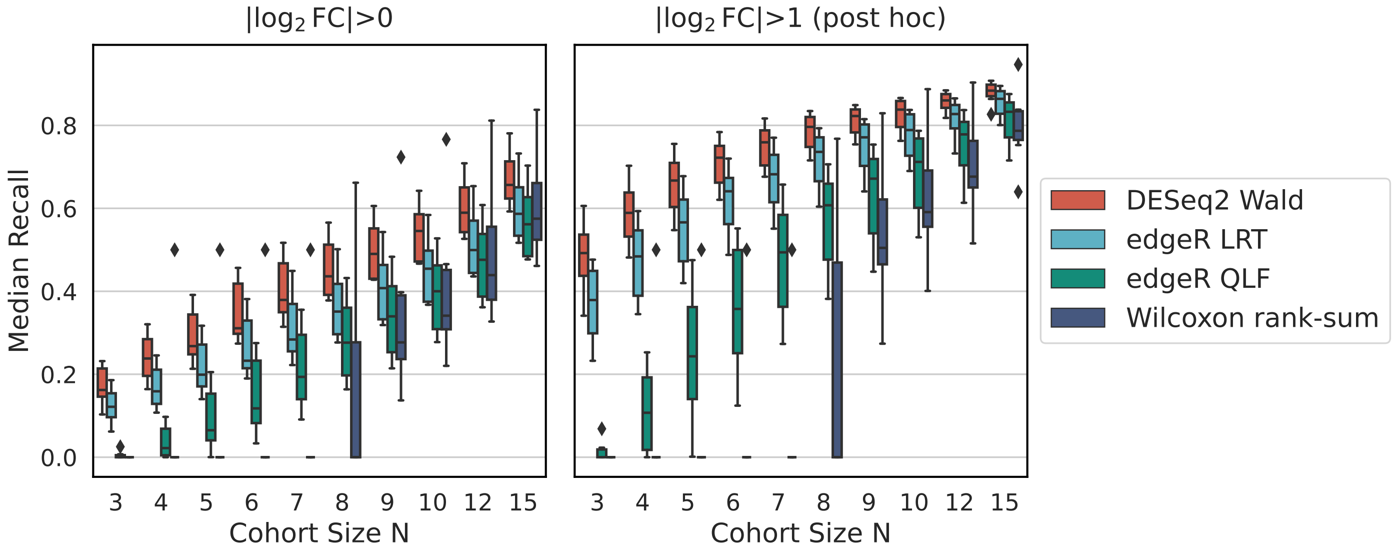

Figure E: Recall results with added Wilcoxon test. Each boxplot summarizes the eight normal–tumor tissue comparisons.

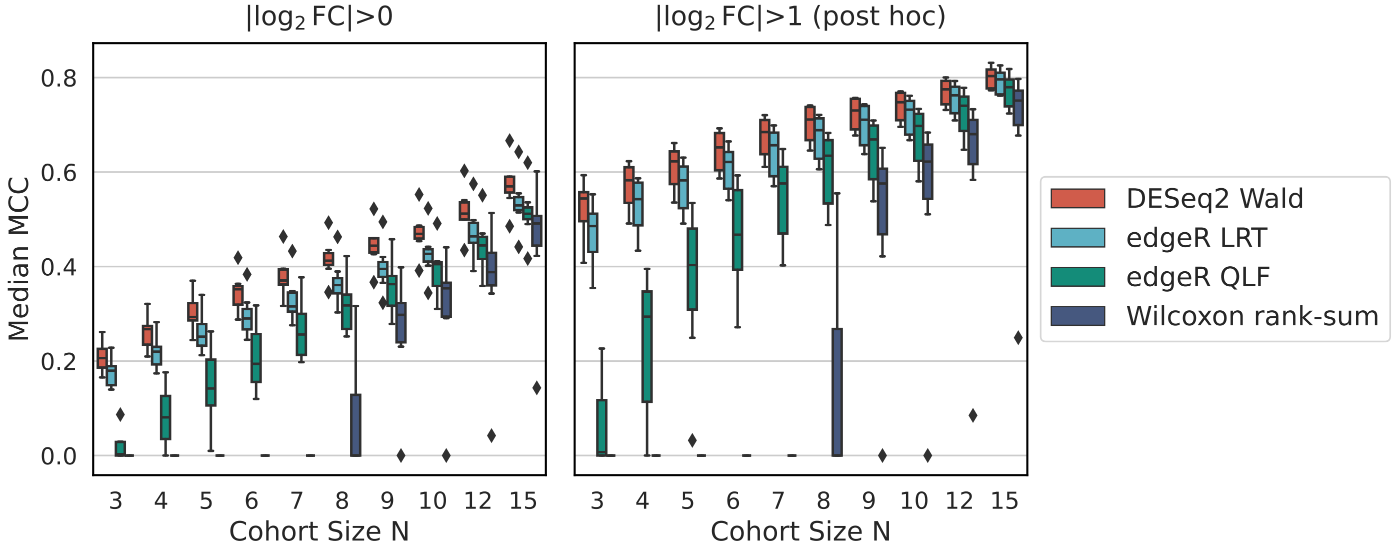

Figure F: MCC results with added Wilcoxon test. Each boxplot summarizes the eight normal–tumor tissue comparisons. MCC is set to 0 when it is undefined.

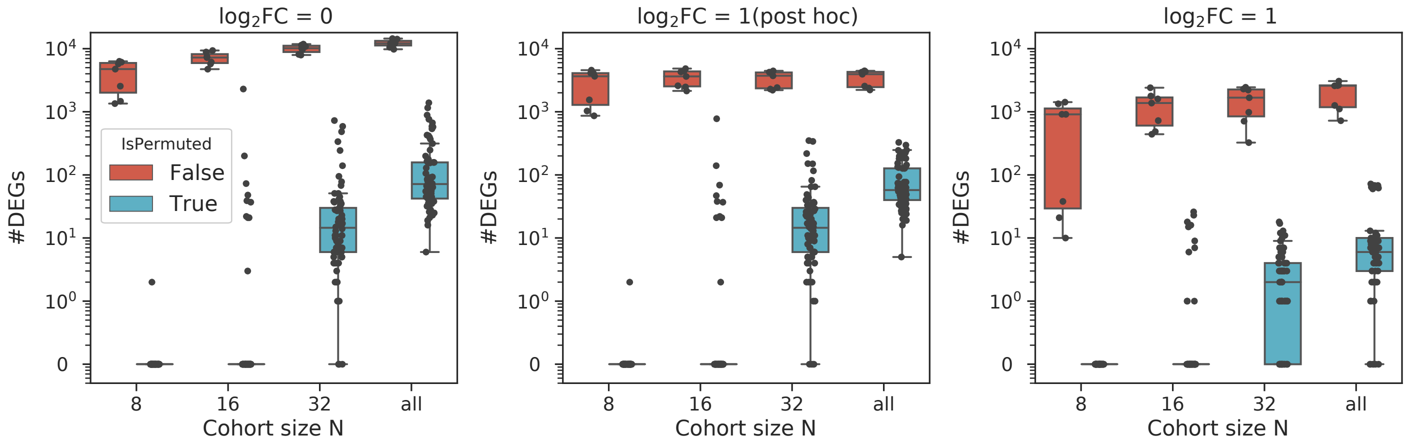

Figure G: DEGs from 8 permuted and unpermuted data sets using edgeR QLF with 5% FDR for different subsampled cohort sizes. We subsampled 15 trials of size N for each of the eight normal-tumor comparisons. The column *all* refers to unsubsampled cohorts (median $N = 52$).

3 Derivation of expected metrics under random gene selection

We present a derivation of the expected replicability of two experiments with randomly selected DEGs. Given two random subsets A and B of fixed sizes $0 < |A|, |B| \leq |N|$ drawn independently and uniformly without replacement from a parent set N, we first need to know the expected value of the intersection of A and B. This problem is equivalent to determining the number of successes k one can expect in n draws without replacement from a set of size $|N|$, where K of its elements are considered successes and the remaining elements are considered failures. Thus reformulated, the problem defines the well-known hypergeometric distribution with mean $E[k] = nK/|N|$. Without loss of generality, we set $|A| = n$ and $|B| = K$ to find

$$E[|A \cap B|] = \frac{|A||B|}{|N|}. \tag{2}$$

The expected value for the union of A and B is then

$$E[|A \cup B|] = E[|A| + |B| - |A \cap B|] = |A| + |B| - \frac{|A||B|}{|N|}. \tag{3}$$

Finally, the expected value for the Jaccard index (replicability) can be approximated by

$$E[Jacc(A, B)] = E[\frac{|A \cap B|}{|A \cup B|}] \approx \frac{E[|A \cap B|]}{E[|A \cup B|]} = \frac{|A||B|}{|N|(|A| + |B|) - |A||B|}, \tag{4}$$

which is equivalent to our approximation of R_0 in Equation 1. In the second line, we used a first-order Taylor expansion around the expected values of the numerator and denominator. Simulations show that the error of this approximation rapidly becomes negligible with increasing set sizes.

Similarly, expected values for the binary classification metrics (precision, recall) can be computed for the case of random gene selection. Consider a set of genes N, a non-empty subset of ground truth genes $G \subset N$, and a uniformly drawn non-empty subset of significant genes $S \subset N$. Let $s \in S$, then

$$E[\text{TP}] = |S| \times P(s \in G) = |S| \times \frac{|G|}{|N|}. \tag{5}$$

The expected values for FP, TN, FN can be obtained directly from $E[\text{TP}]$ and $|G|, |S|, |N|$. The metrics are then readily obtained as

$$\begin{aligned} E[\text{Precision}] &= \frac{|G|}{|N|}, \\ E[\text{Recall}] &= \frac{|S|}{|N|}. \end{aligned} \tag{6}$$

References

[1] Li Y, Ge X, Peng F, Li W, Li JJ. Exaggerated false positives by popular differential expression methods when analyzing human population samples. Genome Biology. 2022;23(1):79. doi:10.1186/s13059-022-02648-4.

[2] Chicco D, Jurman G. The advantages of the Matthews correlation coefficient (MCC) over F1 score and accuracy in binary classification evaluation. BMC Genomics. 2020;21(1):6. doi:10.1186/s12864-019-6413-7.
