# Supplementary material for: Replicability of bulk RNA-Seq differential expression and enrichment analysis results for small cohort sizes
Source: PLoS Comput Biol. 2025 May 5;21(5):e1011630. doi: 10.1371/journal.pcbi.1011630 (PMC12077797; doi:10.1371/journal.pcbi.1011630)
Supplement: S2 Text — Fig A: Ground truth size. Figs B–I: DEG performance metrics for additional tests and fold change thresholds. Fig J: KEGG enrichment performance metrics. Figs K–L: Bootstrapping results for enrichment analysis. Figs M–R: Predicting performance metrics from non-bootstrapped statistics. Fig S: Comparison of predictor statistics. Fig T: Variability of Spearman correlations. Figs U–W: Enrichment metrics for shrunken vs. unshrunken logFC. (PDF) [file pcbi.1011630.s002.pdf]

Replicability of bulk RNA-Seq differential expression and enrichment analysis results for small cohort sizes

Supporting Information 2

Peter Degen and Matúš Medo

April 15, 2025

Contents

1 Additional Figures **2**
1.1 Ground truth number of DEGs and enriched terms . . . 2
1.2 DEG performance metrics . . . 2
1.2.1 logFC 1 (formal) . . . 2
1.2.2 logFC 1 (post hoc) . . . 5
1.2.3 logFC 0 . . . 8
1.3 KEGG enrichment performance metrics . . . 11
1.4 Bootstrapping enrichment . . . 11
1.5 Non-bootstrapped statistics . . . 14
1.5.1 Predicting DEG performance metrics from non-bootstrapped statistics . . . 14
1.5.2 Predicting KEGG performance metrics from non-bootstrapped statistics . . . 15
1.5.3 Predicting GO performance metrics from non-bootstrapped statistics . . . 18
1.5.4 Comparison of predictor statistics . . . 19
1.6 Variability of Spearman correlation . . . 21
1.7 Enrichment metrics for shrunken vs. unshrunken logFC . . . 22

1 Additional Figures

1.1 Ground truth number of DEGs and enriched terms

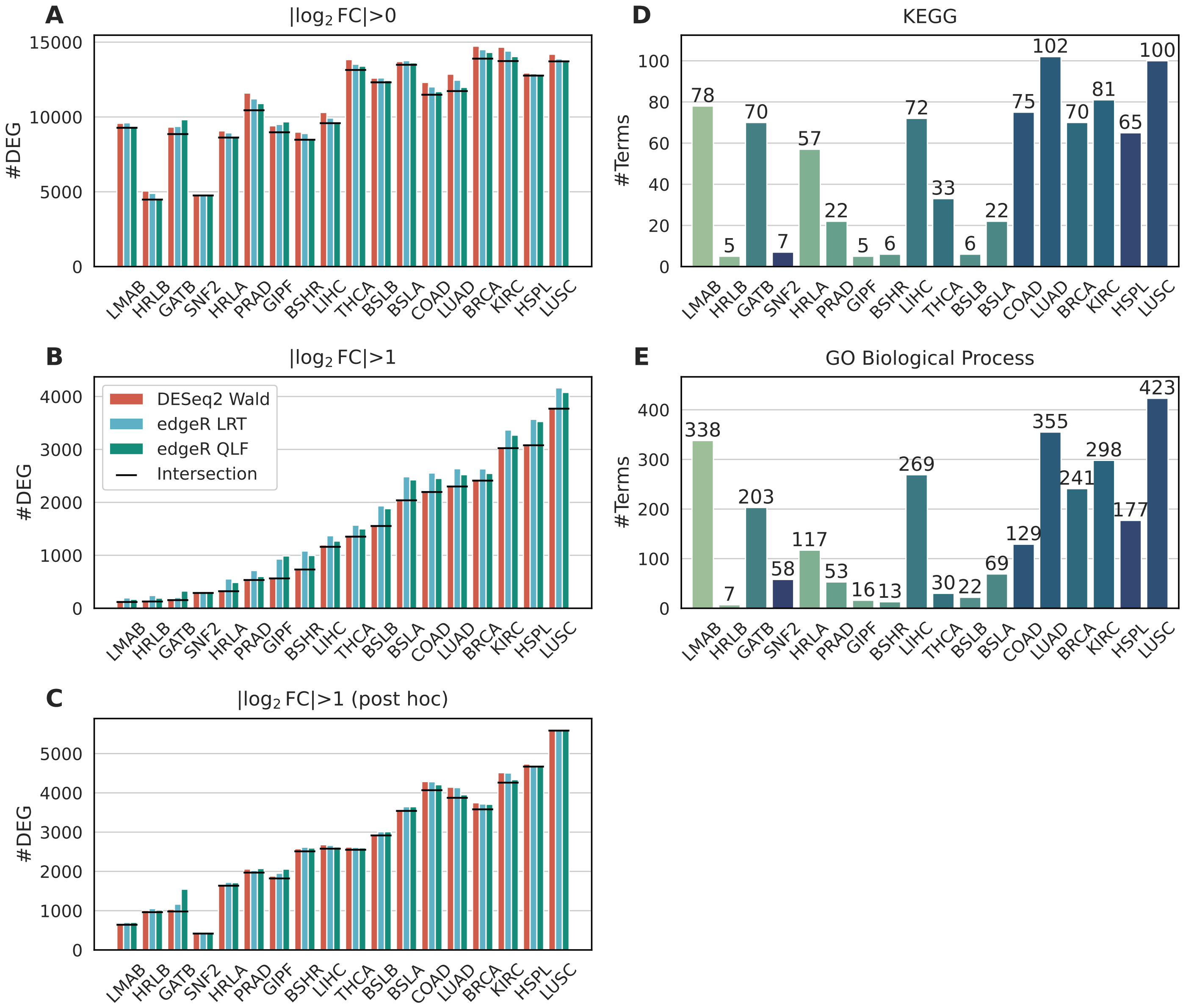

Figure A: **Ground truth size.** *Left column:* The number of ground truth DEGs for different data sets, differential expression methods, and fold-change thresholds. The number of DEGs found by different methods is similar and the intersection of DEGs is consistently high, indicating good agreement between the methods. Note the different ranges on the y-scale. *Right column:* The ground truth number of enriched terms for KEGG (D) and GO (E) libraries. In both cases, GSEA was performed using the shrunken logFC rankings estimated with DESeq2 from the full data sets. For all panels, significance is defined with a FDR threshold of 5%.

1.2 DEG performance metrics

1.2.1 logFC 1 (formal)

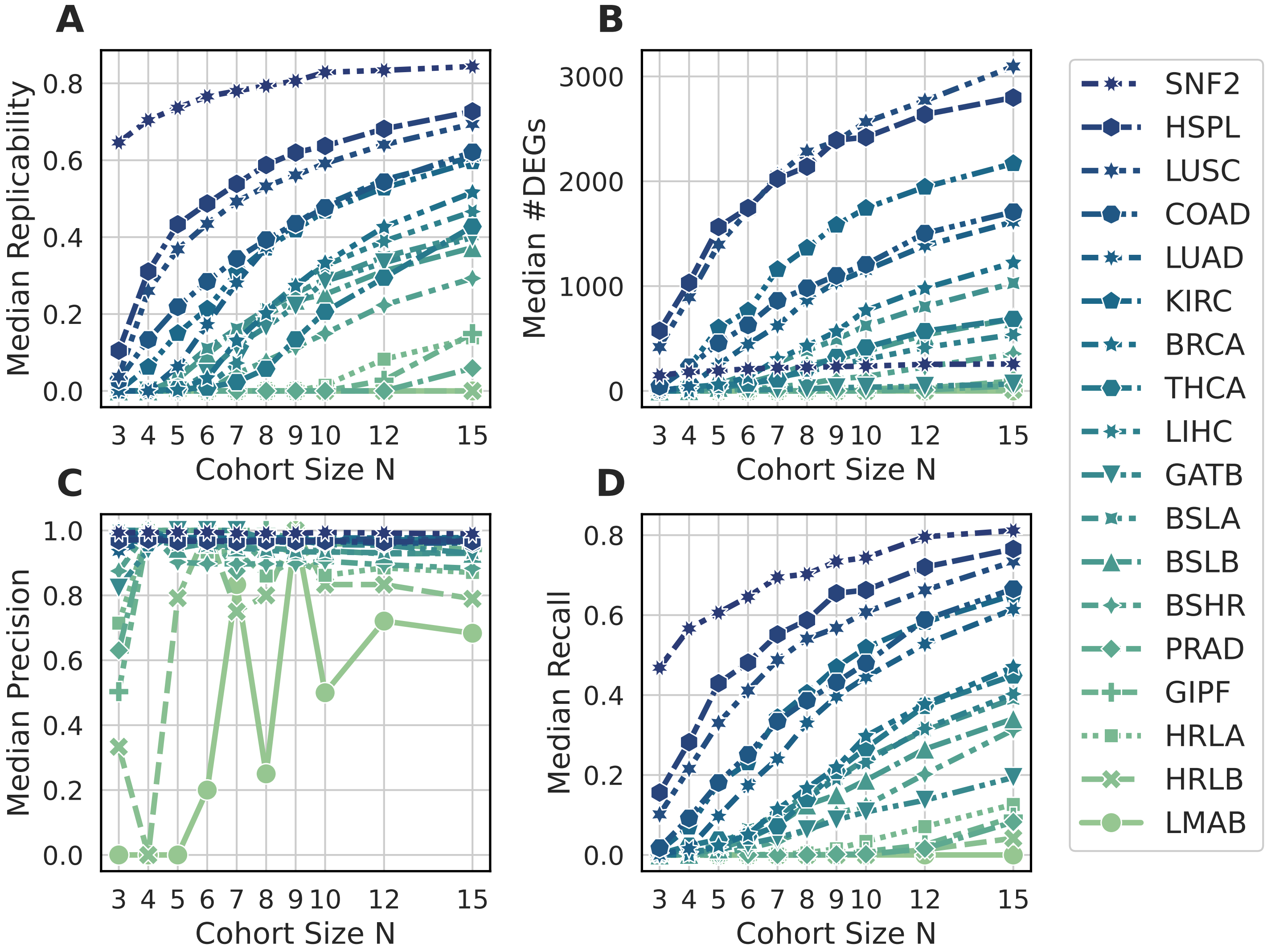

Figure B: **QLF DEG performance metrics as a function of the cohort size.** Each symbol summarizes the median of 100 cohorts. All panels show results using the edgeR QLF test with $|\log_2 \mathrm{FC}| > 1$.

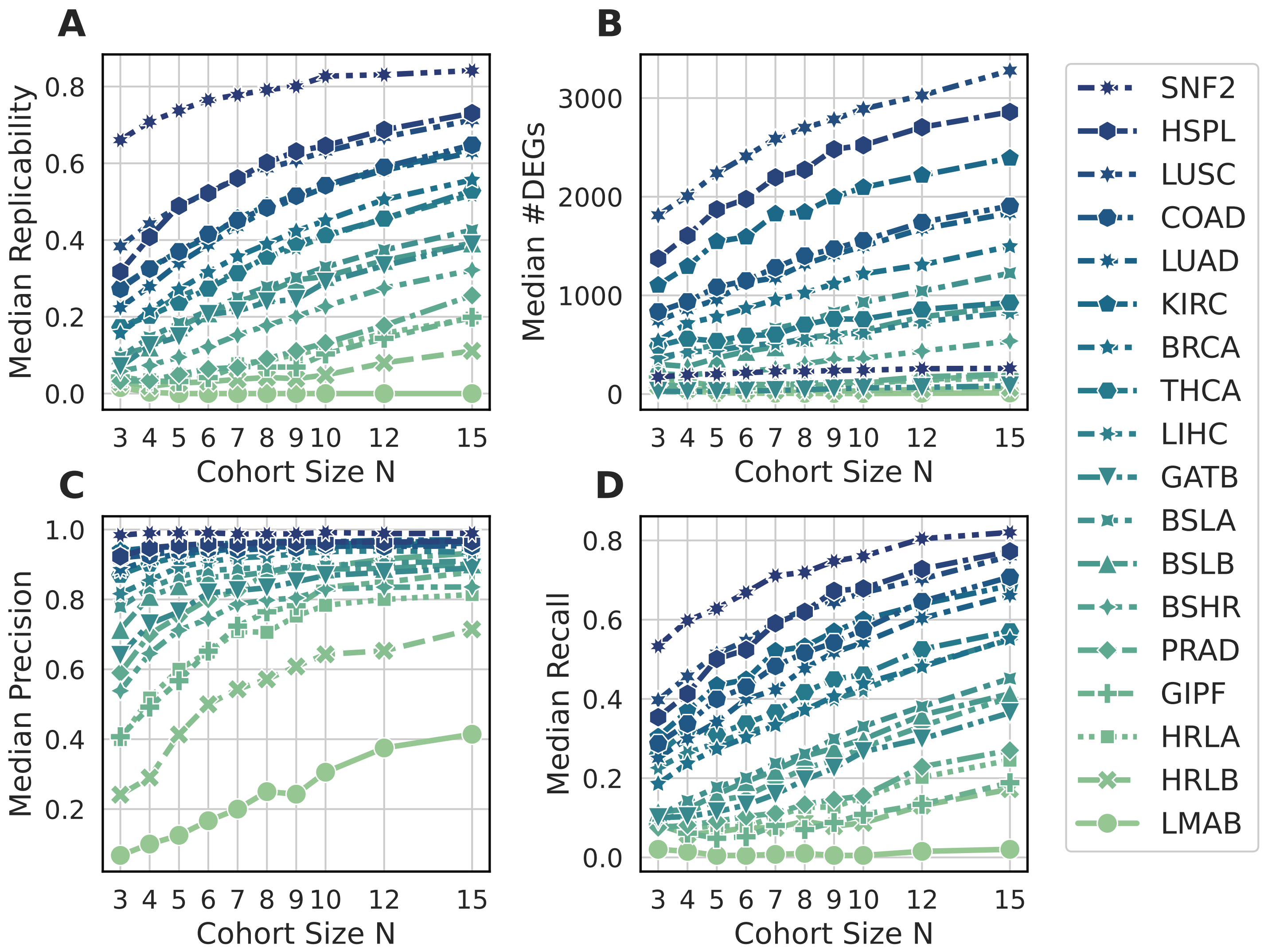

Figure C: **LRT DEG performance metrics as a function of the cohort size.** Each symbol summarizes the median of 100 cohorts. All panels show results using the edgeR LRT test with $|\log_2 \mathrm{FC}| > 1$.

1.2.2 logFC 1 (post hoc)

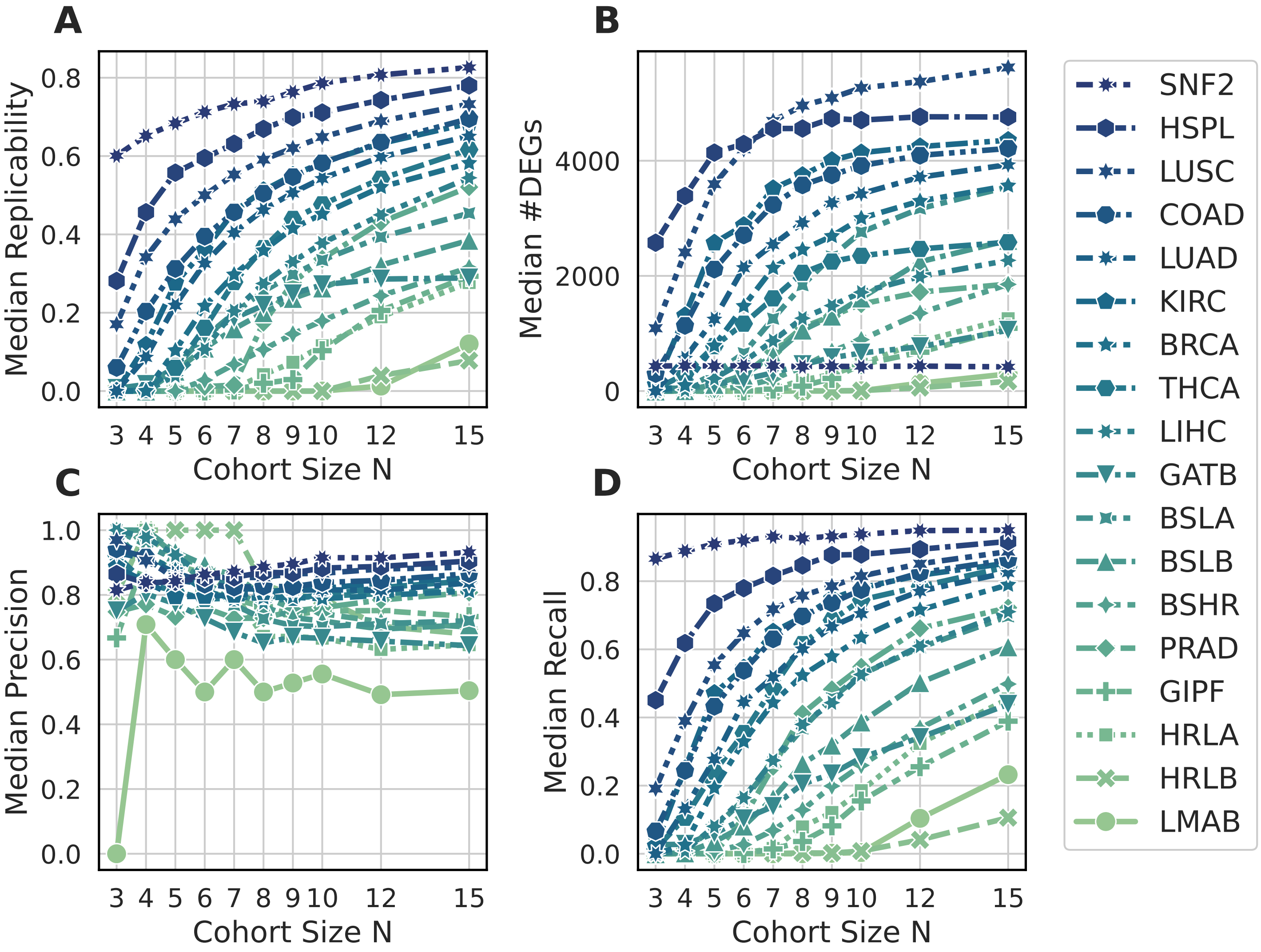

Figure D: **QLF DEG performance metrics as a function of the cohort size.** Each symbol summarizes the median of 100 cohorts. All panels show results using the edgeR QLF test with $|\log_2 \text{FC}| > 1$ (post hoc).

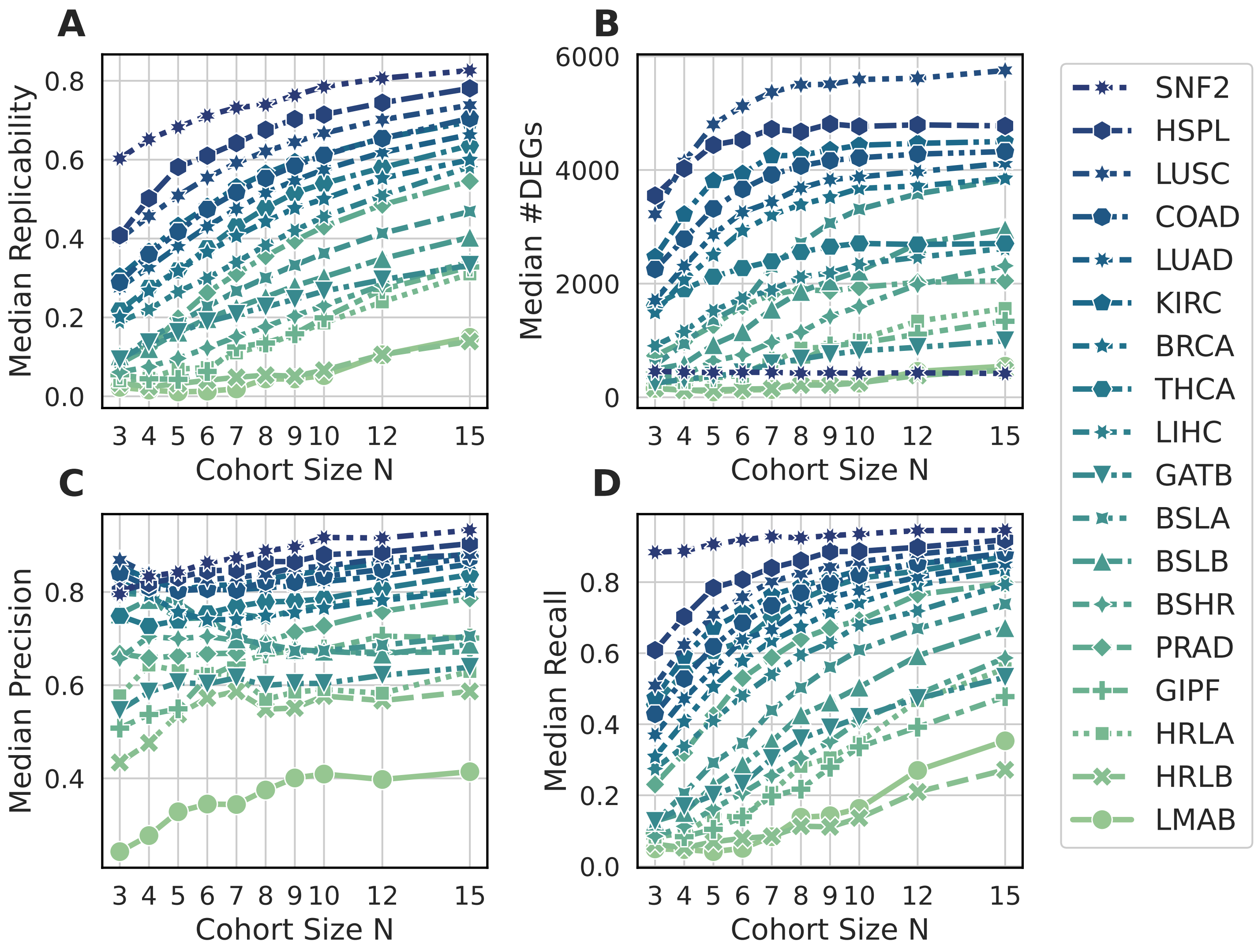

Figure E: **LRT DEG performance metrics as a function of the cohort size.** Each symbol summarizes the median of 100 cohorts. All panels show results using the edgeR LRT test with $|\log_2 \mathrm{FC}| > 1$ (post hoc).

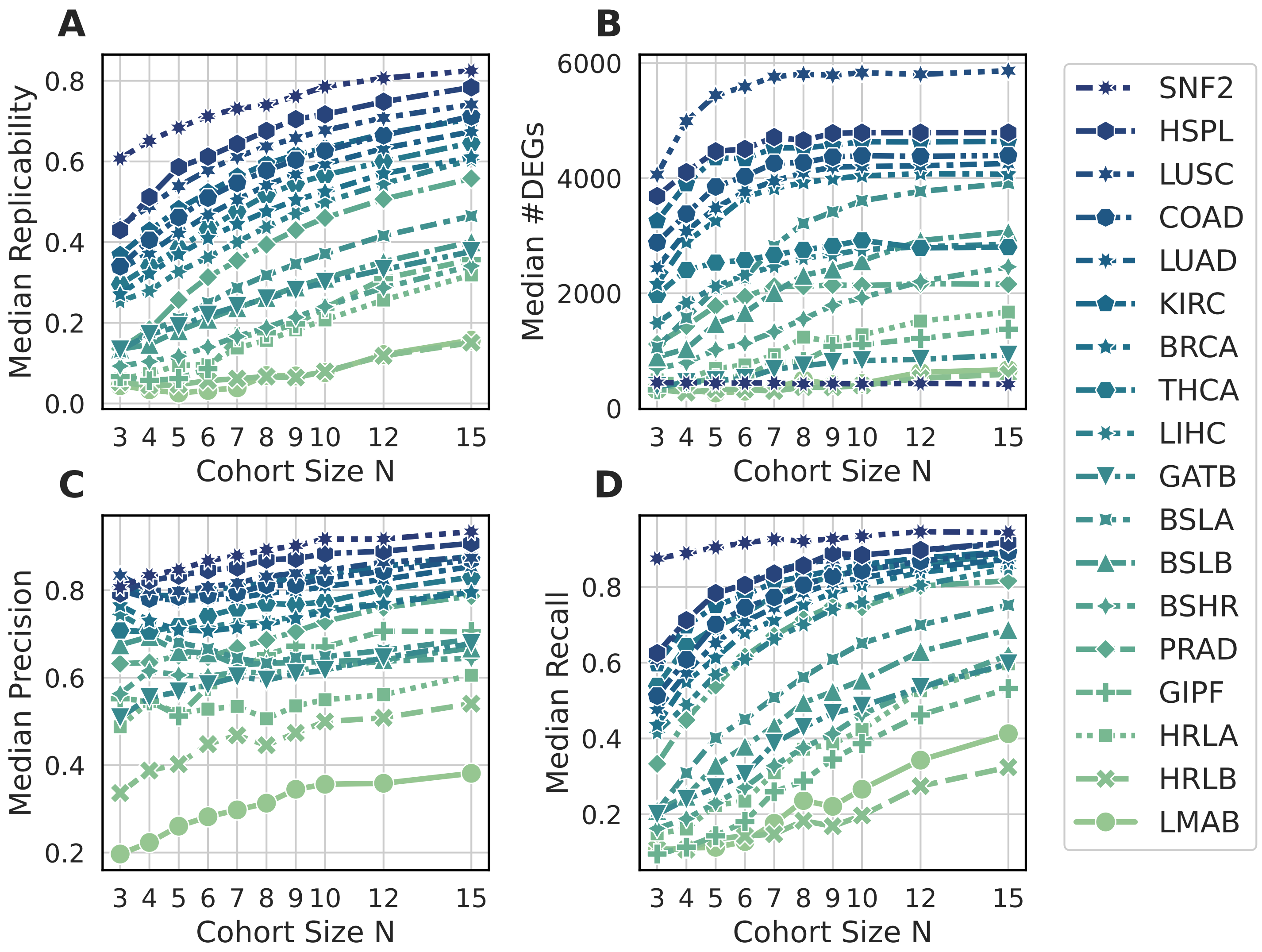

Figure F: **Wald DEG performance metrics as a function of the cohort size.** Each symbol summarizes the median of 100 cohorts. All panels show results using the DESeq2 Wald test with $|\log_2 \mathrm{FC}| > 1$ (post hoc).

1.2.3 logFC 0

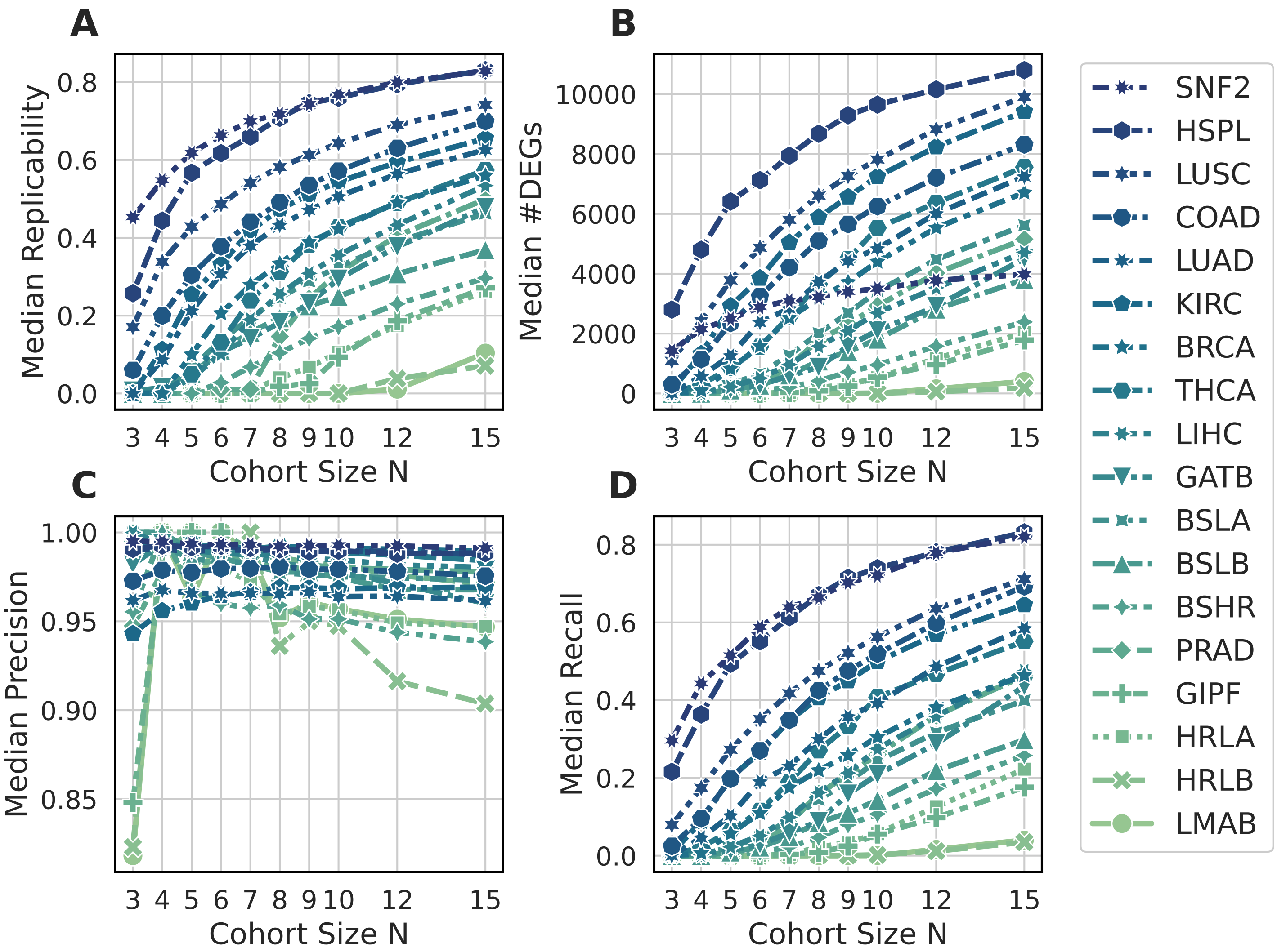

Figure G: **QLF DEG performance metrics as a function of the cohort size.** Each symbol summarizes the median of 100 cohorts. All panels show results using the edgeR QLF test with $|\log_2 \mathrm{FC}| > 0$.

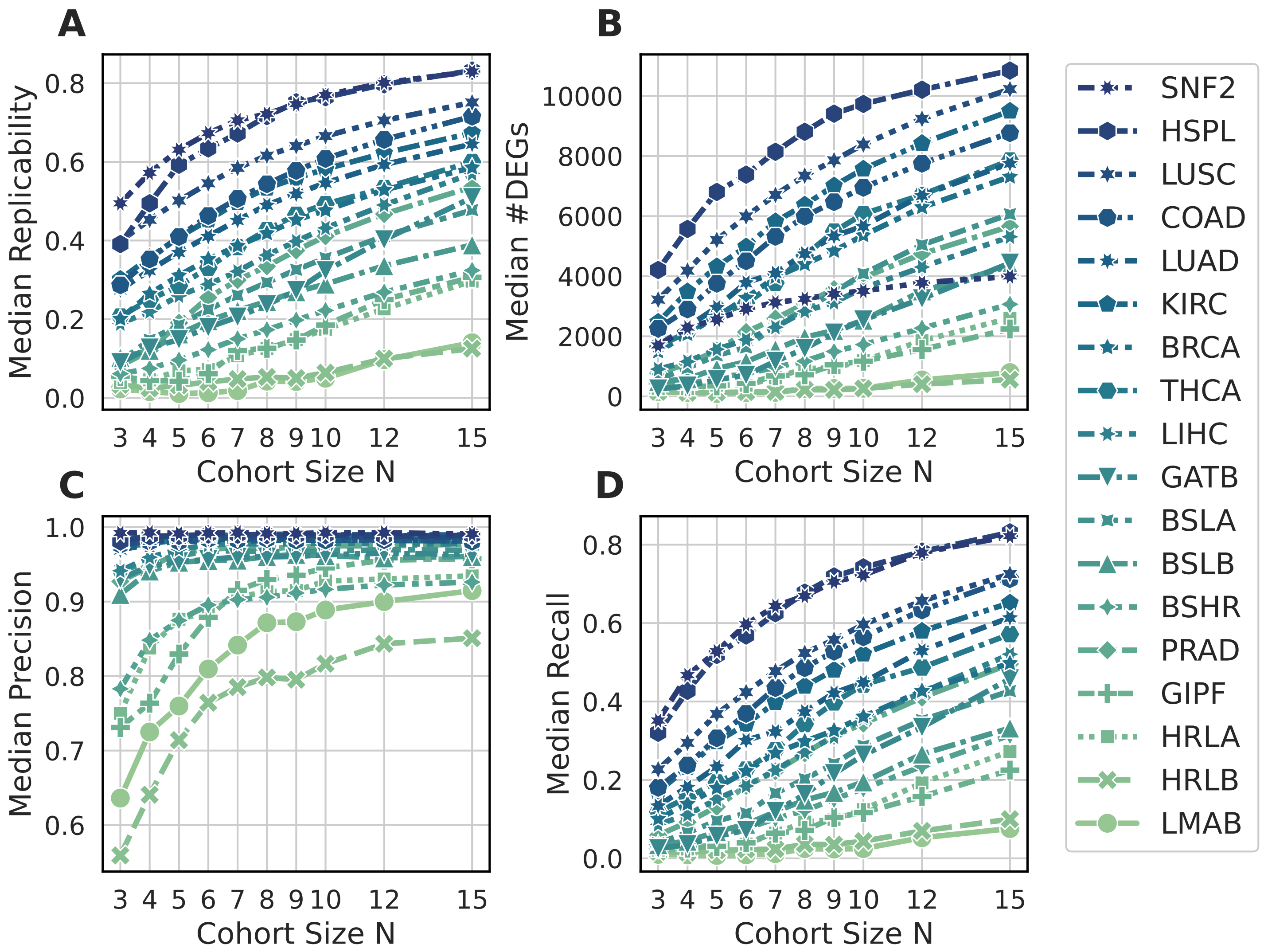

Figure H: **LRT DEG performance metrics as a function of the cohort size.** Each symbol summarizes the median of 100 cohorts. All panels show results using the edgeR LRT test with $|\log_2 \mathrm{FC}| > 0$.

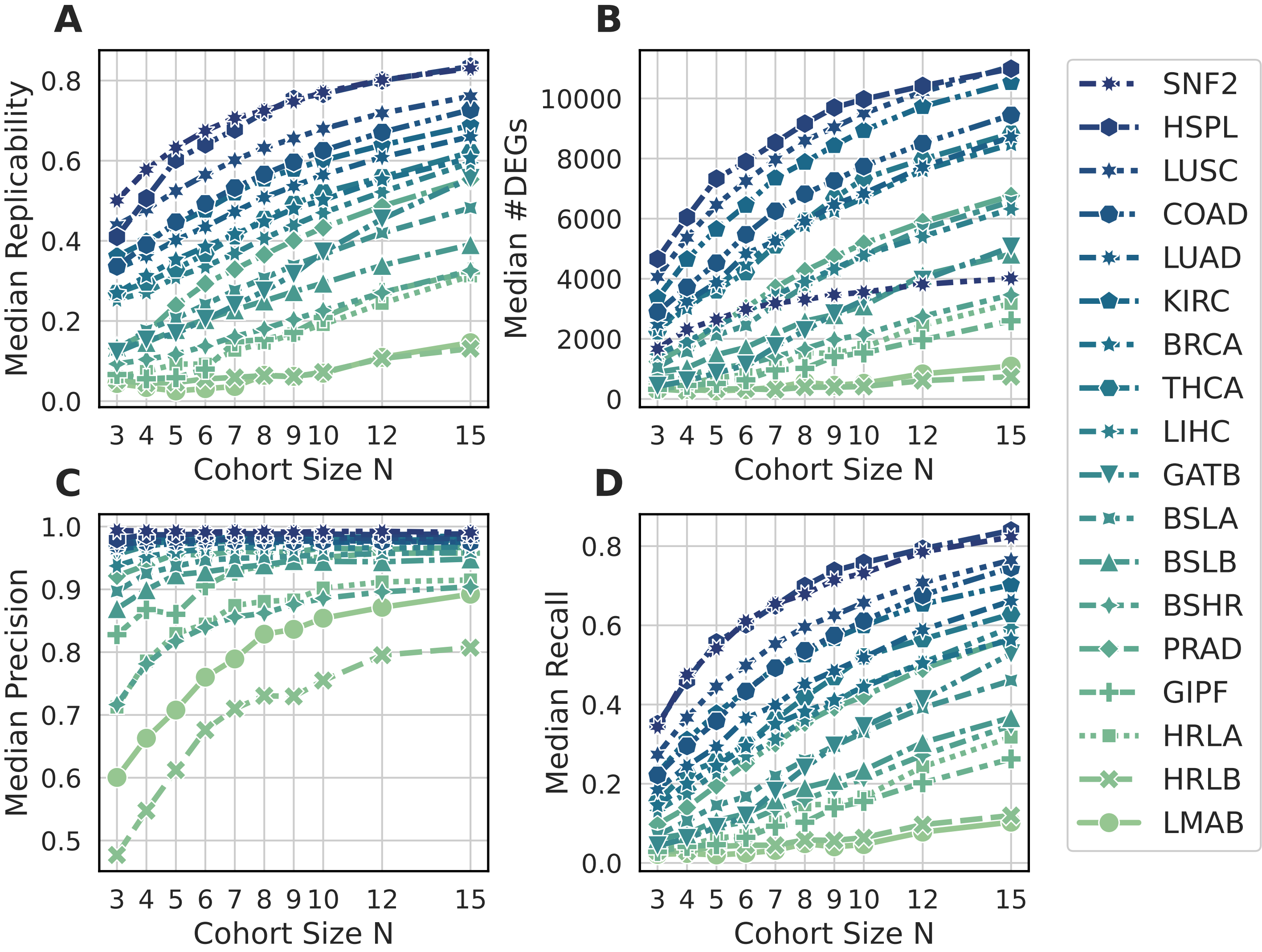

Figure I: **Wald DEG performance metrics as a function of the cohort size.** Each symbol summarizes the median of 100 cohorts. All panels show results using the DESeq2 Wald test with $|\log_2 \mathrm{FC}| > 0$.

1.3 KEGG enrichment performance metrics

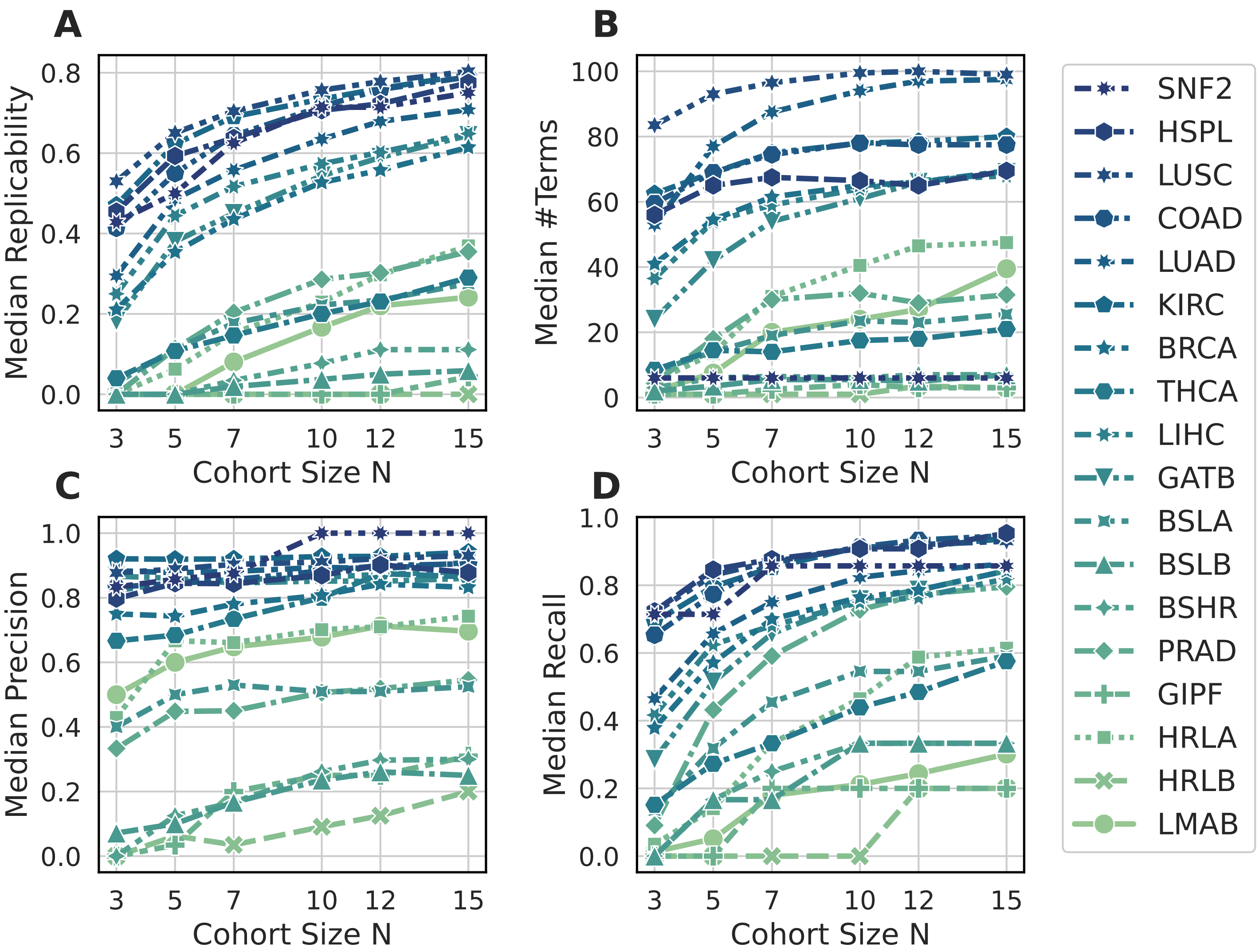

Figure J: **Enrichment performance metrics as a function of the cohort size.** Each symbol summarizes the median of 100 cohorts. All panels show enriched terms from KEGG. Results from GO BP are shown in the main text.

1.4 Bootstrapping enrichment

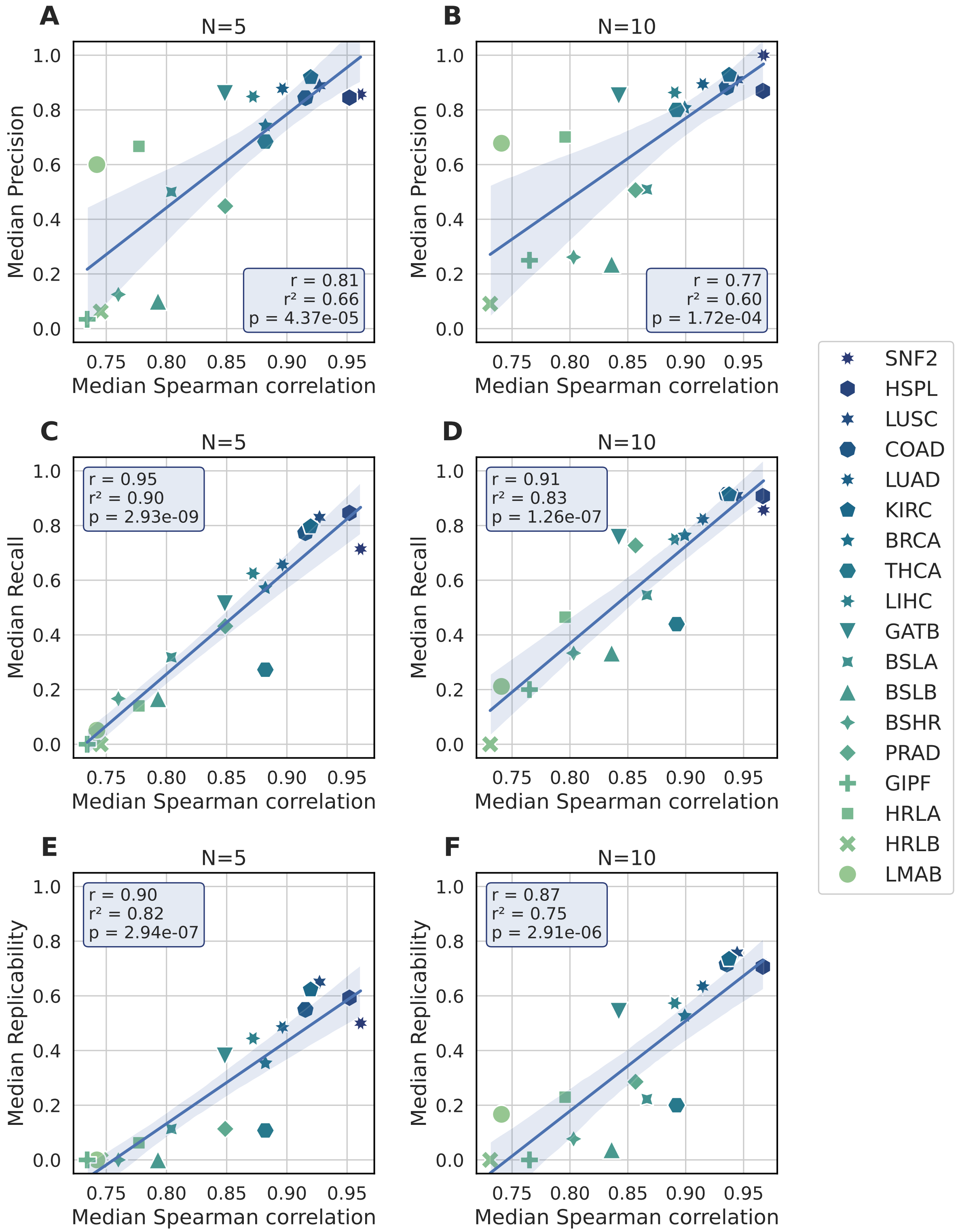

Figure K: **Bootstrapping results.** Performance metrics (precision, recall, and replicability) of significant KEGG pathways versus Spearman rank correlation of log fold change estimates for cohort sizes $N \in \{5, 10\}$.

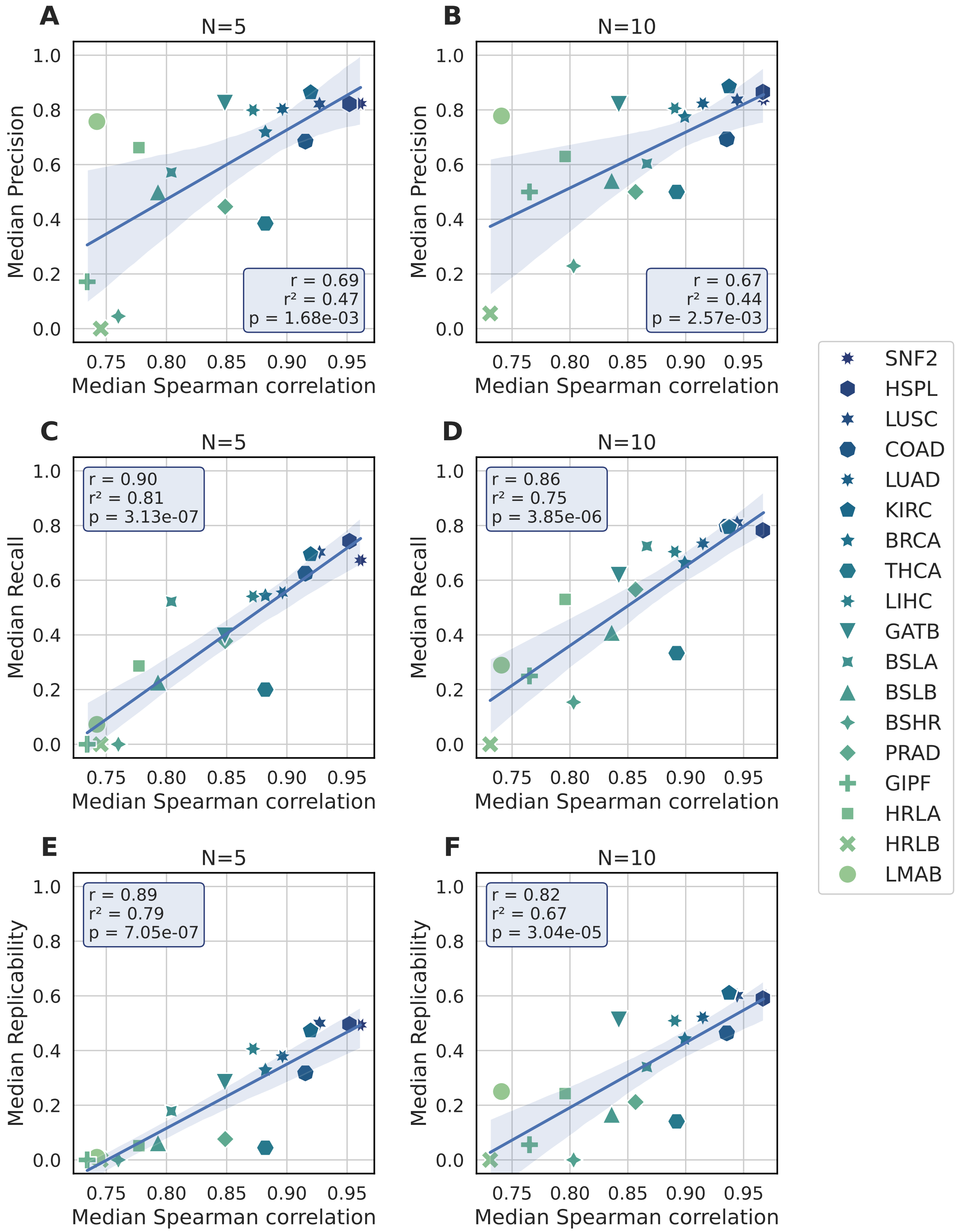

Figure L: **Bootstrapping results.** Performance metrics (precision, recall, and replicability) of significant GO BP terms versus Spearman rank correlation of log fold change estimates for cohort sizes $N \in \{5, 10\}$.

1.5 Non-bootstrapped statistics

1.5.1 Predicting DEG performance metrics from non-bootstrapped statistics

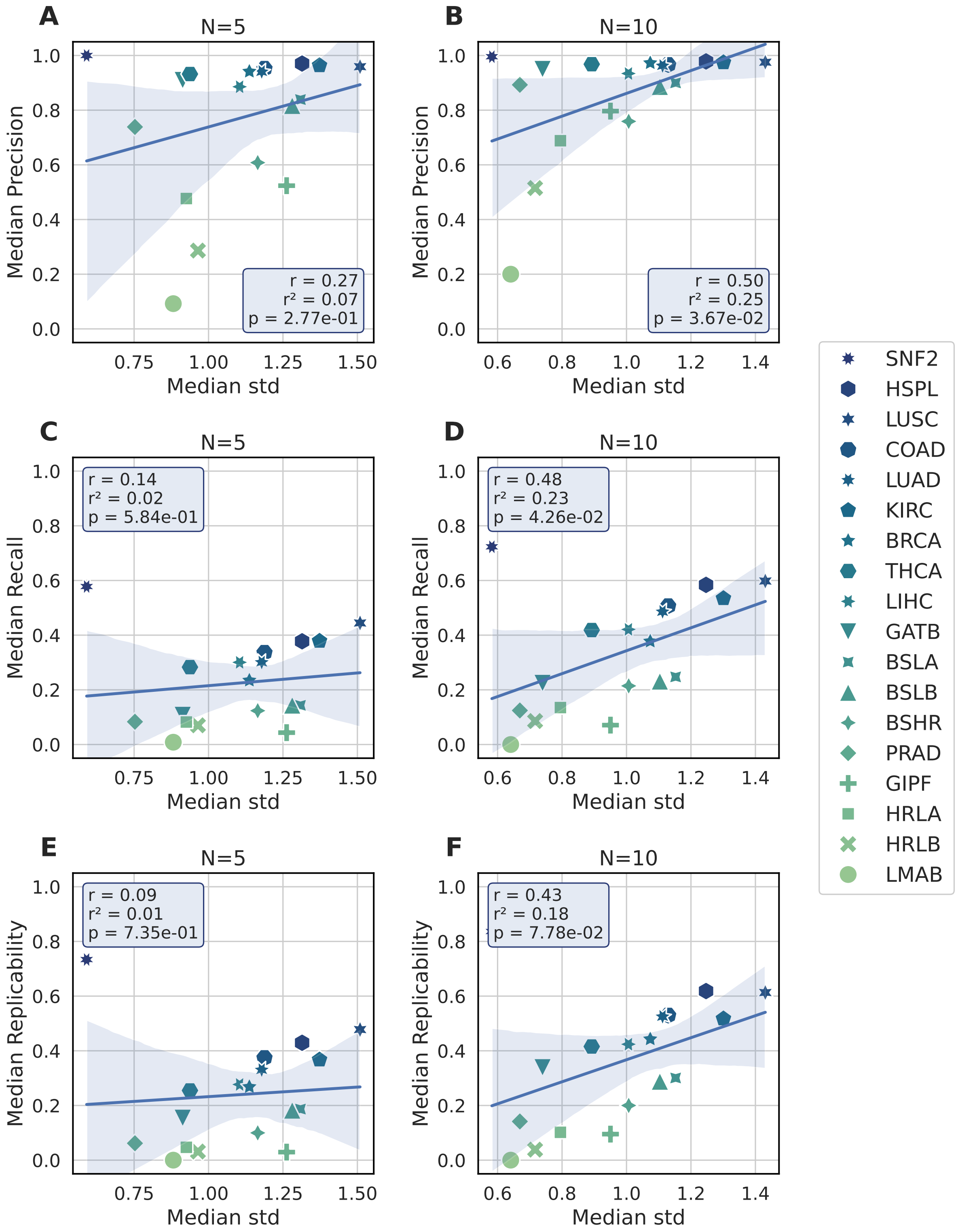

Figure M: **Predicting DEG performance metrics from the logFC standard deviation.** Analogous to Fig 5 in the main text, but using a non-bootstrapped statistic on the x-axis.

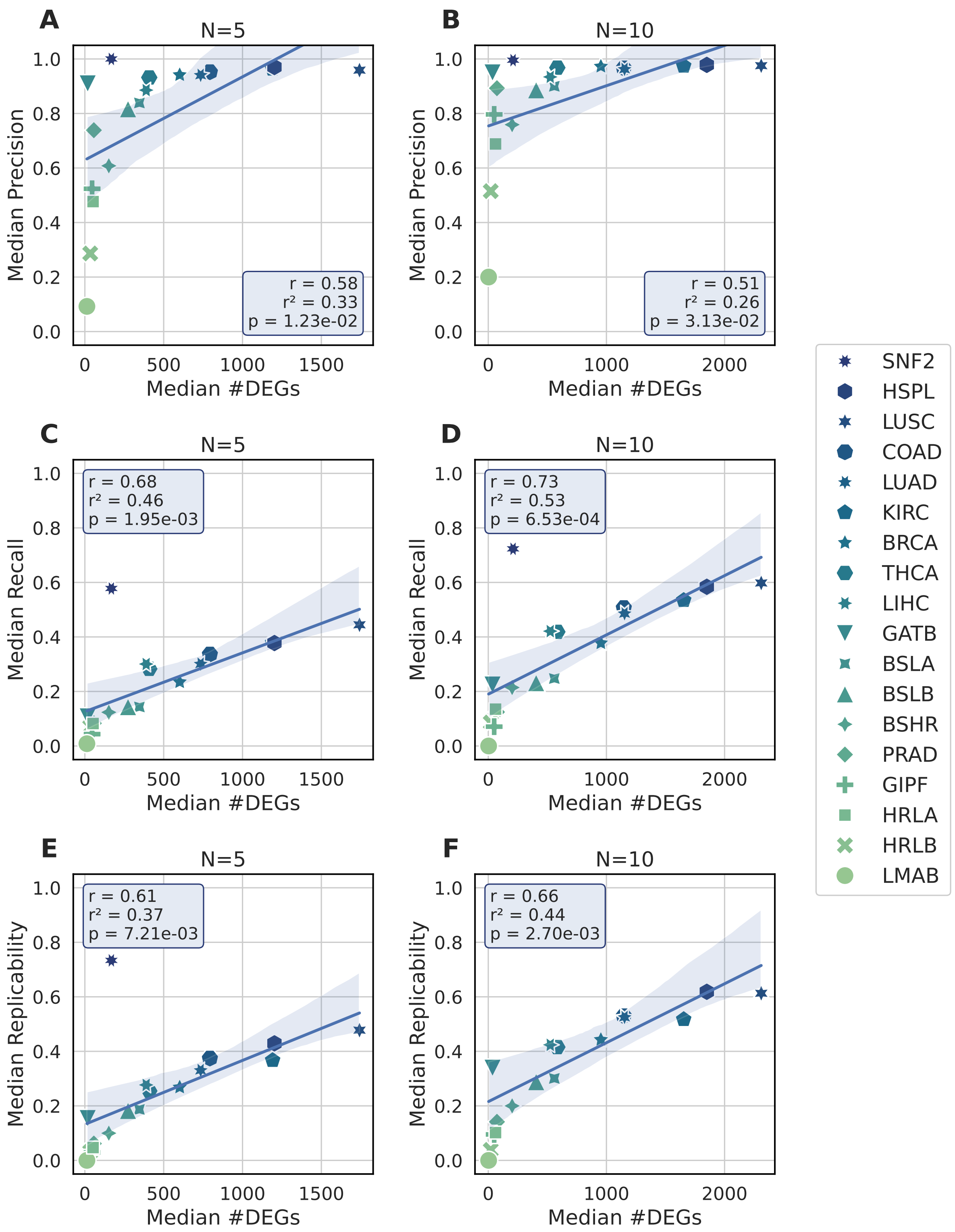

Figure N: **Predicting DEG performance metrics from the number of DEGs.** Analogous to Fig 5 in the main text, but using a non-bootstrapped statistic on the x-axis.

1.5.2 Predicting KEGG performance metrics from non-bootstrapped statistics

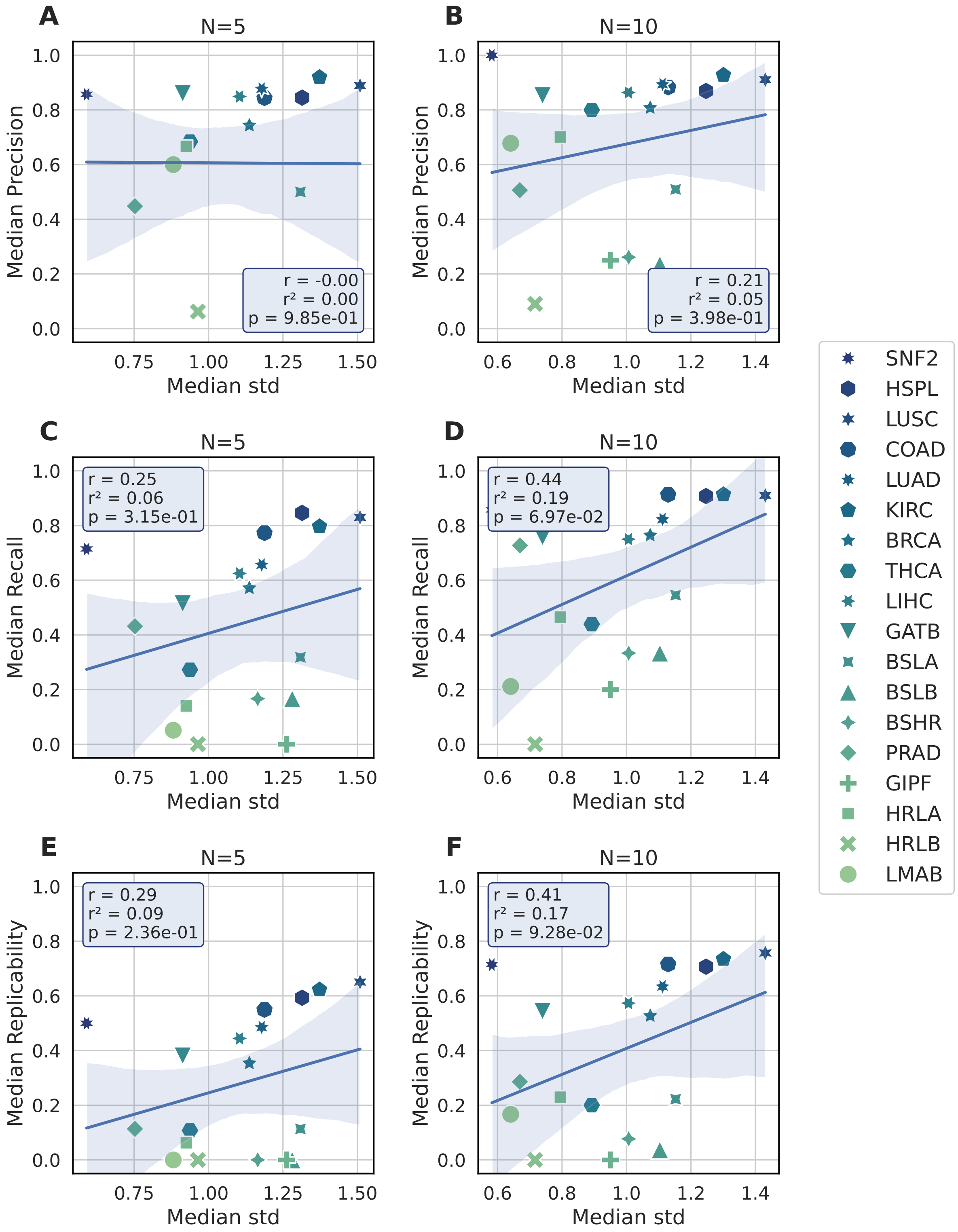

Figure O: **Predicting KEGG performance metrics from the logFC standard deviation.** Analogous to Fig K in this document, but using a non-bootstrapped statistic on the x-axis.

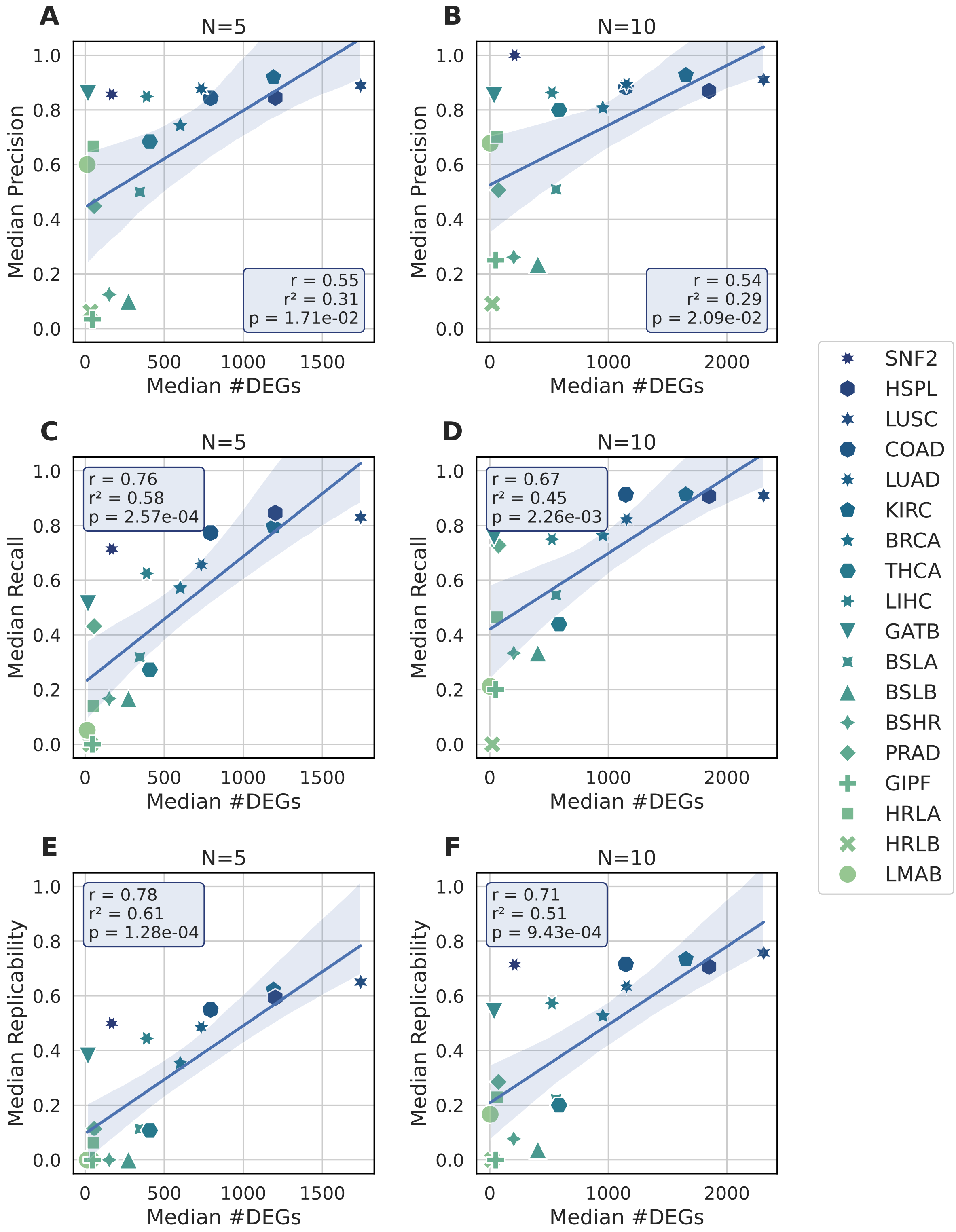

Figure P: **Predicting KEGG performance metrics from the number of DEGs.** Analogous to Fig K in this document, but using a non-bootstrapped statistic on the x-axis.

1.5.3 Predicting GO performance metrics from non-bootstrapped statistics

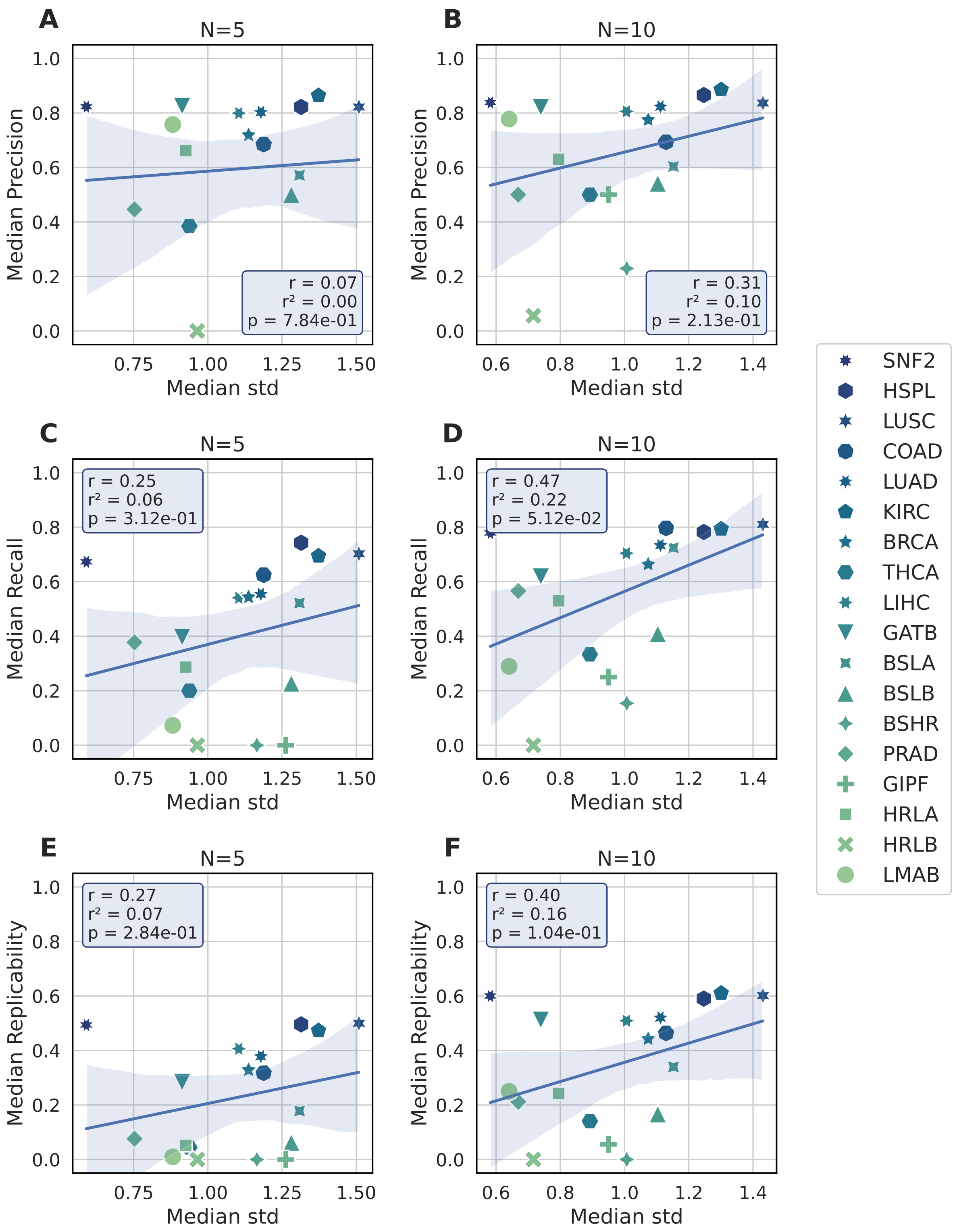

Figure Q: **Predicting GO performance metrics from the logFC standard deviation.** Analogous to Fig L in this document, but using a non-bootstrapped statistic on the x-axis.

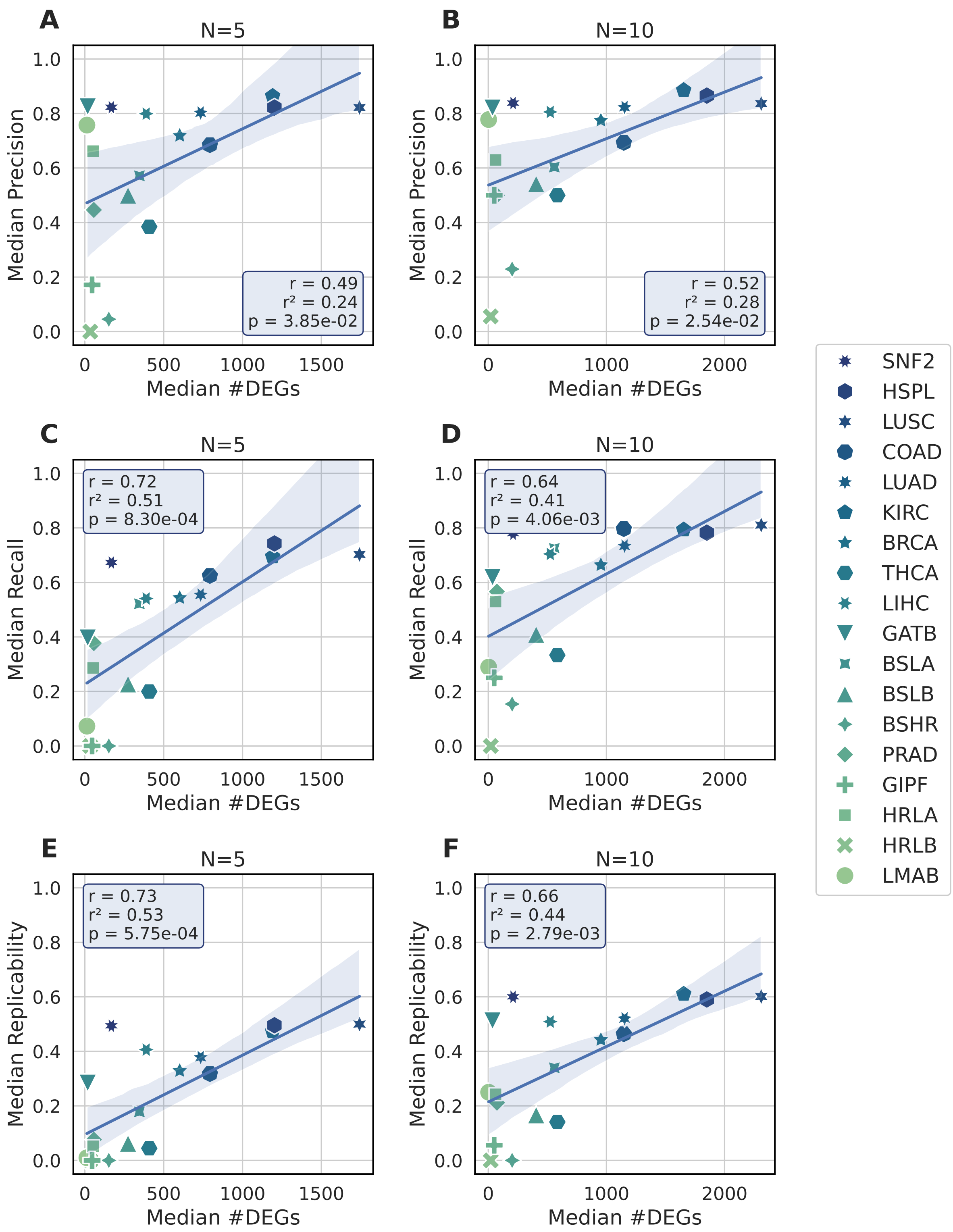

Figure R: **Predicting GO performance metrics from the number of DEGs.** Analogous to Fig L in this document, but using a non-bootstrapped statistic on the x-axis.

1.5.4 Comparison of predictor statistics

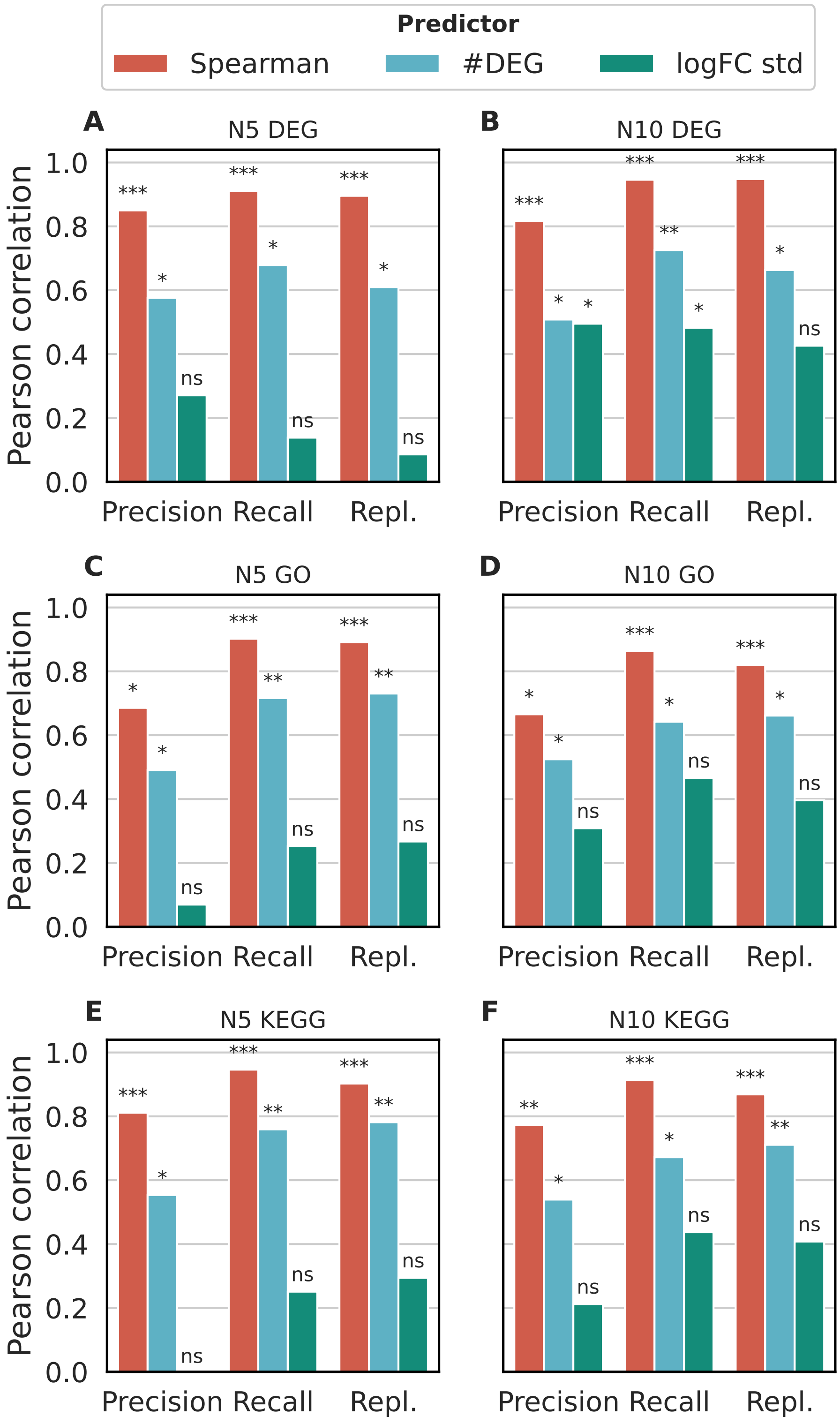

Figure S: **Comparison of predictor statistics**. The bootstrapped Spearman rank correlation is compared with two non-bootstrapped statistics: the number of DEGs detected and the standard deviation of the log fold change distribution of the original cohort. Pearson correlations and p-values are as in Fig 5 from the main text and Fig M–R from this document. A high Pearson correlation indicates that the predictor is strongly correlated with the respective performance metric (precision, recall, replicability). *=$p< 0.05$; **=$p< 0.001$; ***=$p< 0.0001$.

1.6 Variability of Spearman correlation

From the data points in Fig TB and TD, we can calculate the empirical probability that the precision exceeds one of our heuristic thresholds (low: 0.8, high: 0.9), conditioned on the Spearman correlation exceeding a given threshold and the cohort size N:

$$
\begin{aligned}
P(Precision > 0.9 \mid Spearman > 0.9 \wedge N = 5) &= \frac{240}{284} = 0.85 \\
P(Precision < 0.8 \mid Spearman > 0.9 \wedge N = 5) &= \frac{15}{284} = 0.05 \\
P(Precision < 0.8 \mid Spearman < 0.8 \wedge N = 5) &= \frac{217}{276} = 0.79 \\
P(Precision > 0.9 \mid Spearman < 0.8 \wedge N = 5) &= \frac{12}{276} = 0.04 \\
P(Precision > 0.9 \mid Spearman > 0.9 \wedge N = 10) &= \frac{340}{355} = 0.96 \\
P(Precision < 0.8 \mid Spearman > 0.9 \wedge N = 10) &= \frac{4}{355} = 0.01 \\
P(Precision < 0.8 \mid Spearman < 0.8 \wedge N = 10) &= \frac{146}{206} = 0.71 \\
P(Precision > 0.9 \mid Spearman < 0.8 \wedge N = 10) &= \frac{21}{206} = 0.10
\end{aligned} \tag{1}
$$

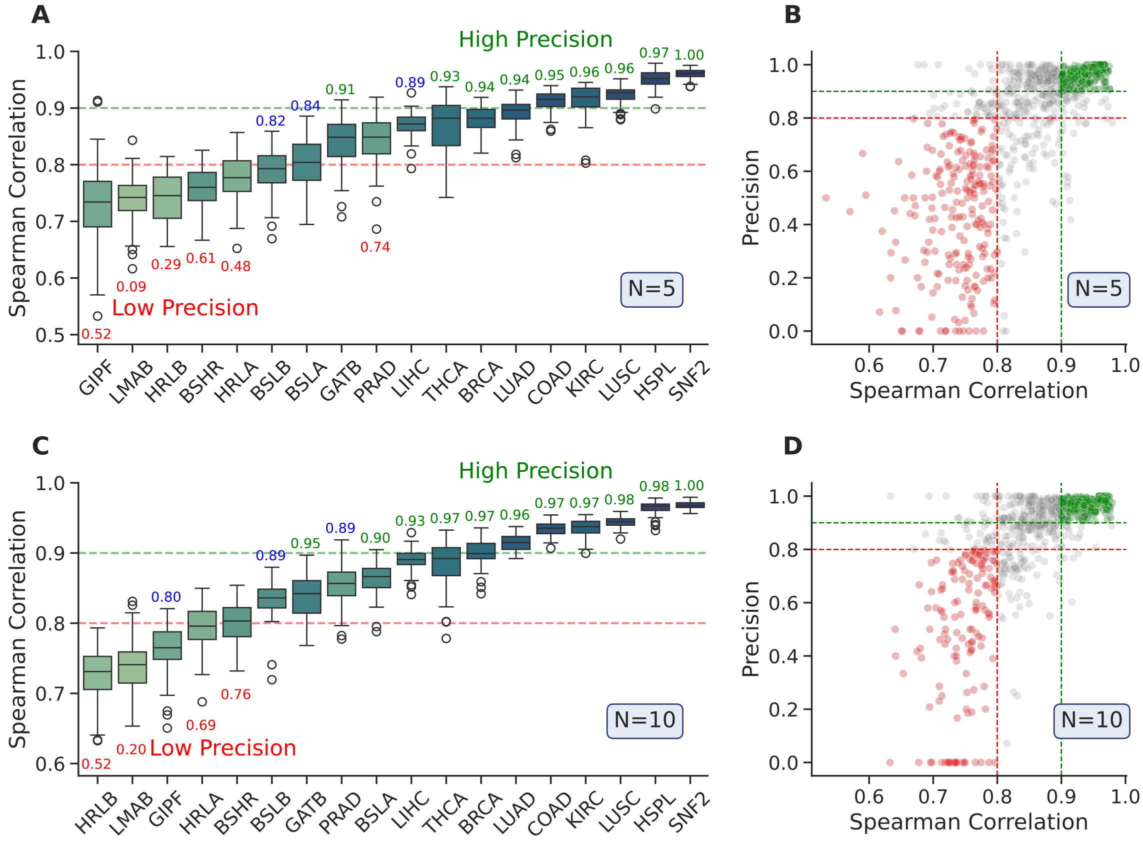

Figure T: **Variability of Spearman correlations.** *Left column:* Box plots summarizing data points from 50 cohorts of size $N \in \{5, 10\}$, each of which represents the mean Spearman correlation in 25 bootstrap trials. Also shown is the median precision of DEGs for the respective data set printed directly on top or below the box plots. We define two performance regimes using thresholds of > 0.9 (high precision, high Spearman) and < 0.8 (low precision, low Spearman). *Right column:* Spearman correlations and precision metrics for 50 cohorts $\times$ 18 data sets $= 900$ cohorts for each $N \in \{5, 10\}$. Shaded in red are data points for which Spearman and precision are below 0.8. Shaded in green are cohorts for which Spearman and precision are above 0.9.

1.7 Enrichment metrics for shrunken vs. unshrunken logFC

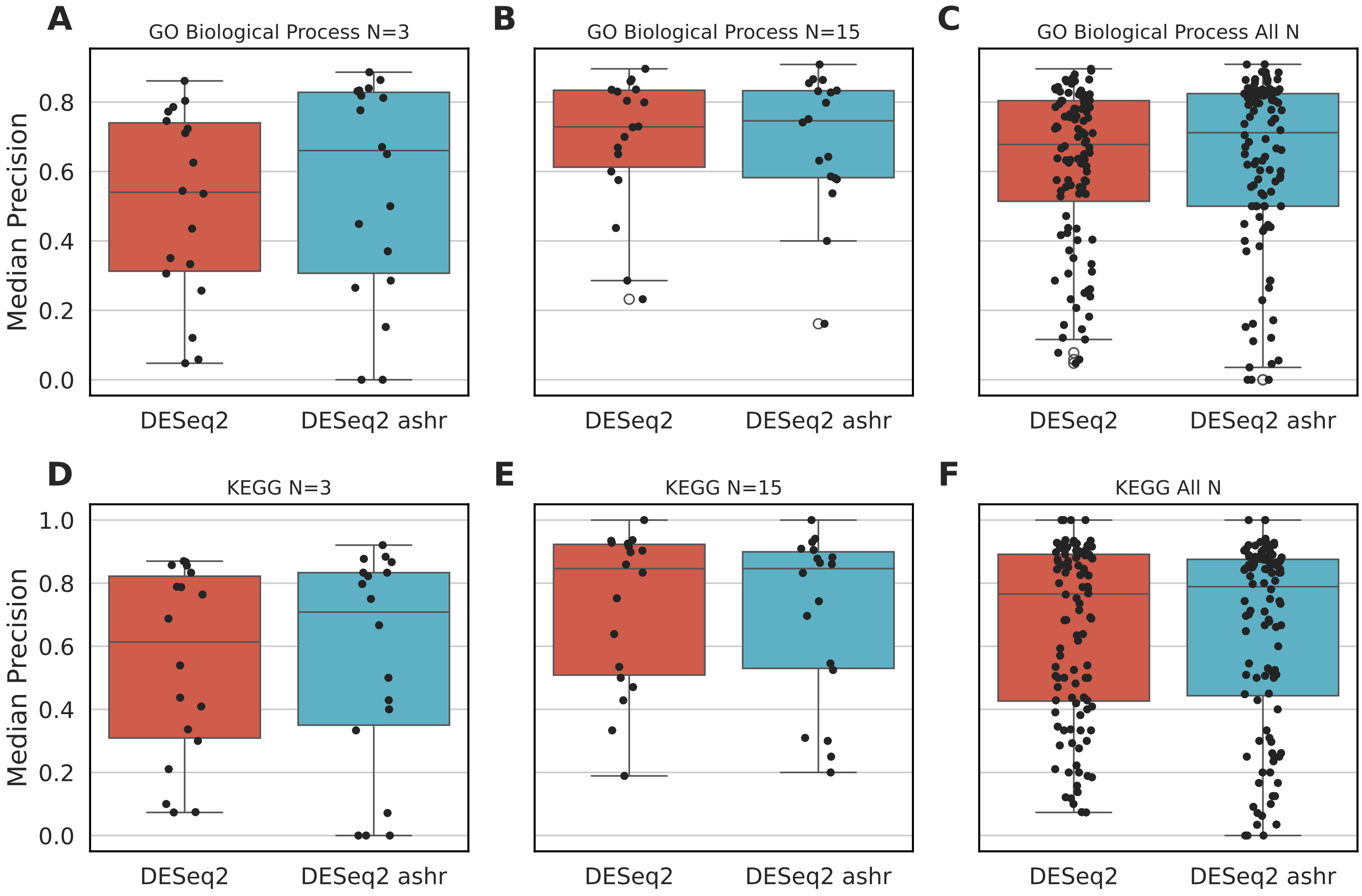

Figure U: **Enrichment precision.** *Left column:* Comparison of precision of enriched terms obtained from gene rankings with and without logFC shrinkage. Each black dot represents the median of 100 cohorts of size $N = 3$ for one of the 18 data sets. Red box plots show results obtained from unshrunken logFC rankings with DESeq2. Blue box plots show results obtained from logFC estimates with adaptive shrinkage (ashr). *Middle column:* As the left column, but for $N = 15$. *Right colum:* As the previous columns, but for all cohort sizes $N \in \{3, 5, 7, 10, 12, 15\}$.

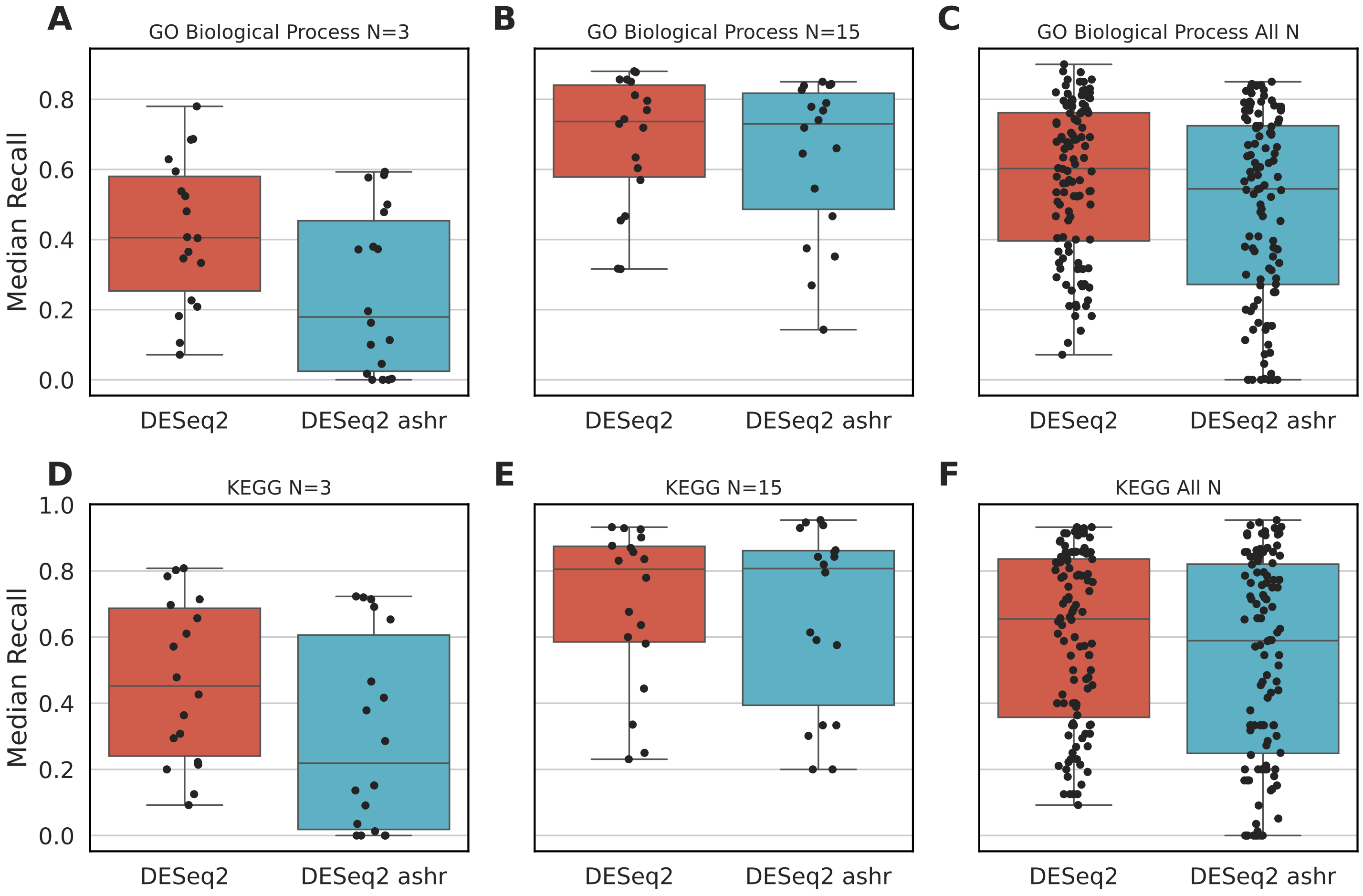

Figure V: **Enrichment recall.** *Left column:* Comparison of recall of enriched terms obtained from gene rankings with and without logFC shrinkage. Each black dot represents the median of 100 cohorts of size $N = 3$ for one of the 18 data sets. Red box plots show results obtained from unshrunken logFC rankings with DESeq2. Blue box plots show results obtained from logFC estimates with adaptive shrinkage (ashr). *Middle column:* As the left column, but for $N = 15$. *Right colum:* As the previous columns, but for all cohort sizes $N \in \{3, 5, 7, 10, 12, 15\}$.

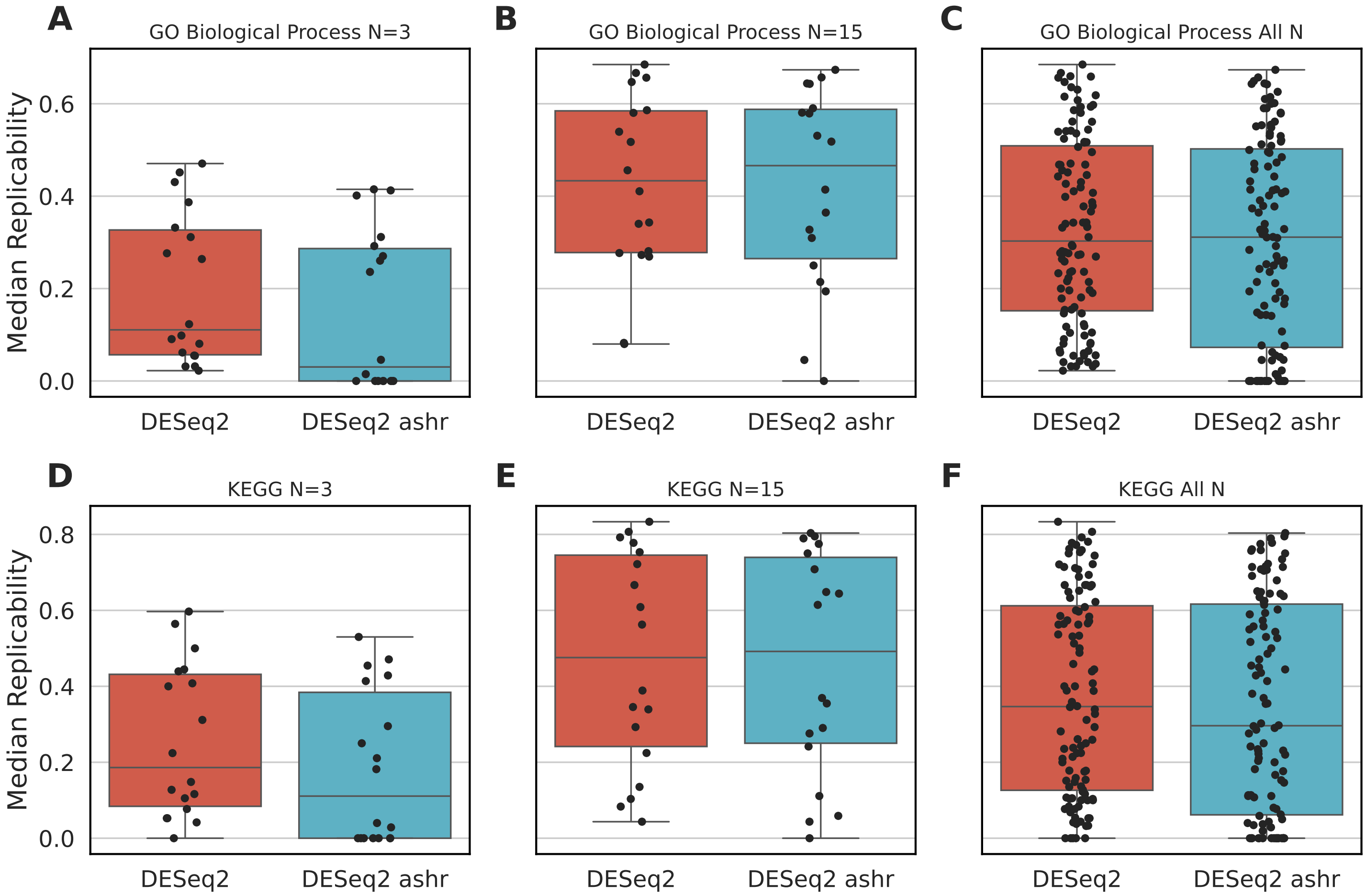

Figure W: **Enrichment replicability.** *Left column:* Comparison of replicability of enriched terms obtained from gene rankings with and without logFC shrinkage. Each black dot represents the median of 100 cohorts of size $N = 3$ for one of the 18 data sets. Red box plots show results obtained from unshrunken logFC rankings with DESeq2. Blue box plots show results obtained from logFC estimates with adaptive shrinkage (ashr). *Middle column:* As the left column, but for $N = 15$. *Right colum:* As the previous columns, but for all cohort sizes $N \in \{3, 5, 7, 10, 12, 15\}$.
