# Supplementary material for: Replicability of bulk RNA-Seq differential expression and enrichment analysis results for small cohort sizes
Source: PLoS Comput Biol. 2025 May 5;21(5):e1011630. doi: 10.1371/journal.pcbi.1011630 (PMC12077797; doi:10.1371/journal.pcbi.1011630)
Supplement: S3 Text — Fig A–H: Heat maps for the remaining data sets (not including SNF2 and LMAB). Fig I–P: Fold change figures for the remaining data sets. (PDF) [file pcbi.1011630.s003.pdf]

Replicability of bulk RNA-Seq differential expression and enrichment analysis results for small cohort sizes

Supporting Information 3

Peter Degen and Matúš Medo

April 15, 2025

Contents

1 Additional Figures **2**
1.1 Heat maps for the remaining data sets 2
1.2 Fold change figures for the remaining data sets 6

1 Additional Figures

1.1 Heat maps for the remaining data sets

See main text for SNF2 and LMAB data sets.

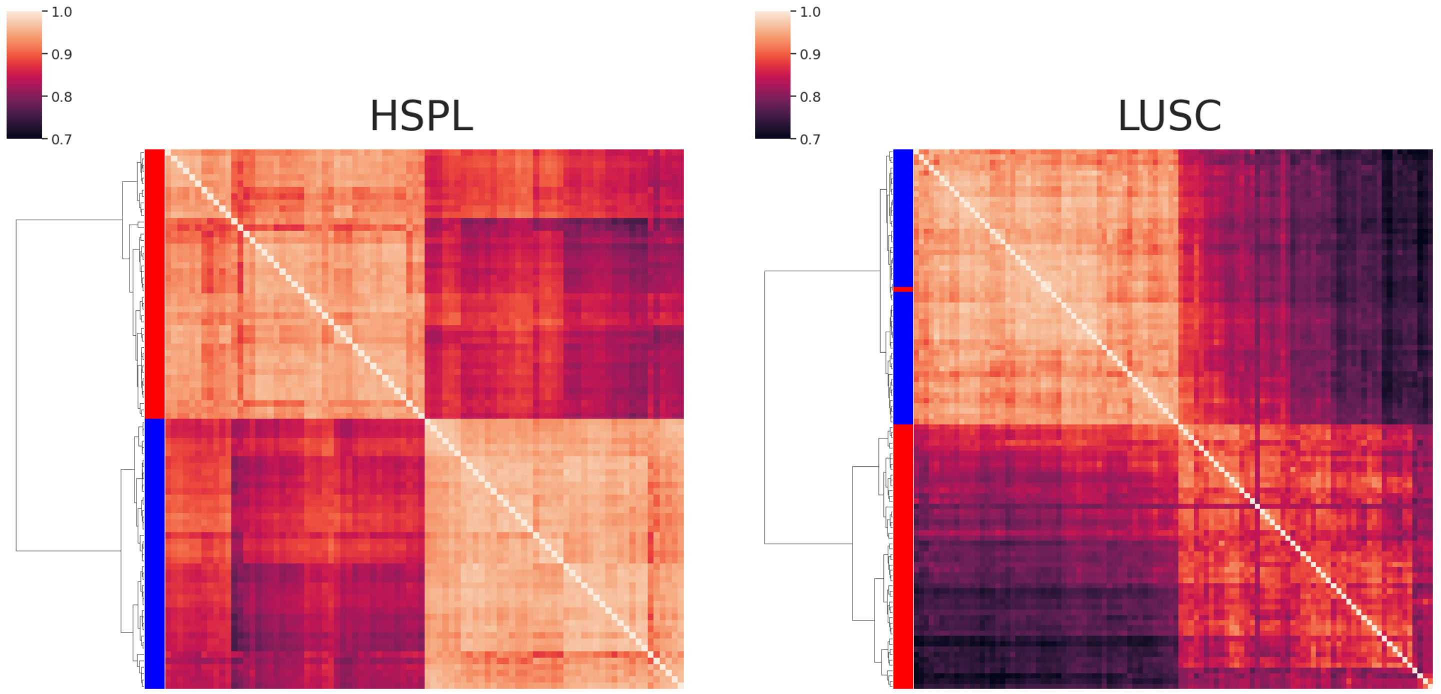

Figure A: **Heat maps for the HSPL and LUSC data sets.** Heat maps show the logCPM correlation of samples. Rows and columns were ordered using hierarchical Ward clustering.

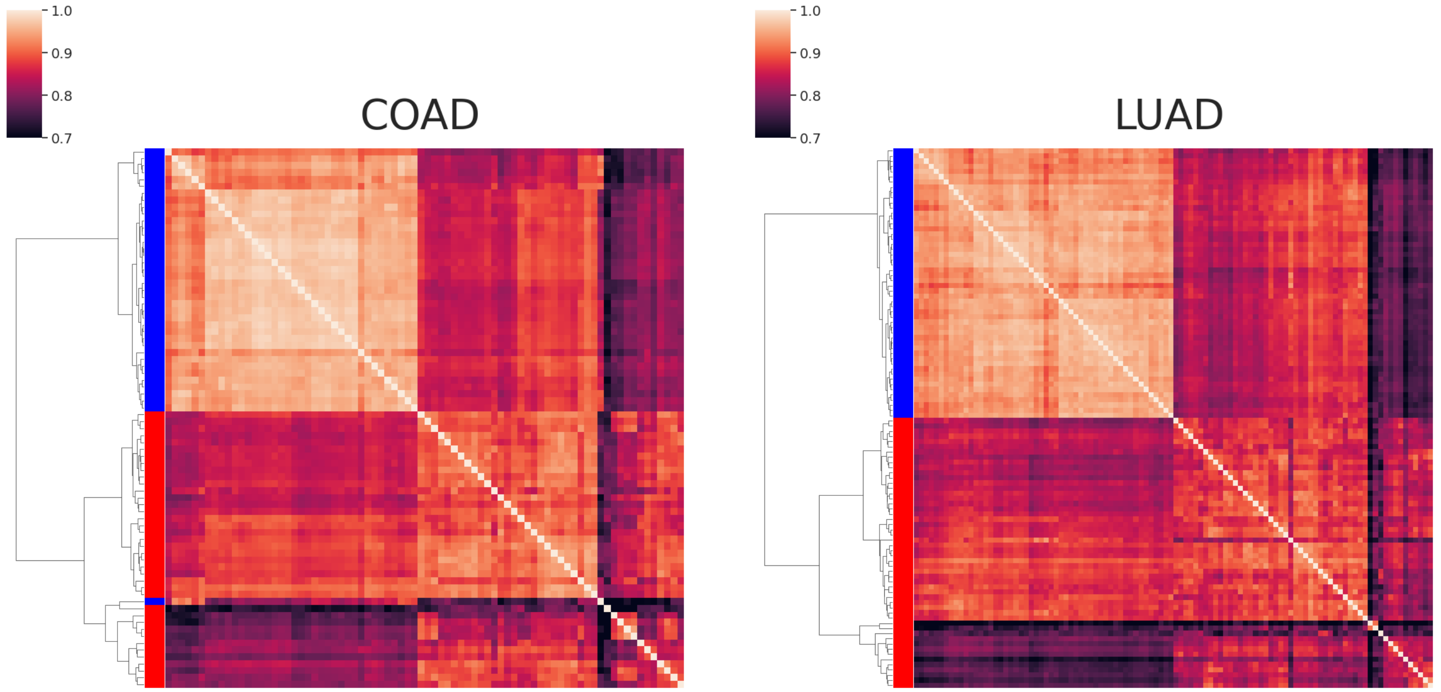

Figure B: **Heat maps for the COAD and LUAD data sets.** Heat maps show the logCPM correlation of samples. Rows and columns were ordered using hierarchical Ward clustering.

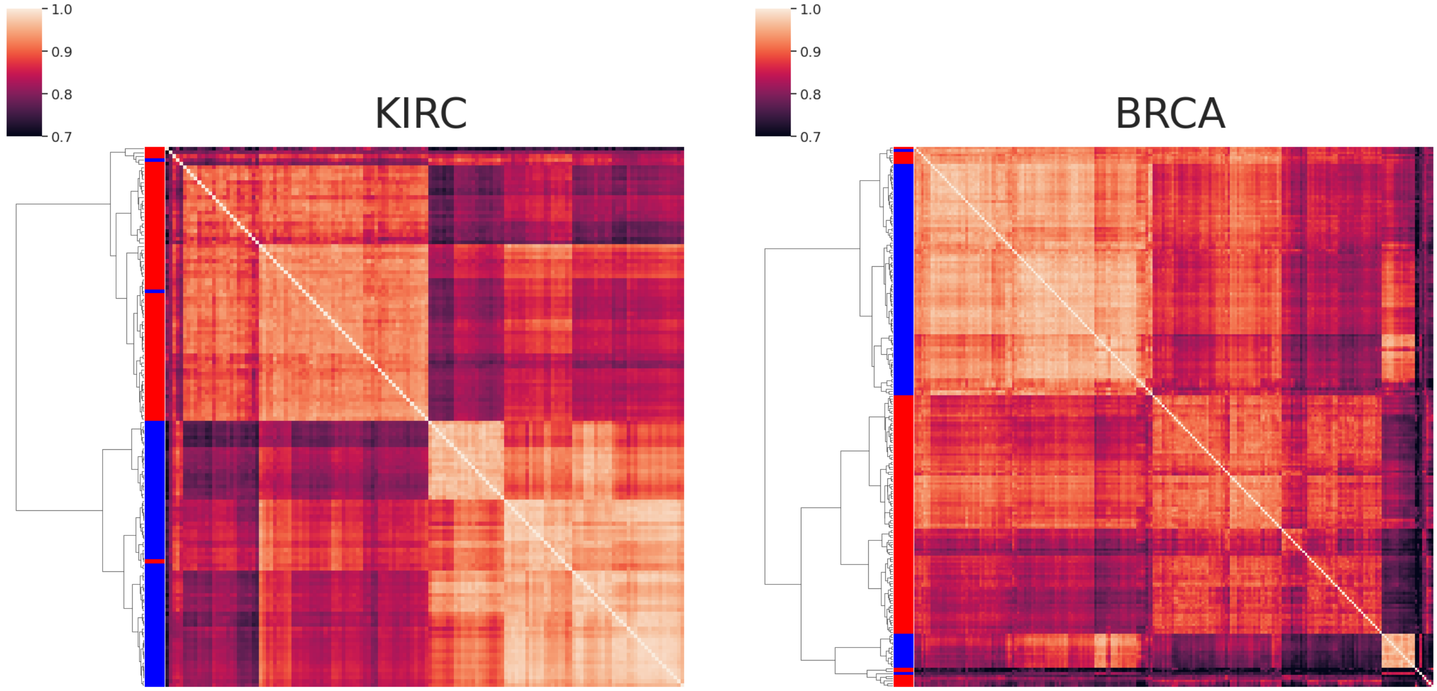

Figure C: **Heat maps for the KIRC and BRCA data sets.** Heat maps show the logCPM correlation of samples. Rows and columns were ordered using hierarchical Ward clustering.

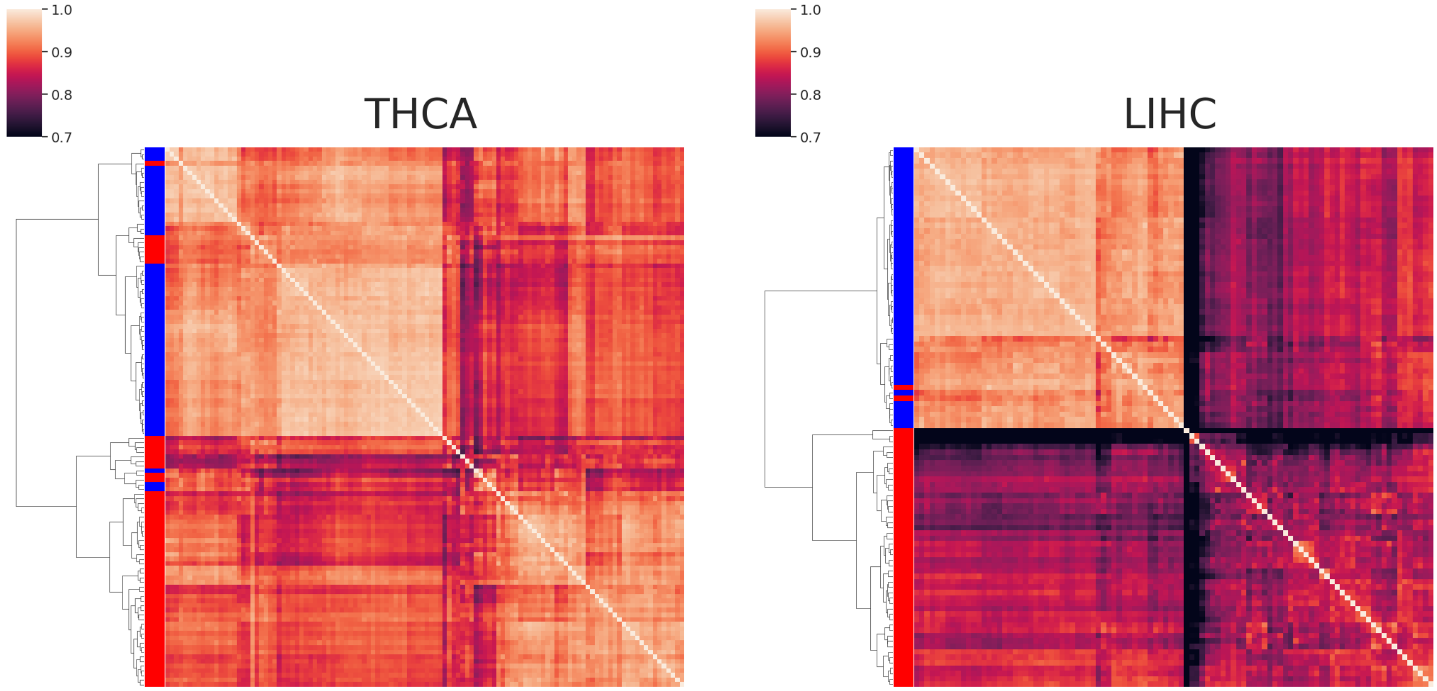

Figure D: **Heat maps for the THCA and LIHC data sets.** Heat maps show the logCPM correlation of samples. Rows and columns were ordered using hierarchical Ward clustering.

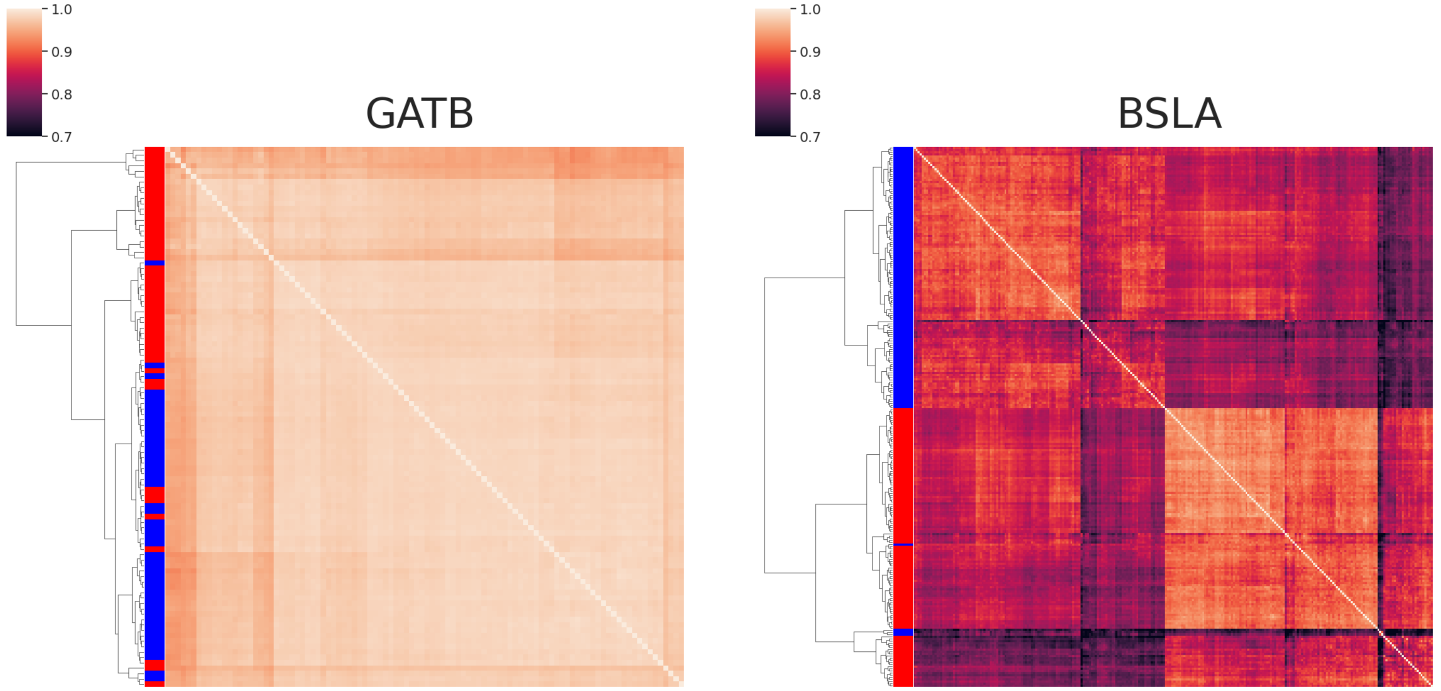

Figure E: **Heat maps for the GATB and BSLA data sets.** Heat maps show the logCPM correlation of samples. Rows and columns were ordered using hierarchical Ward clustering.

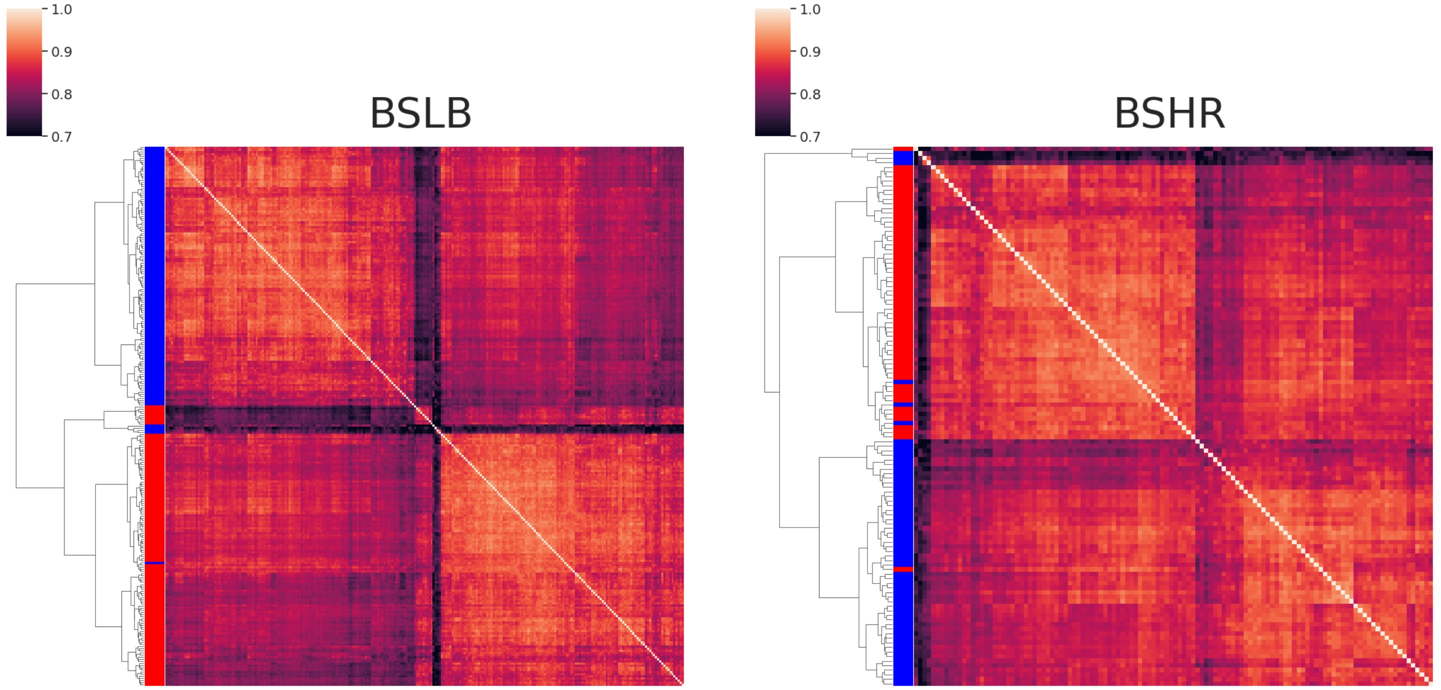

Figure F: **Heat maps for the BSLB and BSHR data sets.** Heat maps show the logCPM correlation of samples. Rows and columns were ordered using hierarchical Ward clustering.

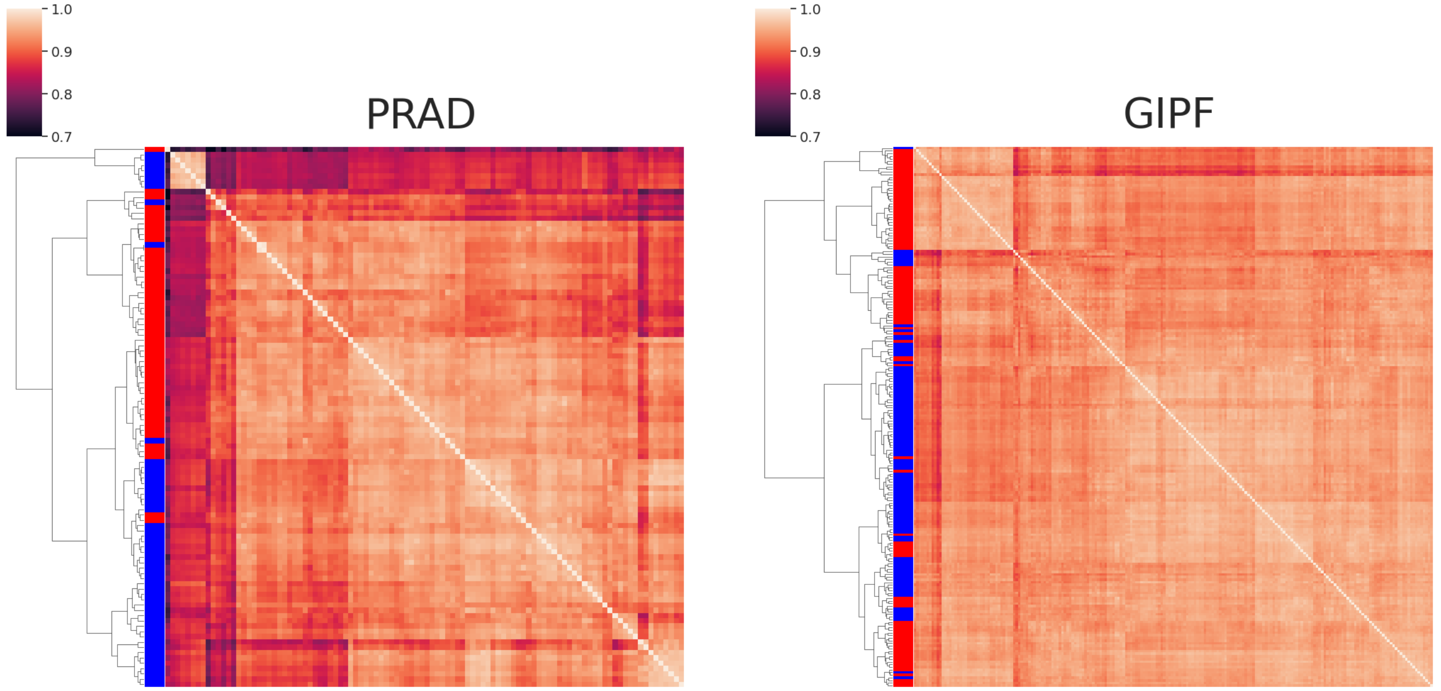

Figure G: **Heat maps for the PRAD and GIPF data sets.** Heat maps show the logCPM correlation of samples. Rows and columns were ordered using hierarchical Ward clustering.

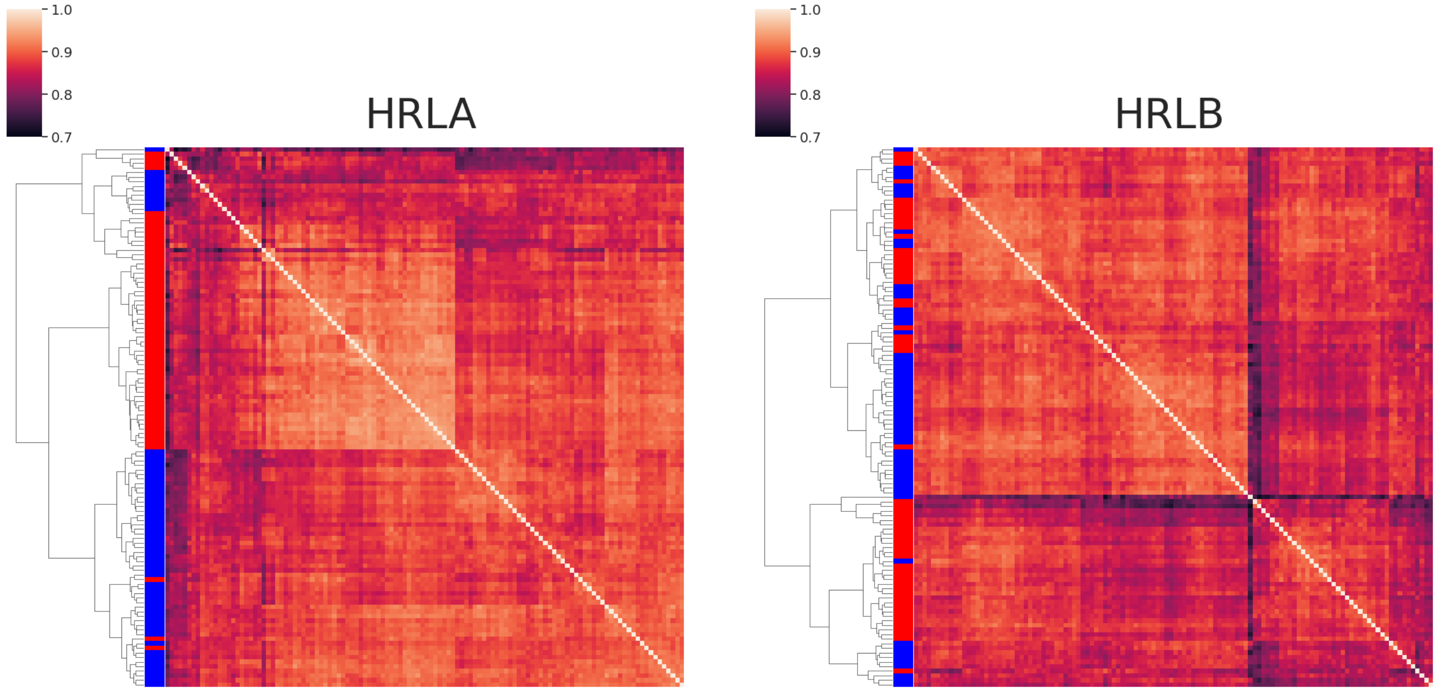

Figure H: **Heat maps for the HRLA and HRLB data sets.** Heat maps show the logCPM correlation of samples. Rows and columns were ordered using hierarchical Ward clustering.

1.2 Fold change figures for the remaining data sets

Fig I–P show fold change estimates of all genes expressed in the respective data sets, unless DESeq2 was unable to estimate a fold change. Blue dots represent the ground truth estimate from the full data set. Gray (red) bars represent the interquartile range of estimates obtained from 100 subsampled cohorts of size $N = 3$ ($N = 15$). The horizontal dashed line shows the logFC threshold used to define DEGs. The legend lists the number of bars that cross the dashed line. See main text for SNF2 and LMAB data sets.

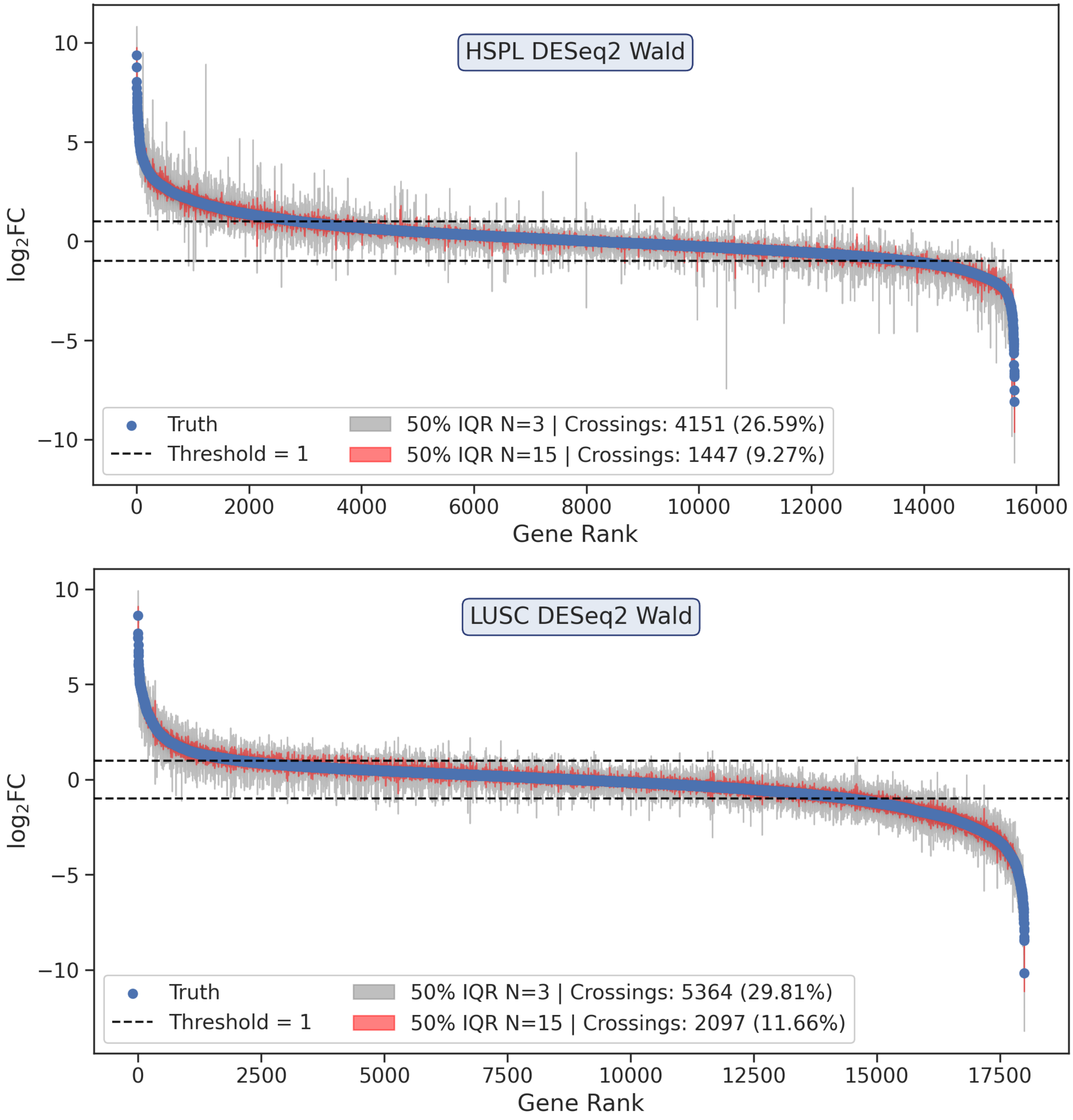

Figure I: **Fold change estimates for the HSPL and LUSC data sets.**

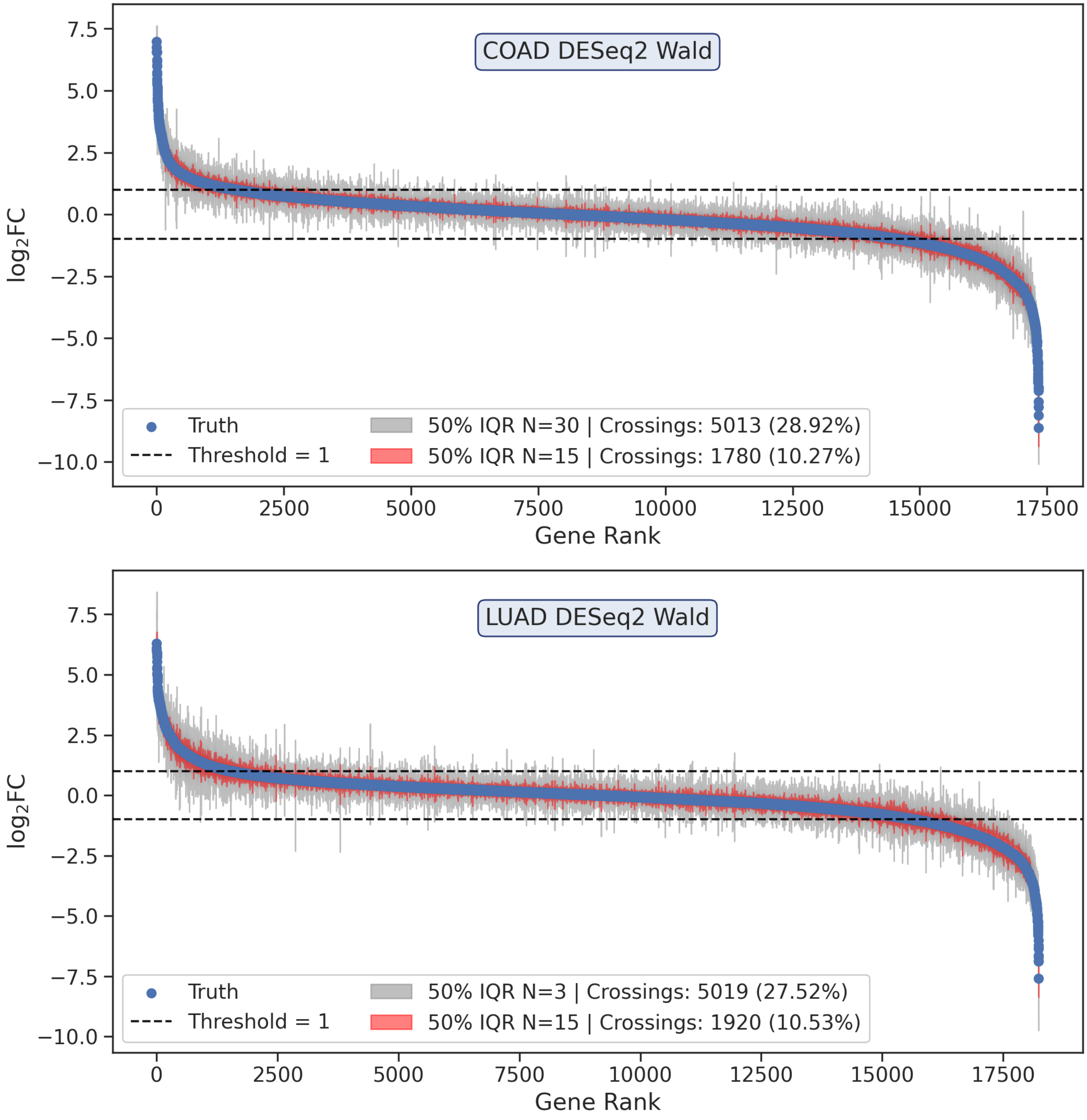

Figure J: **Fold change estimates for the COAD and LUAD data sets.**

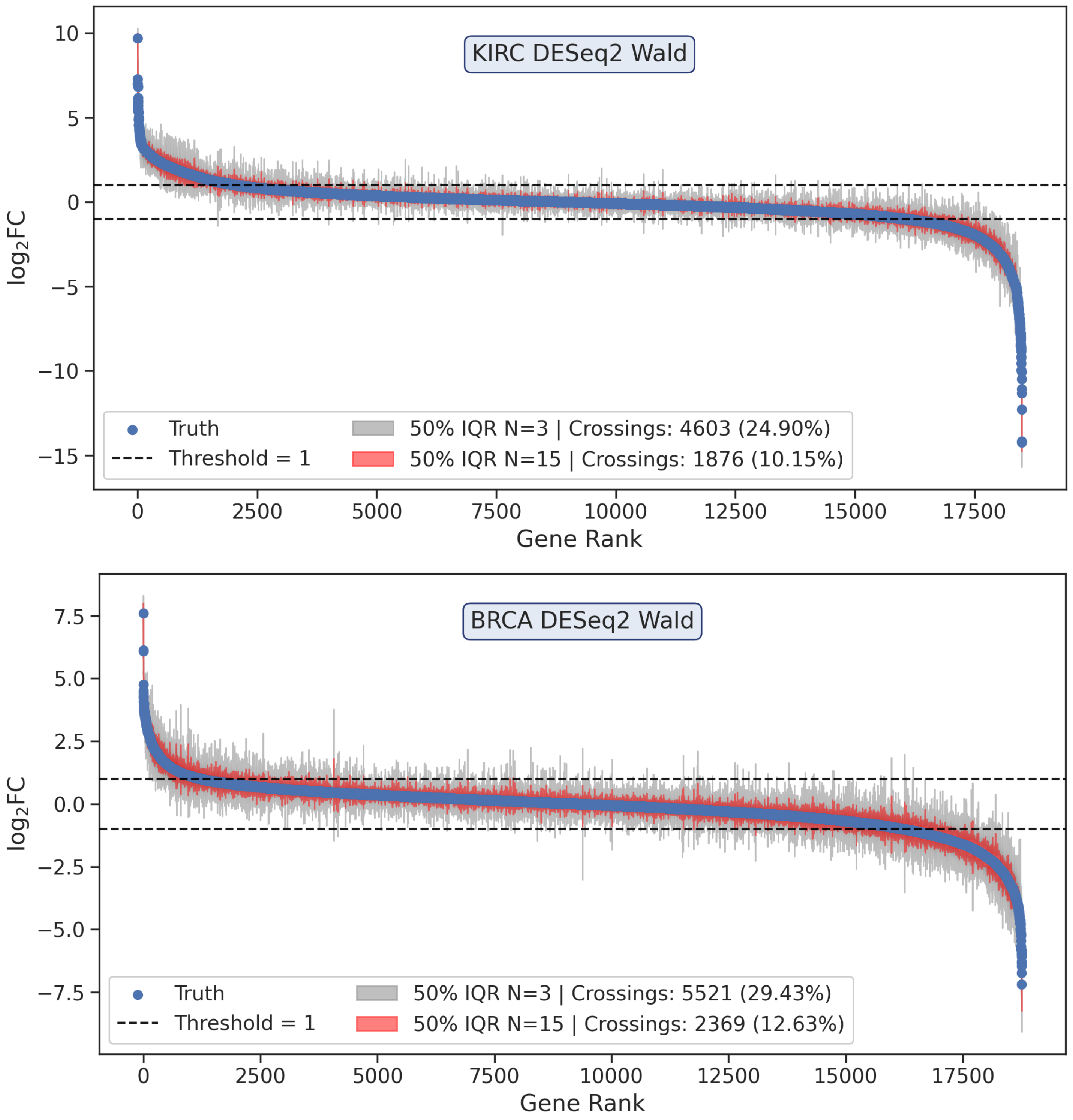

Figure K: **Fold change estimates for the KIRC and BRCA data sets.**

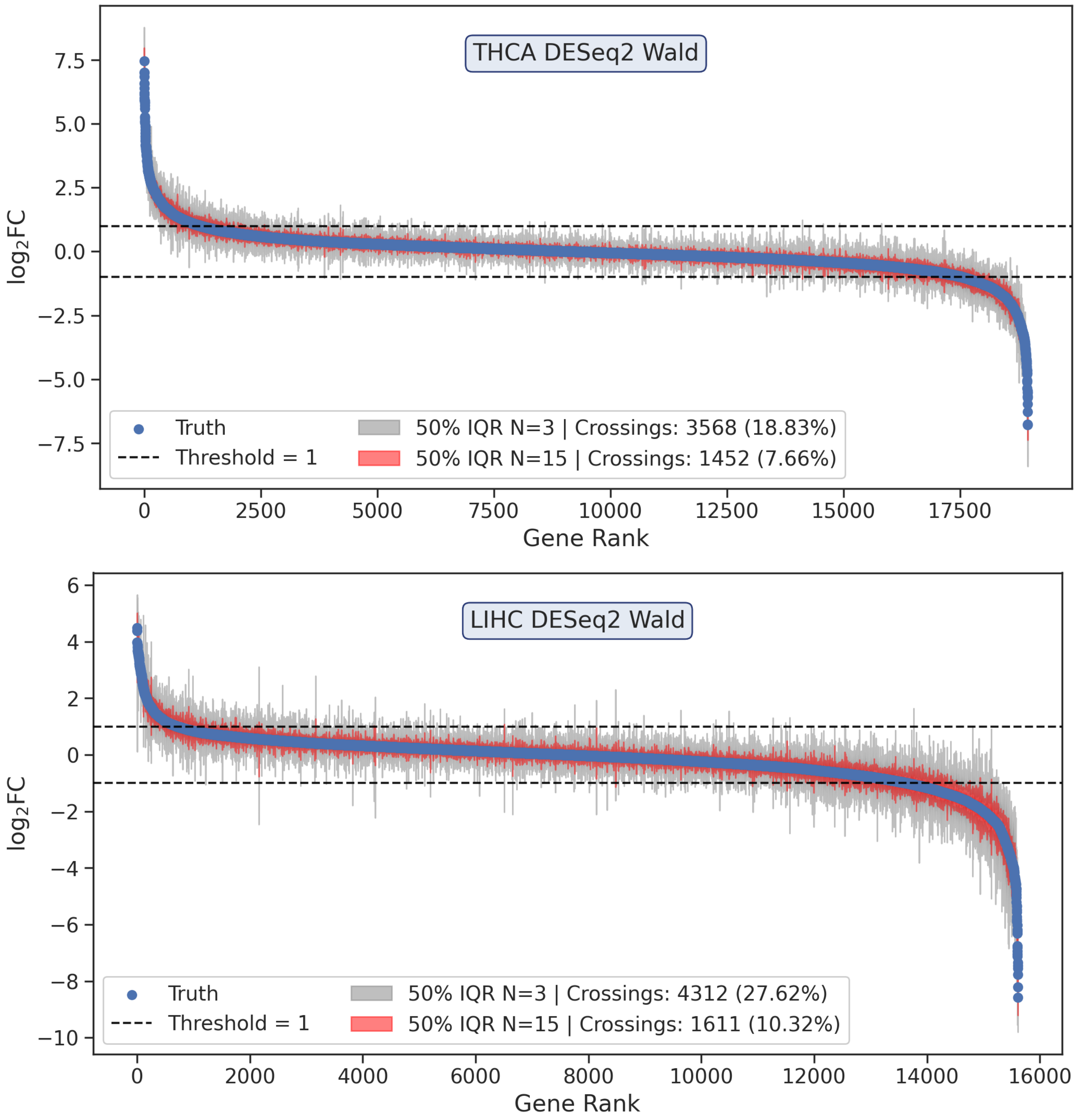

Figure L: **Fold change estimates for the THCA and LIHC data sets.**

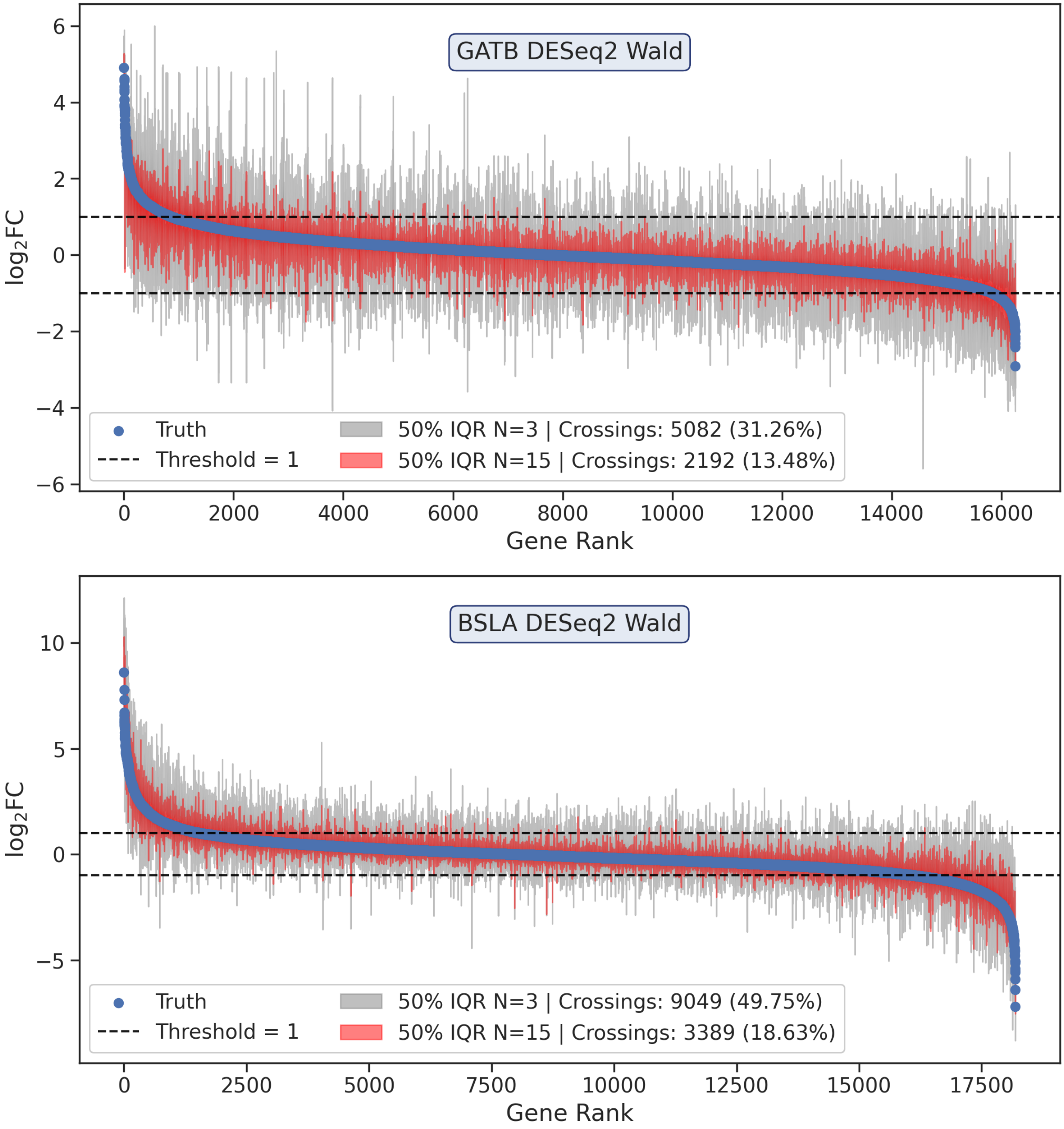

Figure M: **Fold change estimates for the GATB and BSLA data sets.**

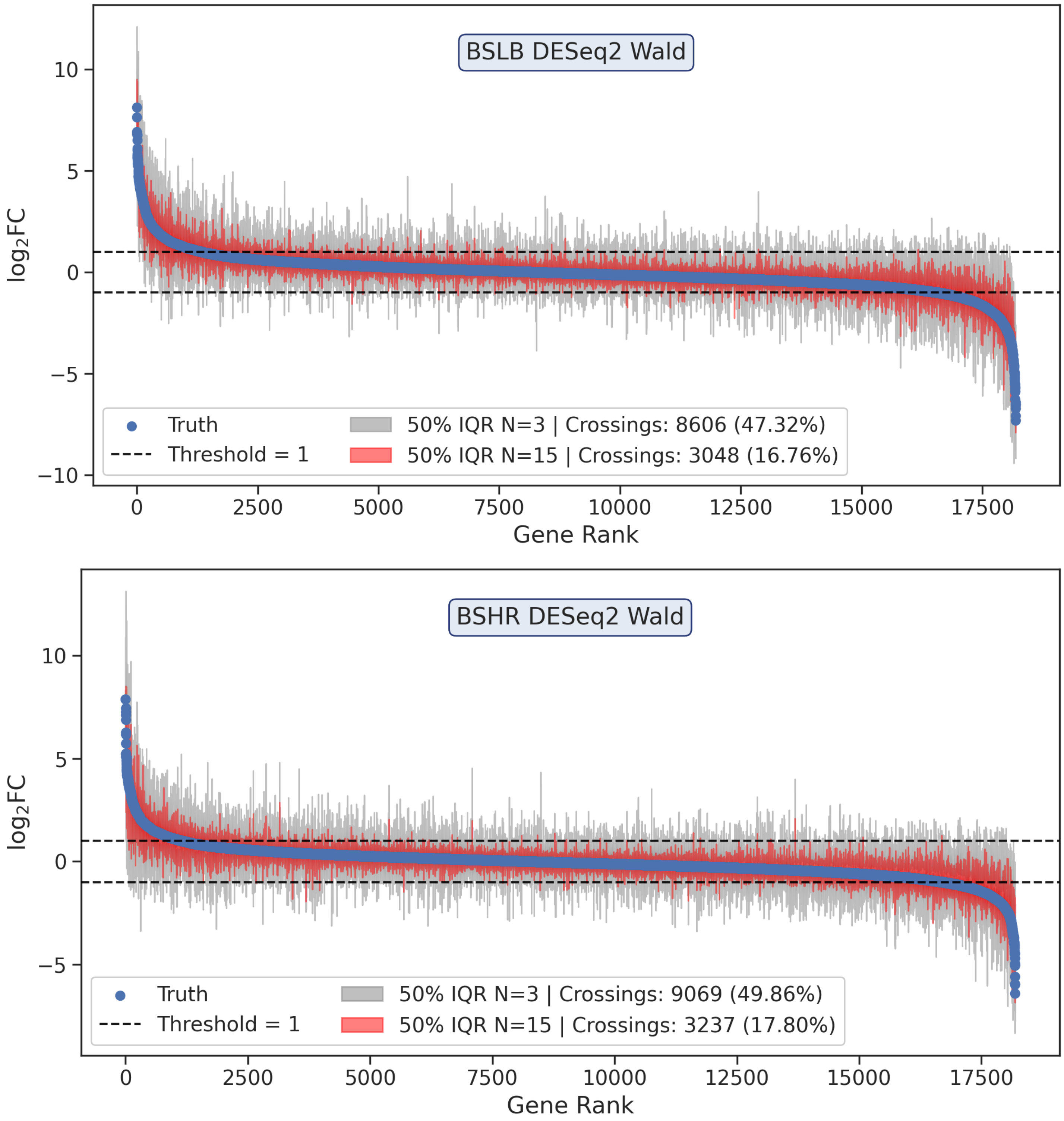

Figure N: **Fold change estimates for the BSLB and BSHR data sets.**

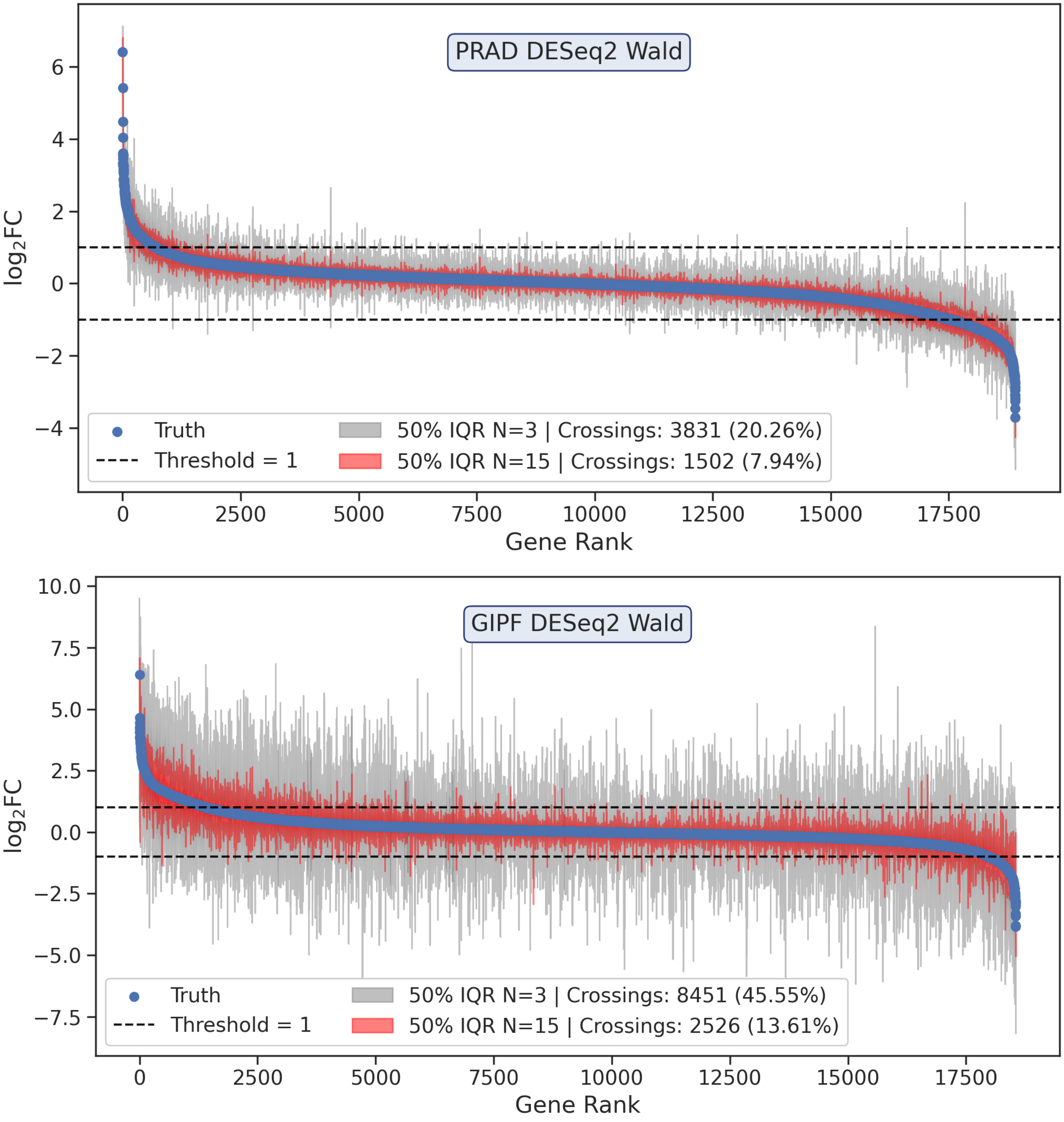

Figure O: **Fold change estimates for the PRAD and GIPF data sets.**

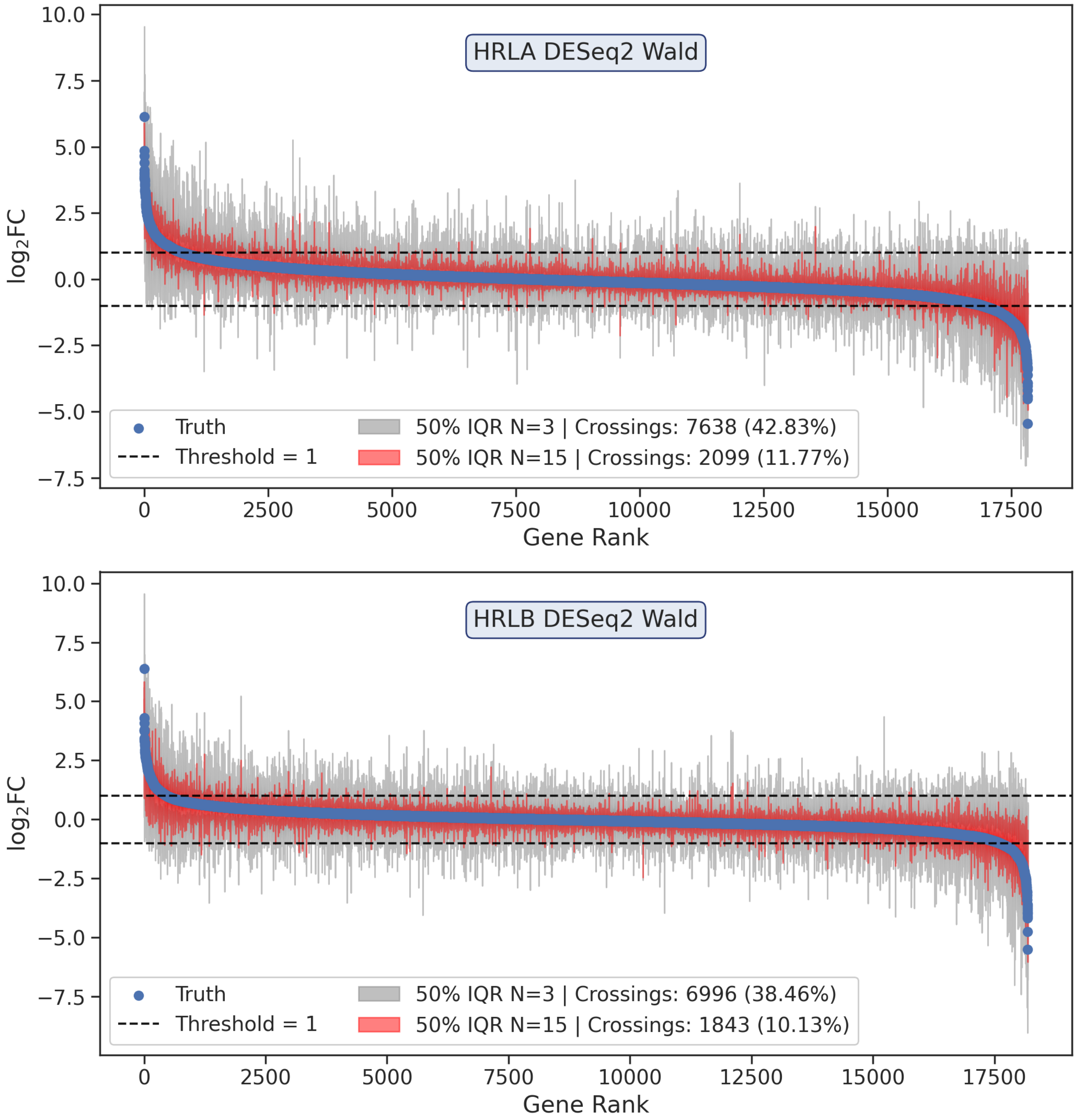

Figure P: **Fold change estimates for the HRLA and HRLB data sets.**
