# Supplementary material for: Replicability of bulk RNA-Seq differential expression and enrichment analysis results for small cohort sizes
Source: PLoS Comput Biol. 2025 May 5;21(5):e1011630. doi: 10.1371/journal.pcbi.1011630 (PMC12077797; doi:10.1371/journal.pcbi.1011630)
Supplement: Attachment — Submitted filename: 20231126.pdf [file pcbi.1011630.s004.pdf]

Summary

This study presents an ensemble of experiments to understand the nature of reproducibility in bulk RNA-Seq data from cancer studies. The main experiments and their interpretations are,

1. Setup: Run three differential testing methods on data subsampled to different cohort sizes. Use the intersection of DEGs from the three methods run on the complete data as a proxy for ground truth. Finding: Replicability can be low, but precision is consistently high. Therefore, seemingly underpowered cancer studies contribute genuine associations to the literature, they're just not the complete story.
2. Setup: The same as above, but for gene set analysis. Finding: Most of the trends as a function of sample size agree with the previous experiment. However, none of the methods achieved their nominal FDR.
3. Setup: Fit edgeR with an aggregated dataset, then estimate LFC for each gene using subsamples of different sizes. Finding: The LFC estimates can be substantially off when using small cohorts.
4. Setup: Run two DE analyses where the only difference is whether an outlier removal method was used first. Finding: Outlier removal slightly reduces precision but substantially increases recall.

Evaluation

I found this study well-motivated and the experimental data they generate relevant to the ongoing discussion of reproducibility in high-throughput biology. My primary concern is that it's unclear whether its explanation for the high observed precision (small studies discover a subset of true DEGs from a larger whole) generalizes to all "biologists working with RNA-Seq data from small cohorts sampled from a heterogeneous population." It may very well be a general phenomenon, and I think it's interesting to document it even in a specific experimental setup, but making the general claim requires a bit more care.

1. The writing is generally easy to follow with high-quality figures. I also briefly skimmed the paper by Cui et al. (2021). That study inspires the current manuscript; however, this follow-up substantially improves the breadth and quality of execution.
2. The results are a mix of obvious-in-retrospect (e.g., larger sample size -> higher recall) and quite surprising (e.g., low FP even in small sample sizes, also low FP even with inflated effect estimates). Further developing the interpretation of these surprising phenomena would strengthen the manuscript. My following points provide specific examples.
    a. My impression is that the precision is so high in these experiments because there is such a large number of "ground truth" genes (line 292 and Table 1). This raises two questions: (1) Is the method of declaring ground truth genes sound? (2) Is this phenomenon specific to paired-design studies in TCGA? For (2), I see your argument that single-cell studies are not applicable, and I would appreciate some additional context for the current data. For example, it might be worth exploring the typical effect sizes in TCGA. For (1), it could be worth reporting model goodness-of-fit, the influence of subsampling with replacement (which violates independence assumptions), or the stability of ground truth sets as sample size/number of methods increase.
    b. It seems counterintuitive that, in small cohorts, the effect estimates are inflated (Fig. 5), but this doesn't inflate the false positive rate (Fig. 2). Can you explain this? Is it that the effects are high variance but still unbiased? Perhaps you can find a way to simultaneously visualize effect sizes and whether they eventually lead to false positives.
3. The robustness-to-outlier experiments felt out of place. This is perhaps because its results are confined to one panel of one figure, and it doesn't appear in either the abstract or the takeaway bullet points.

4. I assume you are subsampling in a way that preserves matching in the paired design. I couldn't find this explicitly in the methods, though. Could you include this/point me to the relevant discussion?
5. Related to my comment about independence above, isn't line 760-761 not strictly true because of this? You underestimate the population heterogeneity, but the method still believes it has more independent samples. It might underestimate the within-group variance and lead to artificially inflated test statistics.
6. The replication code, data, and notebooks are high quality — good file names, split into readable functions, easy to access intermediate results. I appreciate this effort to implement the experiments logically and transparently.

Minor Comments

1. There are a few points where the methods section feels like it should be read before the results (e.g., references to equations, introduction of acronyms). I recommend double-checking that cross-references between sections flow well.
2. On a related note, some of the terminology in the Results, like "replicability" and "gene prevalence," does not seem widely used. I recommend double-checking that as soon as these terms are introduced, a reference is given to the corresponding equation in the methods section.
3. In line 471, you write "formal (post-hoc)." I thought post-hoc was the informal approach. Also, in other places, you write "post hoc" instead of "post-hoc."
4. Line 560 is missing a "FC" in.
5. The y-axis in Figure 19 makes it look like there are cohorts where 1/10 DE genes are found. Is this meant to be 0?
