# Supplementary material for: Replicability of bulk RNA-Seq differential expression and enrichment analysis results for small cohort sizes
Source: PLoS Comput Biol. 2025 May 5;21(5):e1011630. doi: 10.1371/journal.pcbi.1011630 (PMC12077797; doi:10.1371/journal.pcbi.1011630)
Supplement: Attachment — Submitted filename: Degen and Medo Response to reviewers.pdf [file pcbi.1011630.s005.pdf]

Response to the reviewers

Peter Degen and Matúš Medo

May 28, 2024

Contents

1 Reviewer 1 **3**
1.1 Evaluation 3
1.2 Minor comments 6

2 Reviewer 2 **7**

3 Reviewer 3 **8**

We appreciate the reviewers' comments and thank them for their constructive feedback.

Changes are highlighted in red and blue in the revised manuscript. The following is a list of the most important changes:

- The methods section was moved from the end to after the introduction section.
- The enrichment results were moved to the last subsection of the results section.
- Figures and most of the text related to outlier patient removal and gene prevalence were transferred to the supplement. The main text has a short new section on outlier removal to refer to the supplement. Gene prevalence results are briefly mentioned in the main results section.
- Discussion of effect size inflation was expanded and the last figure in the results section was changed to focus solely on effect size results.
- The Wilcoxon signed-rank test was added as a fourth differential expression testing method (results shown in supplement). This only includes DEG analysis, not enrichment analysis.
- A comparison of paired and unpaired testing was added to the supplement.
- In the main text, a short section was added to discuss the prostate adenocarcinoma (PRAD) data set, which behaves notably differently from the other cancer data sets.
- Related to the previous point, a limited exploration of two additional, non-cancer data sets was added (results shown in supplement), bringing the total number of data sets to nine. The two new data sets behave similarly to PRAD.

- A minor redefinition of two of our performance metrics (MCC and precision) was made. To address the issue of division by zero when no differentially expressed genes are identified, rather than considering the metric undefined and excluding it from the median calculation, we opted to assign the metric a value of zero. This adjustment has a minimal impact on the majority of results, except for the scenario involving edgeR QLF for very small cohort sizes. We believe this modification better captures the behavior in this case, which, for most cohorts, identifies zero DEGs.

1 Reviewer 1

1.1 Evaluation

> My primary concern is that it's unclear whether its explanation for the high observed precision (small studies discover a subset of true DEGs from a larger whole) generalizes to all "biologists working with RNA-Seq data from small cohorts sampled from a heterogeneous population." It may very well be a general phenomenon, and I think it's interesting to document it even in a specific experimental setup, but making the general claim requires a bit more care.

We agree that the original phrasing of our conclusion is too general given the cancer-specific scope indicated by our title. As we are making an affirmative claim rather than a cautionary one (DEGs can be reliable for small cohort sizes), we should be more hesitant to extrapolate our results to cases not covered by our data sets. We have adjusted the phrasing in the conclusion to emphasize that our results are based on TCGA data. We also note in the discussion (line 405 in the highlighted manuscript) that false positives may become more prevalent when very few DEGs are identified for very small cohorts (see also our response to the ground truth discussion at the end of this page).

> The results are a mix of obvious-in-retrospect (e.g., larger sample size -> higher recall) and quite surprising (e.g., low FP even in small sample sizes, also low FP even with inflated effect estimates). Further developing the interpretation of these surprising phenomena would strengthen the manuscript.

This is a useful observation. We have expanded our results and discussion of effect size inflation accordingly. We have also rephrased the abstract and conclusion to better reflect these surprising findings.

> a. My impression is that the precision is so high in these experiments because there is such a large number of "ground truth" genes (line 292 and Table 1). *(Author's note: The number of ground truth DEGs is depicted in Fig. 1 of S1 Appendix, not in Table 1.)*

The reviewer is right to be concerned about the number of ground truth DEGs. We tried to address this in the original manuscript by: (1) Introducing a fold change cut-off and (2) reporting the expected precision under the null model of random DEG selection.

Without a fold change cutoff, the expected precision of randomly chosen DEGs is high ($\approx$ 70%, see Supplementary Fig. 29A in S1 Appendix) and the high precision of the identified DEGs ($\approx$ 95%) is thus also influenced by the large ground truth size (Supplementary Fig. 1). However, when we formally apply a fold change threshold, the expected random precision drops to 11% due to a substantially smaller ground truth size, yet the empirical precision remains high at $\approx$ 85-95% (Fig. 29B). This indicates that, for most data sets, the methods themselves are reasonably precise even for small cohort sizes. For convenience, we attach a cropped version of Supplementary Fig. 29 below.

At the same time, it is true that one of our TCGA data sets (PRAD) exhibits both the lowest precision and the lowest number of ground truth DEGs above a fold change. Similarly, the number of DEGs above a fold change identified for subsampled PRAD cohorts is very low (Supplementary

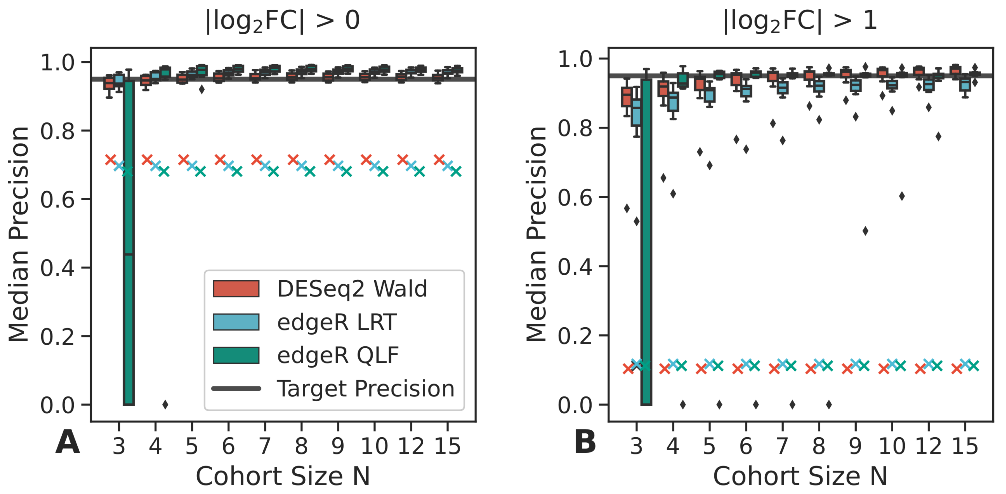

Figure 1: Cropped version of Supplementary Fig. 29. Crosses depict the median expected precision for randomly selected DEGs. Most low-precision outliers (black diamonds) originate from the PRAD data set. **A:** no fold change threshold. **B:** with a formal fold change threshold.

Fig. 2). In the revised results and discussion, we now make a note that false positives may become prevalent when very few DEGs ($\lesssim 100$) are identified for very small cohorts ($N \lesssim 7$). This observation is additionally based on two new, non-TCGA data sets, for which we conducted a limited exploration (Supplementary Fig. 28). Both data sets exhibit a similar number of DEGs as PRAD and display similarly lower precision for $N \lesssim 7$.

> This raises two questions: (1) Is the method of declaring ground truth genes sound? [...] For (1), it could be worth reporting model goodness-of-fit, the influence of subsampling with replacement (which violates independence assumptions), or the stability of ground truth sets as sample size/number of methods increase.

Supplementary Fig. 1 in S1 Appendix shows that the intersection of the ground truths declared by the three methods (Wald, QLF, LRT) is very high for all tested scenarios. This indicates that the ground truth is reasonably robust to the choice of the DE method. For the revised manuscript, we have added the Wilcoxon signed-rank test as a fourth method. Supplementary Fig. 21 shows that the intersection of truth DEGs among all four methods is still very high which further supports the ground truth robustness.

We believe that there may have been a misunderstanding regarding our subsampling procedure (we have changed some phrasings in the revised version for clarity): (1) For each cohort, subsampling is done without replacement; each cohort thus consists of unique patients. (2) However, once a cohort has been subsampled, all of its patients are placed back into the parent data set before a new cohort is subsampled. This means that there can be repeating patients between two different cohorts. Because of (1), our binary classification metrics (which depend on the ground truth) are not negatively affected by our subsampling procedure. Because of (2), our computed replicability metrics (which do not depend on the ground truth) are inflated by a small amount, as can be seen in Supplementary Fig. 26.

(2) Is this phenomenon specific to paired-design studies in TCGA? For (2), I see your argument that single-cell studies are not applicable, and I would appreciate some additional context for the current data. For example, it might be worth exploring the typical effect sizes in TCGA.

The new Supplementary Fig. 27 shows our main performance metrics computed for unpaired TCGA data. As expected, the metrics are generally worse compared to paired designs, though the main conclusions are still broadly valid (high precision for small cohorts).

As mentioned above, we also conducted a limited exploration of two additional, non-TCGA data sets (active tuberculosis and idiopathic pulmonary fibrosis). However, given our available resources, broadening our scope to cover substantial ground beyond cancer was not possible for the revision. The initial choice of TCGA was guided by two criteria: 1) public accessibility of sufficiently large data sets, and 2) the authors' affiliation with the radiation oncology lab at the University of Bern, which provided access to a network of colleagues with expertise in cancer research.

b. It seems counterintuitive that, in small cohorts, the effect estimates are inflated (Fig. 5), but this doesn't inflate the false positive rate (Fig. 2). Can you explain this? Is it that the effects are high variance but still unbiased? Perhaps you can find a way to simultaneously visualize effect sizes and whether they eventually lead to false positives.

Our interpretation is as follows: when using a post hoc fold change threshold, the false positive rate is substantially inflated (Fig. 3C) as a consequence of effect size inflation. However, when we formally test a given fold change threshold, the observed FDR is close to the expected FDR (Fig. 3B), though still displaying some discrepancy for $N \lesssim 6$. The formal testing procedure requires a higher standard of evidence before a gene is declared significant and of sufficient effect size. Genes that barely pass the threshold due to random effect size inflation are thus unlikely to pass the formal testing procedure, resulting in better precision of this approach. We have expanded our discussion of this phenomenon and added additional panels in the last main figure to demonstrate this point more clearly.

3. The robustness-to-outlier experiments felt out of place. This is perhaps because its results are confined to one panel of one figure, and it doesn't appear in either the abstract or the takeaway bullet points.

We agree. In an earlier stage of the manuscript, the outlier discussion was more prominent, but eventually it was reduced to make space for other results. For the revised version, we have moved the bulk of the outlier discussion to the supplementary materials.

4. I assume you are subsampling in a way that preserves matching in the paired design. I couldn't find this explicitly in the methods, though. Could you include this/point me to the relevant discussion?

Yes, the subsampling preserves the matching of patient samples for paired testing. We have now added this point to the methods section (line 86 int he highlighted manuscript).

Related to my comment about independence above, isn't line 760-761 not strictly true because of this? You underestimate the population heterogeneity, but the method still believes it has more independent samples. It might underestimate the within-group variance and lead to artificially inflated test statistics.

The argument made in that line was not phrased very well and we have changed it accordingly. The existence of repeated patients between different subsampled cohorts implies that these cohorts are more similar than what we would observe in a real scenario, where no two patients are identical. Hence, we expect that the replicability between two subsampled cohorts should be slightly inflated compared to two real cohorts. We confirm this in the newly generated Supplementary Fig. 26 which shows that the inflation is even smaller than expected, 0.1–1%.

1.2 Minor comments

1. There are a few points where the methods section feels like it should be read before the results (e.g., references to equations, introduction of acronyms). I recommend double-checking that cross-references between sections flow well.
2. On a related note, some of the terminology in the Results, like "replicability" and "gene prevalence," does not seem widely used. I recommend double checking that as soon as these terms are introduced, a reference is given to the corresponding equation in the methods section.

Indeed, the methods section was originally written to appear before the results section. For the revised version, we have changed it back to the original order and moved a few technical details of the original methods section to the supplement. We also moved the gene prevalence results to the supplement, as we do not think it is a straightforward metric to interpret. This also allows us to focus more on the effect size inflation for the final figure.

3. In line 471, you write "formal (post-hoc)." I thought post-hoc was the informal approach. Also, in other places, you write "post hoc" instead of "post-hoc."
4. Line 560 is missing a "FC" in.
5. The y-axis in Figure 19 makes it look like there are cohorts where 1/10 DE genes are found. Is this meant to be 0?

The original phrasing of line 471 was intended to present results for both post hoc and formal thresholding in the same sentence, but we realize now the ambiguity of the phrasing and have changed it in the revised version. We also fixed the minor mistakes mentioned in points 4 and 5.

2 Reviewer 2

> Main concern: The analysis was purely made on TCGA data. It is unclear if the results can generally capture the robustness level for RNA-seq DEG analysis. The authors are expected to make a systematic analysis on at least five independent RNA-seq data cohorts. It will be better to include samples of different organ types such as blood and brain samples.

In the original manuscript, we used seven independent cohorts from seven different organ types (lung, liver, breast, kidney, colorectal, prostate, thyroid). We checked every available organ type at the TCGA and found that these seven are the only ones with sufficient sample size for our purposes. However, the reviewer rightly points out that all cohorts originate from TCGA. Thus, generalizability to RNA-Seq data from non-cancer samples remains an open question. Due to the broad applicability of RNA-Seq experiments, it is not possible to fully answer this question in the scope of a single study. We also conducted a limited exploration of two additional, non-TCGA data sets (active tuberculosis and idiopathic pulmonary fibrosis) and found similar results to unpaired analysis of the TCGA data (see Figs. 27 and 28 in the supplement). However, given our available resources, broadening our scope to cover substantial ground beyond cancer was not possible for the revision. The results for the two newly added data sets are shown in Supplementary Fig. 28 and referred to in the main text as part of our discussion of the PRAD data set.

> Also, DESeq and EdgeR are used for DEG for pseudobulk based analysis of scRNA-seq data. It will be good if an evaluation of this part could be included.

Due to the substantially increased cost and computational complexity of single-cell experiments, it is not viable to repeat our analysis for this data modality. For example, certain processing steps such as the integration of different batches require huge amounts of memory, necessitating the use of HPC clusters. Repeating this kind of analysis thousands or even hundreds of times for subsampled cohorts exceeds user limits on common university clusters. Directly transferring our methodology from bulk RNA-Seq to single-cell RNA-Seq is thus difficult in practice.

That said, we are happy to inform the reviewer that our newest project is indeed related to the comment at hand. Using an in-house single-nuclei RNA-Seq data set as a case study, we investigate the robustness of different analysis pipelines and tools, including the performance of pseudobulk differential expression analysis methods. We plan to submit a manuscript for this project later this year.

> Minor concern: Nonparametric Mann Whitney test should be compared.

This is an interesting point, in particular regarding a recent paper [2] which suggests this test for “RNA-seq studies with large sample sizes”. We have now added this test in the revised supplement (under the name of Wilcoxon signed-rank test). As this is only a test for computing p-values and not a dedicated differential expression analysis tool that normalizes count data and computes fold changes, we used DESeq2 for these steps. This makes it possible to use the Wilcoxon test with post hoc fold change thresholding. Supplementary Fig. 22 in S1 Appendix shows that this test performs worse than DESeq2 and egdeR; thus we recommend against using it when sample sizes are small.

3 Reviewer 3

1. The simulation study is random sampling a subset of RNA-seq samples from the total cohorts. However, random sampling may not a good strategies. Covariates of the cohort such as gender, sex may confound the analysis results.

The reviewer is right to point out the importance of controlling for confounders. However, this can be difficult when sample sizes are very small, which is the case we are interested in. When the number of confounders becomes comparable or even exceeds the number of observations, the design matrix can become rank-deficient, making it impossible to control for the confounders in the statistical model.

Moreover, we decided against actively balancing the cohorts due to the limited data available. For example, there are difficulties with stratified sampling for very small sample sizes (e.g., it is impossible to have a balanced ratio of males and females if $N = 3$). Alternatively, we could have constructed same-sex cohorts and computed our metrics for each sex separately. However, this would have split our already limited parent data sets in half, e.g. from 52 patients to 26 patients, which we deemed insufficient for our purposes. The largest data set, BRCA, has 111 patients but 110 are female, which makes this stratification ineffective. Ultimately, random sampling was the most practical approach for our purposes.

2. In addition the simulation study is not real simulation conceptually but still real data analysis. Usually simulation are generated from a parametric model with underlying gene expression and FC know in advance to evaluate the DE performance under different sample size

We acknowledge the terminological difference in our use of the word “simulated”. To minimize confusion, we have now replaced this word with “subsampled” in the revised version.

We also attempted to use completely synthetic data generated from parametric distributions in a preliminary stage of our study. However, generating realistic data proved to be difficult because the model needed to be overly complex to match the level of heterogeneity observed in the real TCGA samples. We ultimately concluded that there is little benefit in having complex synthetic data when we can construct an empirical ground truth that makes fewer assumptions about the data.

3. The major conclusions large sample size results in better reproducibility and small cohort will have poor reproducibility is well-known common sense.

It is true that small replicability of small cohorts is expected, as has been reported recently, for example, by [1]. However, our main conclusion is not only that larger sample sizes have higher replicability, but the rather surprising result that even small sample sizes can exhibit high precision of DEGs, despite effect size inflation and low inter-experiment replicability. As reviewer 1 pointed out, the original manuscript could have emphasized this point more clearly. We tried to achieve that in the revised version.

References

[1] Weitong Cui et al. "Effect of high variation in transcript expression on identifying differentially expressed genes in RNA-seq analysis". In: *Annals of Human Genetics* 85.6 (2021), pp. 235–244.

[2] Yumei Li et al. "Exaggerated false positives by popular differential expression methods when analyzing human population samples". In: *Genome Biology* 23.1 (2022), p. 79. DOI: 10.1186/s13059-022-02648-4.
