# Supplementary material for: Replicability of bulk RNA-Seq differential expression and enrichment analysis results for small cohort sizes
Source: PLoS Comput Biol. 2025 May 5;21(5):e1011630. doi: 10.1371/journal.pcbi.1011630 (PMC12077797; doi:10.1371/journal.pcbi.1011630)
Supplement: Attachment — Submitted filename: Degen and Medo Response to reviewers 2.pdf [file pcbi.1011630.s006.pdf]

Response to reviewer #4

Peter Degen and Matúš Medo

October 2, 2024

We appreciate the reviewer's comments and thank them for their constructive feedback.

Changes are highlighted in the revised manuscript. The following is a list of the most important changes:

- Added another cancer type (lung squamous cell carcinoma), bringing the total number of TCGA datasets to eight.
- Added five simulated datasets that further strengthen our main conclusions (high precision, low recall of DEGs for small cohort sizes). Results are shown in the supplement (section 2.11).
- Expanded the introduction and added additional references.

1 Detailed response

> 1) Is it true most cancer studies only have a few replicates? The assumption of this entire study is based on a previously published paper [PMID: 30428853] which claimed, "A survey by Baccarella et al. [9] reports that about 50% of 100 randomly selected RNA-Seq experiments with human samples fall at or below six replicates per condition, with this ratio growing to 90% for non-human samples." Unfortunately, Baccarella's paper is flawed. They used only 100 "random" publications to draw this conclusion, selecting low-impact publications and even several papers from the same team. A random check of a few of these papers reveals miscounted sample sizes, ignored technical replicates, and case studies that never performed DE analysis. Even if we assume Baccarella's conclusion is true, it is based on all human studies, with only a few cancer studies included. Given that population heterogeneity is a common issue in cancer research, most responsible cancer studies do not use fewer than six replicates. High-impact publications, clinical trials, and consortium studies typically include dozens of replicates per condition. In reality, non-human and non-cancer studies are more likely to fall below six replicates per condition. Overall, the assumption of this paper is incorrect. The authors spent significant effort solving a non-existent problem.

We thank the reviewer for these insightful comments that motivated us to better articulate the scope and applicability of our study.

We agree that large consortium efforts, such as the TCGA program whose datasets we used as a basis for our study, include many samples. However, there are many preclinical papers in which the number of samples is as low as $n = 3$; two anecdotal examples are the studies by Tuch et al. [1] from the edgeR vignette and by Nisa et al. [2] from our lab. Studies by Schurch et al. [3] and Baccarella et al. [4] map this issue more systematically (though, as the reviewer suggests, maybe

not flawlessly—we would be interested to see if the reviewer perhaps can provide a reference for the critique of the paper by Baccarella et al.?). Beyond RNA-Seq data, many authors have identified inadequate sample sizes as one of the drivers of the replication crisis in science [5, 6]. We thus disagree with the assessment that we addressed a non-existent problem. In effect, our manuscript studies the question of how to assess the results obtained by "irresponsible" studies (using the reviewer's words) that use a few samples from a heterogeneous population to draw their conclusions. Should they be trusted at all? We find that while the sensitivity of these results suffers, high precision can be achieved when the analysis is performed correctly. We also provide practical recommendations.

At the same time, we agree that our original paper title may have invited misinterpretations, as it ends with "... in cancer research". By this, we wanted to express that we used data from cancer research as the basis for our work; this choice was driven by the availability of large RNA-Seq data from cancer research. These datasets allowed us to apply a study design based on subsampling to create many small heterogeneous cohorts that can be further analyzed. To avoid similar misinterpretations going forward, we therefore removed the last three words from the title. This is justified by the addition of simulated, non-cancer datasets (see below) that confirm our observations with cancer data.

2) The comparison between tumor vs. matching normal lacks practical meaning. The authors defined the empirical ground truth based on this comparison. Since tumor cells and normal tissue are essentially different cell types, it is common to see a high number of DEGs ("ground truth"), which is rare in other non-human and non-cancer studies. Previous reviewer 1 mentioned this concern, and the authors merely explained it in their rebuttal letter without adequately addressing it. One possibility is that instead of comparing tumor vs. matching normal, they could compare one subtype vs. another subtype, or poor survival vs. better survival. Comparing two groups of tumor samples will not lead to a huge "ground truth" and high precision. The latter comparison is more relevant in clinical or biological research.

We addressed the high number of ground truth DEGs in our first version of the manuscript by limiting the number of DEGs using a fold change cutoff. As documented using the median expected precision of randomly selected DEGs, precision correctly controlled around 95% is not an inevitable consequence of ground truth size. We also note that the official *edgeR* vignette uses a study by Tuch et al. [1] based on comparing matched normal and tumor tissues with $n = 3$ human patients, thus demonstrating that underpowered studies comparing tumors vs. matching normals are considered an appropriate use case by the authors.

Nonetheless, we appreciate the reviewer's suggestion of adding other types of comparisons, such as tumor subtypes or patient survival. We performed a preliminary analysis comparing lung adenocarcinoma (LUAD) with lung squamous cell carcinoma (LUSC) and found that the number of DEGs is of similar magnitude as comparing matched normal and tumor tissue samples. We also tried patient survival comparisons, however, after stratifying the available patients by clinical variables such as tumor grade and sex, we found that there we were left with an insufficient number of samples. Therefore, these two suggestions would not be practical for our paper.

Instead, we added simulated data as an additional line of evidence to better understand how our results generalize to non-cancer data. The simulated data allows us to tune the strength and prevalence of differential expression in the data, thus directly addressing the reviewer's concern about the number of ground truth DEGs.

> 3) Some confusion in the description of the data. "If the cases came from multiple projects, we kept the patients from the most populated project." As far as I know, no single patient is enrolled in more than one TCGA study.

We acknowledge the ambiguity of phrasing. The intended meaning is that we selected the most populated project in case a cancer site contained more than one project. Specifically, the lung cancer site contains projects for adenocarcinoma (LUAD) and squamous cell carcinoma (LUSC), and we originally selected the former because it has more patients. We have removed this sentence for the revised version, as we now include both LUAD and LUSC in our analysis.

> "To avoid excessive cohort heterogeneity, we finally kept only patients with the most common disease type for the given project." Authors need to provide details about which particular subtype and patient IDs were used in this study. Additionally, they should upload the code to GitHub, along with subsampled patient IDs, to enable reproducibility.

The data and code can be found in the "Data and Code Availability" statement as provided by PLOS Computational Biology (this statement is separate from the manuscript PDF). Our GitHub repository (https://doi.org/10.5281/zenodo.8333519) contains a Jupyter notebook that can be used to download the data from TCGA. For the revised manuscript, we have also uploaded the metadata with patient IDs to the GitHub repository. The cancer subtypes can also be found in Table 1 in the original manuscript.

> 4) Authors ignored other confounding factors previously mentioned by reviewers. TCGA studies is too complicated. They should include simulated data, non-human data, and non-TCGA data to better understand the utility of this study.

Patient-specific confounders mentioned by reviewer 3 ("gender, sex") are already taken into account by the paired-sample experimental design with patient as a factor. This is confirmed by Gordon Smyth, one of the co-authors of the *edgeR* package, in a BioConductor post: *"Adjusting for patient automatically adjusts also for gender, age and any other personal characteristics."* It is not possible to add further patient-specific factors to the model, as the design matrix will not be full rank, resulting in a runtime error when performing differential expression analysis.

To further investigate the generalizability of our results, we added results from simulated datasets to the revised manuscript. The simulated data affirms our main conclusion that low-powered RNA-Seq studies can still achieve high precision despite low replicability, the latter being a consequence of low recall. Notably, this holds even when the number of differentially expressed genes in the ground truth is very low (1% of all genes). See second row in Supplementary Fig. 30.

> 5) This paper is too similar to Cui et al.'s paper [PMID: 35876281], with only a few more TCGA datasets. If this paper is merely an extension of a previous publication with slightly more assessments and data leading to very similar conclusions, it lacks novelty as a new publication.

Our work and conclusions go beyond Cui et al. [7] in many ways. Whereas Cui et al. caution that underpowered cancer studies are unreliable due to low replicability, our main result is that their precision can be high despite low replicability and low sensitivity. We also show that effect sizes are inflated, but this does not reduce the precision if the proper fold change thresholding method

is used. Compared to Cui et al., we evaluated two popular tools for DEG analysis, various statistical tests, two different ways of fold change thresholding, considered both paired and unpaired study designs, and used several evaluation metrics on eight different TCGA datasets, two non-TCGA datasets, and five simulated datasets. Furthermore, our subsampling methodology is different from Cui et al., allowing us to substantially increase the number of subsampled cohorts. We note that evidence synthesis from different approaches and independent teams is an important step in the validation of scientific results [8–10].

References

[1] Brian B Tuch, Rebecca R Laborde, Xing Xu, Jian Gu, Christina B Chung, Cinna K Monighetti, Sarah J Stanley, Kerry D Olsen, Jan L Kasperbauer, Eric J Moore, et al. "Tumor transcriptome sequencing reveals allelic expression imbalances associated with copy number alterations". In: *PloS one* 5.2 (2010), e9317.

[2] Lluís Nisa, David Barras, Michaela Medová, Daniel M Aebersold, Matúš Medo, Michaela Poliaková, Jonas Koch, Beat Bojaxhiu, Olgun Eliçin, Matthias S Dettmer, et al. "Comprehensive genomic profiling of patient-matched head and neck cancer cells: a preclinical pipeline for metastatic and recurrent disease". In: *Molecular cancer research* 16.12 (2018), pp. 1912–1926.

[3] N.J. Schurch, P. Schofield, M. Gierliński, C. Cole, A. Sherstnev, V. Singh, N. Wrobel, K. Gharbi, G.G. Simpson, T. Owen-Hughes, et al. "How many biological replicates are needed in an RNA-seq experiment and which differential expression tool should you use?" In: *RNA* 22.6 (2016). DOI: 10.1261/rna.053959.115.

[4] A. Baccarella, C.R. Williams, J.Z. Parrish, and C.C. Kim. "Empirical assessment of the impact of sample number and read depth on RNA-Seq analysis workflow". In: *BMC Bioinformatics* 19 (2018), p. 423. DOI: 10.1186/s12859-018-2445-2.

[5] Andrew D. Higginson and Marcus R. Munafò. "Current Incentives for Scientists Lead to Underpowered Studies with Erroneous Conclusions". In: *PLOS Biology* 14.11 (Nov. 10, 2016). Publisher: Public Library of Science, e2000995. ISSN: 1545-7885. DOI: 10.1371/journal.pbio.2000995. URL: https://journals.plos.org/plosbiology/article?id=10.1371/journal.pbio.2000995 (visited on 07/08/2024).

[6] J.P. Ioannidis. "Why most published research findings are false". In: *PLoS Medicine* 2.8 (2005), e1004085. DOI: 10.1371/journal.pmed.0020124.

[7] W. Cui, H. Xue, L. Wei, J. Jin, X. Tian, and Q. Wang. "High heterogeneity undermines generalization of differential expression results in RNA-Seq analysis". In: *Human Genomics* 15.7 (2021). DOI: 10.1186/s40246-021-00308-5.

[8] Marcus R. Munafò and George Davey Smith. "Robust research needs many lines of evidence". In: *Nature* 553.7689 (Jan. 2018). Bandiera_abtest: a Cg_type: Comment Publisher: Nature Publishing Group Subject_term: Research management, Philosophy, pp. 399–401. DOI: 10.1038/d41586-018-01023-3. URL: https://www.nature.com/articles/d41586-018-01023-3 (visited on 07/12/2024).

[9] Eric-Jan Wagenmakers, Alexandra Sarafoglou, and Balazs Aczel. "One statistical analysis must not rule them all". In: *Nature* 605.7910 (May 2022). Bandiera_abtest: a Cg_type: Comment Publisher: Nature Publishing Group Subject_term: Publishing, Research management, pp. 423–425. DOI: 10.1038/d41586-022-01332-8.

[10] Andrew Gelman and Eric Loken. “The Statistical Crisis in Science”. In: *The Statistical Crisis in Science*. Princeton University Press, Jan. 12, 2016, pp. 305–318. ISBN: 978-1-4008-7337-1. DOI: 10.1515/9781400873371-028. URL: https://www.degruyter.com/document/doi/10.1515/9781400873371-028/html (visited on 08/09/2024).
