# Supplementary material for: Replicability of bulk RNA-Seq differential expression and enrichment analysis results for small cohort sizes
Source: PLoS Comput Biol. 2025 May 5;21(5):e1011630. doi: 10.1371/journal.pcbi.1011630 (PMC12077797; doi:10.1371/journal.pcbi.1011630)
Supplement: Attachment — Submitted filename: Degen and Medo response to reviewers 3.pdf [file pcbi.1011630.s007.pdf]

Replicability of bulk RNA-Seq differential expression and enrichment analysis results

Response to the reviewers

Peter Degen and Matúš Medo

February 25, 2025

Summary of the most important changes

- We extended the number of data sets in the main text from 8 to 18. The 10 new data sets comprise 6 cancer subtype comparisons and 4 non-cancer data sets.
- We overhauled the main text figures to focus on differences between data sets instead of differences between statistical methods (moving the latter to the supplement).
- The substantial expansion in the number of data sets required us to make cuts in other places, which helped to streamline the final manuscript. The following results were cut:
  - The enrichment analysis is now restricted to one tool (GSEApy), removing ClusterProfiler and over-representation analysis.
  - The impact of outlier sample removal was removed.
  - The simulated data part was removed from the supplement.
- The increased heterogeneity of the analyzed data sets allowed us to add a new experiment that uses bootstrap resampling to estimate the expected precision and replicability of DEGs and enriched terms for a given data set. This is a practical addition that can help researchers working with RNA-Seq data limited by sample size.

Response to reviewer #4

Thank you for your comments and for engaging in a discussion of the replicability of RNA-Seq data analysis. We have further revised our manuscript (all substantial changes are highlighted with color). We want to stress that your previous assessment “The authors made only slight modifications...” was probably caused by our decision to place new results in Supporting Information. This has been changed now and new results are fully integrated with the main text. We have also further extended the results as described in detail in the following paragraphs.

The reviewer was right to suspect that adding a wider variety of data sets may reveal additional patterns in our results. By expanding the number of data sets in the main text from 8 to 18, we have observed greater variability in possible outcomes and have changed the manuscript to highlight these new findings accordingly. Notably, we have added an entirely new experiment based on bootstrap resampling to predict performance metrics such as replicability and precision from individual low-powered data sets.

> 1. Regarding Bacarella’s paper, please carefully review ‘Additional File 4’ and consider the probability of randomly drawing 100 papers from PubMed, with multiple papers coming from the same group. This suggests it’s not truly random.

Thank you for clarifying the issues that you have with the study by Baccarella et al. According to the authors, the studies were selected at random and subsequently reviewed to meet the author’s inclusion criteria. We checked the mentioned ’Additional File 4’ programmatically and found that 95% (998 of 1054) of all authors, 95% of the last authors, and 96% of the first authors are unique in the sample of 100 papers analyzing human RNA-Seq data. These numbers do not cast substantial doubt on the sampling procedure described by the authors.

> 2. In Bacarella’s paper, they specifically stated, ’Studies utilizing previously published datasets, including large-scale sequencing efforts (such as TCGA) were excluded, to ensure a representative sampling of the most common experimental designs.’ It’s reasonable to exclude large consortia studies and publications using large cohorts of data for their own purpose. However, you cannot conclude that most cancer studies use fewer than six replicates.

We do not conclude that most cancer studies use fewer than six replicates. We only use the referenced literature to show that studies with a small number of replicates are common, which motivates our own investigation of the impact on the differential expression analysis.

3. Once again, comparing tumor versus normal tissue doesn't make practical sense. When inter-group signals are much stronger than intra-group variability, it doesn't matter whether you have 6 replicates or 100. Consider an extreme case: comparing humans and E. coli—you wouldn't need replicates at all. You cannot use such extreme cases to imply your method works across all human studies. Again, comparing tumor to normal tissue is inappropriate.

To address this comment, we added four non-TCGA data sets (tuberculosis, pulmonary fibrosis, human placenta, and yeast mutation) and six cancer subtype comparisons. Regarding the signal strength, Figure 2 in the main text shows that the number of identified DEGs grows with the number of replicates for all data sets, including the TCGA normal tissue vs tumor comparisons. This suggests that biological variability is sufficient to make differential expression analysis non-trivial for all analyzed data sets.

4. In real-world research, scientists and clinicians are more focused on comparing different subtypes rather than tumor versus normal within a single cancer type.
6. There are many human studies that don't have as many samples as TCGA. At least demonstrate one or two such examples, rather than relying solely on TCGA data in the main text.
8. The tumor vs. normal comparison across eight cancers only demonstrates a single scenario. One scenario cannot convince people of the broader applicability of your method.

We do not know which comparisons are most common in the differential expression analysis of RNA-Seq data but newly added data cancer subtype comparisons and non-TCGA data sets follow your suggestion of extending our scope beyond the TCGA normal-tumor comparisons. We decided to keep also the normal tissue-tumor comparisons as many studies involve such analyses [1–10], which shows that our results based on comparing normal tissue and tumor samples are also relevant. Their inclusion also increases the diversity of the analyzed data sets which is important for the newly added results on estimating the expected precision and replicability of DEGs and enriched terms.

5. The authors also misunderstood the term 'subtype.' In cancer biology, subtypes refer to groups of the same cancer sharing specific characteristics, such as the four breast cancer subtypes: Luminal A, Luminal B, HER2-positive, and triple-negative. LUSC, LUAD, and BRCA are different cancers, not subtypes. Don't argue that LUSC and LUAD are both 'lung cancer'—they are distinct cancers. The 33 cancer types studied by the TCGA consortium are clearly listed on the NCI's website. Please follow their definitions.

We acknowledge the mistaken phrasing in the previous rebuttal letter. The main text does not refer to any of the cancer types as subtypes and correctly refers to LUSC and LUAD as different cancer types.

7. Even if you insist on using TCGA data, there are more meaningful ways to apply it. For example, comparing the four molecular subtypes in BRCA, or comparing patients with good vs. poor survival outcomes in triple-negative BRCA, or platinum-tolerant vs. resistant patients in triple-negative BRCA.

Thank you for this suggestion. The revised version now includes all six BRCA subtype comparisons obtained from the four molecular subtypes (basal-like, luminal A, luminal B, Her2+).

References

[1] Olivia M Marx, Marc M Mankarious, Walter A Koltun, and Gregory S Yochum. "Identification of differentially expressed genes and splicing events in early-onset colorectal cancer". In: *Frontiers in Oncology* 14 (2024), p. 1365762.

[2] Myeong Ho Jung, Sung Chun Kim, Geoung A Jeon, Seung Hyun Kim, Youngyul Kim, Kyoung Suk Choi, Sang Ick Park, Myung Kuk Joe, and Kuchan Kimm. "Identification of differentially expressed genes in normal and tumor human gastric tissue". In: *Genomics* 69.3 (2000), pp. 281–286.

[3] Yongjin Luo, Zhen Liu, and Xiaoxia Hu. "ceRNA Network and WGCNA Analyses of Differentially Expressed Genes in Cervical Cancer Tissues for Association with Survival of Patients". In: *Reproductive Sciences* (2024), pp. 1–11.

[4] Ting Lan, Yuxiang Yan, Dali Zheng, and Lincan Ding. "Investigating diagnostic potential of long non-coding RNAs in head and neck squamous cell carcinoma using TCGA database and clinical specimens". In: *Scientific Reports* 14.1 (2024), p. 7500.

[5] Rong Jia, Zhongxian Li, Wei Liang, Yucheng Ji, Yujie Weng, Ying Liang, and Pengfei Ning. "Identification of key genes unique to the luminal a and basal-like breast cancer subtypes via bioinformatic analysis". In: *World journal of surgical oncology* 18 (2020), pp. 1–11.

[6] Ga-Eon Kim, Nah Ihm Kim, Ji Shin Lee, Min Ho Park, and Keunsoo Kang. "Differentially expressed genes in matched normal, cancer, and lymph node metastases predict clinical outcomes in patients with breast cancer". In: *Applied Immunohistochemistry & Molecular Morphology* 28.2 (2020), pp. 111–122.

[7] Andrew M Gross, Jason F Kreisberg, and Trey Ideker. "Analysis of matched tumor and normal profiles reveals common transcriptional and epigenetic signals shared across cancer types". In: *PloS one* 10.11 (2015), e0142618.

[8] Bing Gao, Shufang Ning, Jilin Li, Haizhou Liu, Wene Wei, Feixiang Wu, Yanping Tang, Yan Feng, Kezhi Li, and Litu Zhang. “Integrated analysis of differentially expressed mRNAs and miRNAs between hepatocellular carcinoma and their matched adjacent normal liver tissues”. In: *Oncology Reports* 34.1 (2015), pp. 325–333.

[9] Brian B Tuch, Rebecca R Laborde, Xing Xu, Jian Gu, Christina B Chung, Cinna K Monighetti, Sarah J Stanley, Kerry D Olsen, Jan L Kasperbauer, Eric J Moore, et al. “Tumor transcriptome sequencing reveals allelic expression imbalances associated with copy number alterations”. In: *PloS one* 5.2 (2010), e9317.

[10] Paolo GV Martini, Deanne M Taylor, Jadwiga Bienkowska, Jennifer Jackson, Gregory McAllister, Heike Keilhack, and Robert K Campbell. “Comparative expression analysis of four breast cancer subtypes versus matched normal tissue from the same patients”. In: *The journal of steroid biochemistry and molecular biology* 109.3-5 (2008), pp. 207–211.
